# Design, synthesis, and biological properties exhibited by 1,2,3-triazole based Grp94-selective inhibitors

**DOI:** 10.1016/j.ejmech.2025.118394

**Published:** 2025-11-29

**Authors:** Hao Xu, Dustin J.E. Huard, Elijah Dunn, Lucas A. Chalfoun, Felix Adulley, Raquel L. Lieberman, Brian S.J. Blagg

**Affiliations:** a Department of Chemistry and Biochemistry, Warren Center for Drug Discovery, The University of Notre Dame, 305 McCourtney Hall, Notre Dame, IN, 46556, USA; b School of Chemistry & Biochemistry, Georgia Institute of Technology, Atlanta, GA, 30332, USA

**Keywords:** Heat shock protein 90 (Hsp90), Glucose regulated-protein 94 (Grp94), Mutant myocilin, Open-angle glaucoma, Integrin, Metastatic cancer

## Abstract

Grp94, the endoplasmic reticulum-resident paralog of Hsp90, is responsible for the folding and maturation of several client proteins including integrins and mutant myocilin. Inhibition of Grp94 with small molecules has been shown to reduce cell migration of breast cancer cells and promote degradation of mutant myocilin aggregates. Herein, we describe the development of 1,2,3-triazole based Grp94-selective inhibitors derived from a nitrogen scan on **BnIm**. Structure-activity relationship studies identified lead compound **47**, which manifests **76** nM affinity for Grp94 with 121-fold selectivity over Hsp90α. In cellular studies, compound **47** induced the degradation of integrin α2 in MDA-MB-231 cells and reduced intracellular accumulation of mutant myocilin in human trabecular meshwork cells. These findings supported compound **47** as a potent and selective Grp94-selective inhibitor with therapeutic potential.

## 1. Introduction

Heat shock proteins (Hsps) are evolutionarily conserved molecular chaperones that maintain cellular homeostasis by regulating the maturation of client protein substrates [1]. In addition to constitutive expression under normal cellular growth, Hsps are upregulated in response to various cellular stresses such as elevated temperature and malignant transformation [2]. The 90 kDa heat shock proteins (Hsp90s) are the most abundant chaperones in eukaryotes amongst the five major Hsp family members. Hsp90, along with its various co-chaperones, facilitates the maturation, activation and stabilization of more than 400 Hsp90-dependent client proteins, many of which are associated with the ten hallmarks of cancer [3–5]. Hsp90 functions as a homodimer and the N-terminal domain contains a Bergerat fold that is an unique and druggable ATP-binding site, which distinguishes it from other ATPases and allows for the development of selective inhibitors [6].

More than 20 Hsp90 N-terminal inhibitors such as 17-AAG, SNX-5422, and AUY-922 entered clinical trials for the treatment of cancer. Unfortunately, most of them failed due to cardio-, ocular- and/or dose-limiting toxicities amongst other concerns [7,8]. These detriments result from inhibition of hERG assembly, disruption of photoreceptor trafficking and induction of the heat shock response (HSR). The HSR is produced by pan-inhibition of all four Hsp90 isoforms: Hsp90α and Hsp90β, glucose regulated protein 94 (Grp94), and tumor necrosis factor receptor associated protein 1 (TRAP1). Moreover, all four isoforms share >85 % identity within the N-terminal ATP-binding site making the selective inhibition of individual paralogs extremely difficult. In eukaryotes, two genes encode the inducible and constitutively expressed isoforms, Hsp90α and Hsp90β, respectively, both of which are localized to the cytosol, whereas Grp94 and TRAP1 are found in the endoplasmic reticulum (ER) and mitochondria, respectively. Thus, the development of isoform-selective Hsp90 inhibitors may provide an opportunity to overcome these detriments.

Grp94 is the most structurally unique Hsp90 isoform, which makes it a promising target for the development of selective ATP-competitive inhibitors. This lowered identity compared to other isoforms is the result of a 5-amino acid insertion in the primary sequence, which produces a unique secondary binding pocket that exists exclusively in Grp94 [9,10]. Several Grp94-dependent substrates have been elucidated, including toll-like receptors, integrins and mutant myocilin. However, in general, Grp94 inhibition is not toxic nor does it exhibit anti-proliferative activity. Additionally, Grp94 is only essential during embryonic development, but non-essential for developed cells [11].

Grp94 is a promising target for the development of agents to treat open-angle glaucoma caused by the intracellular accumulation of mutant myocilin [12–15]. Myocilin is expressed in the anterior eye tissue called the trabecular meshwork (TM). Inherited pathogenic mutations in *myoc*-OLF lead to mutant myocilin misfolding. Mutant myocilin accumulates within the ER of TM cells due to inefficient ER-associated degradation (ERAD) clearance [16]. Grp94 was identified as a key chaperone that prevents mutant myocilin clearance [16] by accelerating co-aggregation with mutated myocilin [17,18]. Upon Grp94 knockdown or by chemical inhibition of Grp94, clearance of mutant myocilin was promoted via autophagy [16]. Grp94 inhibition reduced intracellular mutant myocilin levels but had no adverse effect on WT myocilin [16, 17]. Thus, Grp94-selective inhibitors represent a promising treatment for myocilin-associated glaucoma.

**Fig. 1.**
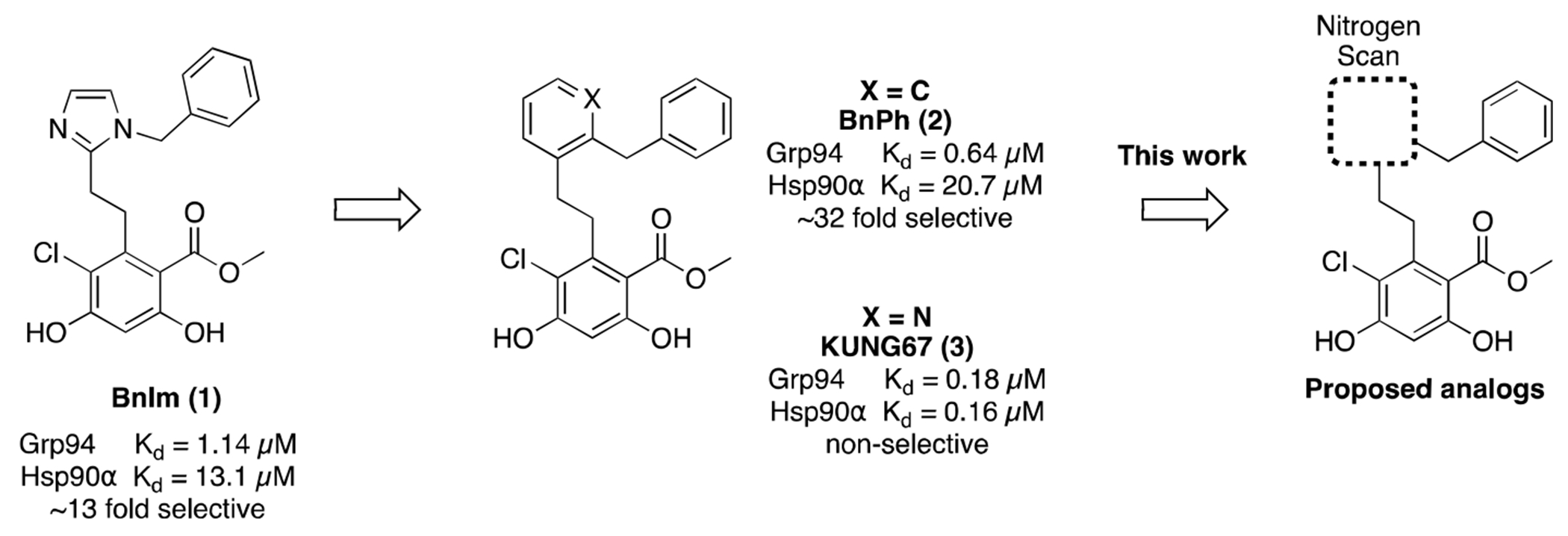
First and second generations of Grp94 inhibitors.

Herein, we report the discovery of next generation Grp94-selective inhibitors. A nitrogen scan of **BnIm (1)** identified the triazole ring system as a preferred scaffold for optimization (Fig. 1). Subsequent structure-activity relationship (SAR) studies resulted in a lead compound **47**, which manifested a 76 nM affinity and 121-fold selectivity over Hsp90α. Compared to the previously reported first and second generation **BnIm** and **BnPh**, compound **47** exhibited 15-fold and 8-fold improvements in affinity, respectively.

## 2. Results and discussion

### 2.1. Design and synthesis of Grp94-selective inhibitors via nitrogen scan

Two generations of Grp94-selective inhibitors have been previously disclosed: **BnIm (1)**, which features an imidazole linker between the resorcinol and phenyl appendages, and **BnPh (2)**, which incorporates a phenyl linker (Fig. 1) [13,19]. **BnIm (1)** exhibited a 1.14 μM affinity for Grp94 and ~13-fold selectivity over Hsp90α in fluorescence polarization (FP) assays, whereas replacement of the imidazole moiety with a phenyl ring increased both affinity and selectivity. Structure-activity relationship (SAR) studies on the phenyl linker of **BnPh (2)** led to incorporation of a 3-pyridyl ring (**KUNG67**), which produced in a significant increase in affinity (K_d_ = 0.18 μM). However, incorporation of N-heteroatoms into the phenyl linker yielded compounds with little to no selectivity versus other isoforms. It was clear that the inclusion of a nitrogen atom significantly increased binding to Grp94 as well as to the other isoforms. Furthermore, prior studies highlighted the enhanced ability of **BnIm** derivatives to promote mutant myocilin degradation in *in-vitro* glaucoma models as compared to **BnPh** analogs [12,15,19]. Therefore, this study aimed to determine whether the affinity and/or selectivity of the **BnIm** scaffold could be improved by the inclusion of additional nitrogens within the linker.

**Scheme 1.**
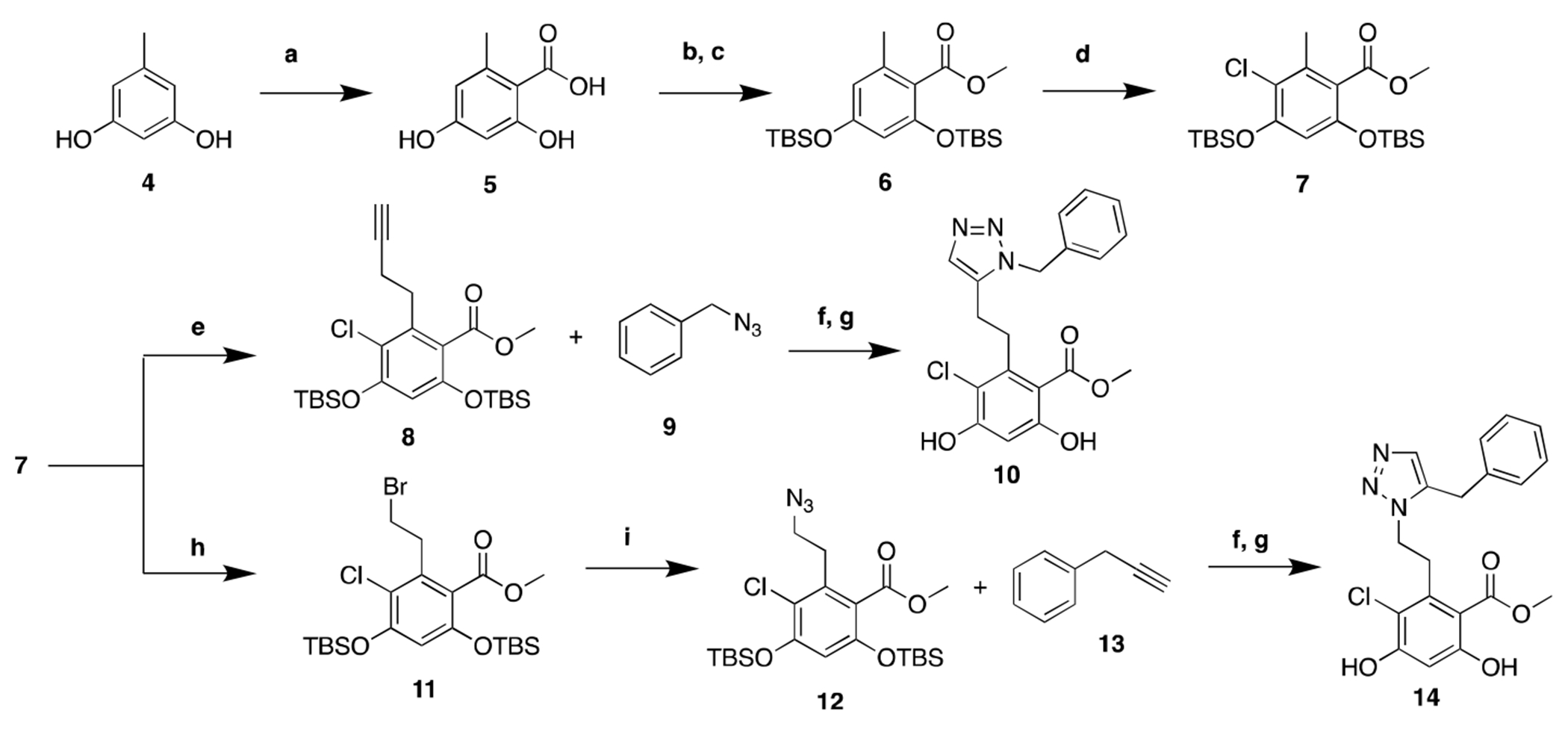
Synthesis of triazole based analogs **10** and **14**. **a)**. DBU, CO_2_, MeCN, rt, 2 d; **b)**. Mel, NaHCO_3_, Acetone, rt, 16hr; **c)**. TBSCl, imidazole, DMF, rt, 12 h; **d)**. SO_2_Cl_2_,THF, −40 °C, 30 min; **e)**. LDA, propargyl bromide, THF, −78 °C, 30 min; **f)**. Cp*RuCl(PPh_3_)_2_, 1,4-dioxane, 60 °C, 16 h; **g)**. TBAF, THF, rt, 2 h; **h)**. LDA, dibromomethane, THF, −78 °C, 30 min; **i)**. NaN_3_, DMF, 80 °C, 12 h.

The increased affinity observed with **KUNG67 (3)** appeared to result from a hydrogen bonding interaction with Asn162 within the Grp94 binding site. Therefore, we designed **BnIm** derivatives **10** and **14** (Scheme 1), which were based on the inclusion of an additional nitrogen atom in the imidazole. This modification results in formation of a triazole ring system. These derivatives were prepared to investigate the influence of an additional nitrogen atom as well as the location of nitrogen with regards to both affinity and selectivity. Synthesis of **10** and **14** began via Kolbe-Schmitt carboxylation of commercially available resorcinol (**4**), followed by methylation of the resulting acid and TBS protection of the resorcinol to afford **6**. Regioselective chlorination was achieved upon treatment of **6** with sulfuryl chloride (SO_2_CI_2_) at −40 °C to yield resorcinol **7**, which was used as a key intermediate for subsequent derivatives. Alkylation of **7** with propargyl bromide produced the terminal alkyne, **8**. A ruthenium-catalyzed azide-alkyne cycloaddition reaction (RuAAC) between **8** and benzyl azide **9** followed by subsequent deprotection yielded the 1,5-disubstituted triazole, **10**. In contrast, alkylation of resorcinol **7** with dibromomethane (DBM) furnished alkyl bromide **11**, which underwent nucleophilic substitution with sodium azide (NaNa) to afford intermediate **12**. A ruthenium-catalyzed cycloaddition reaction with **13** afforded the 1,5-disubstituted triazole regioisomer, **14**.

**Scheme 2.**
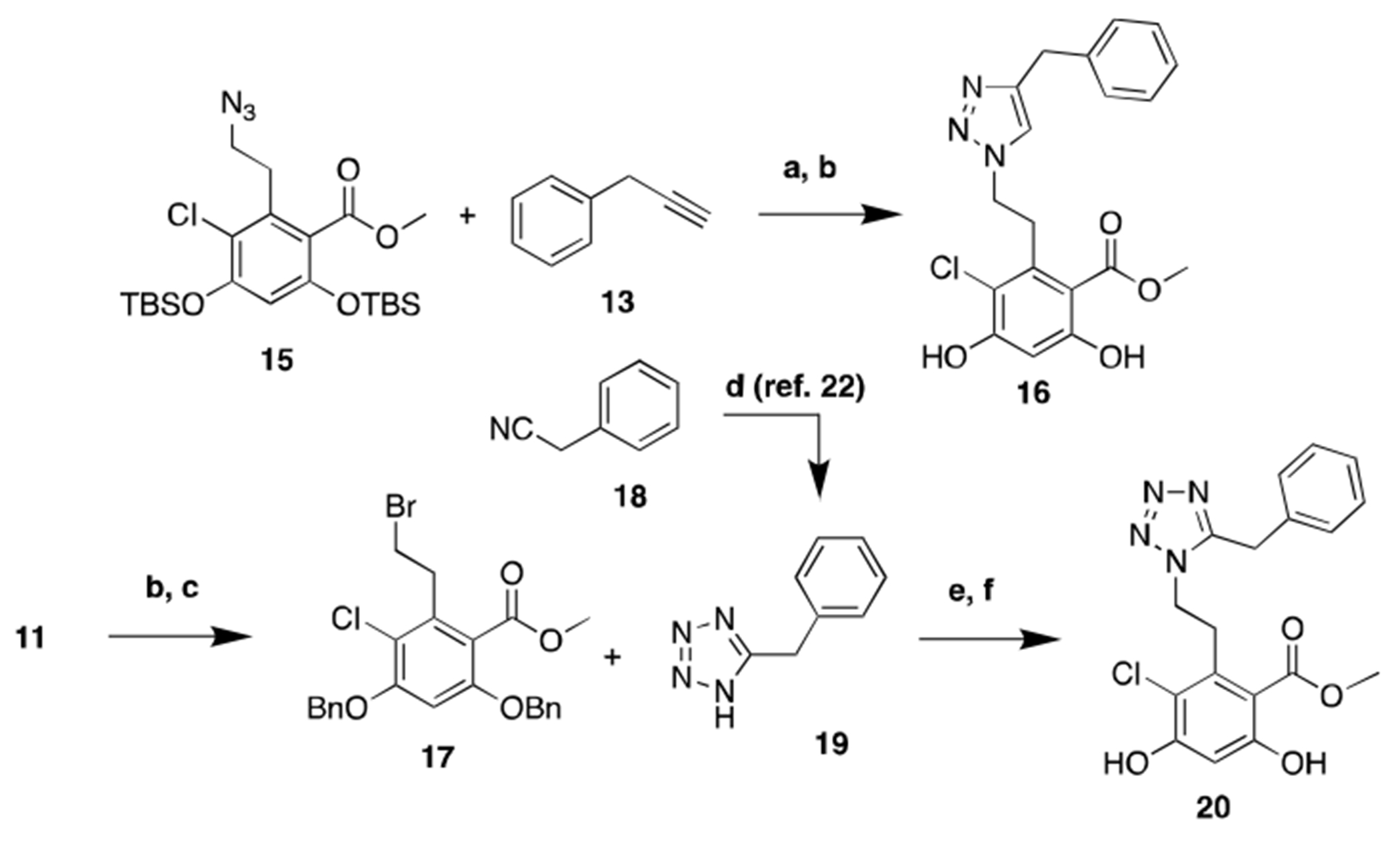
Synthesis of triazole based analog **16** and 1-*H*-tetrazole based analog **20**. **a)**. CuSO_4_*5H_2_O, sodium ascorbate, THF/H_2_O, 80 °C, 12 h; **b)**. TBAF, THF, rt, 2 h; **c)**. BnBr, K_2_CO_3_, DMF, rt, 16 h; **d)**. TBAF*3H_2_O, TMSN_3_, 120 °C, 24 h.^22^ **e)**. K_2_CO_3_, DMF, 60 °C, 16 h; **f)**. Pd/C, H_2_, EtOAc, rt, 12 h.

Compounds **10** and **14** were evaluated by a competitive binding FP assay to determine the apparent binding affinity for Grp94 versus Hsp90α (Table 1). Both of the 1,5-disubsititued compounds exhibited increased affinity towards Grp94 relative to **BnIm (1)**, with apparent K_d_’s of 0.57 μM and 0.69 μM, respectively. However, compound **14** manifested decreased selectivity. Therefore, we opted to investigate both 1,4-disubstituted triazole structure **16** and the 1-H-tetrazole structure **20**. Preparation of **16** and **20** is described in Scheme 2 in which a copper-catalyzed azide-alkyne cycloaddition reaction (CuAAC) between intermediate **15** and prop-2-yn-1-ylbenzene (**13**) yielded the 1,4-disubstituted triazole, **16**. Meanwhile, synthesis of the 1-*H*-tetrazole analog **20** was achieved from intermediate **11**, which was converted to the resorcinolic bis-benzyl ether, **17**. Commercially available benzyl nitrile (**18**) was treated with trimethylsilyl azide (TMSN_3_) and TBAF, which led to formation of benzyl-1-*H*-tetrazole (**19**).^22^ A subsequent nucleophilic substitution reaction between **17** and **19** afforded the 1-*H*-tetrazole derivative, **20**. Evaluation of the 1,4-disubstituted triazole analog **16** revealed reduced affinity and selectivity towards Grp94 (Table 1). In addition, the 1-*H*-tetrazole analog **20** exhibited similar affinity to triazole derivatives **10** and **14**; however, it lost selectivity when compared to triazole, **14**. On the basis of these observations, we conducted structure-activity relationship (SAR) studies that focused on the benzyl side chain of compound **14** for further optimization.

### 2.2. Structure-activity relationship studies of compound 14

**Scheme 3.**
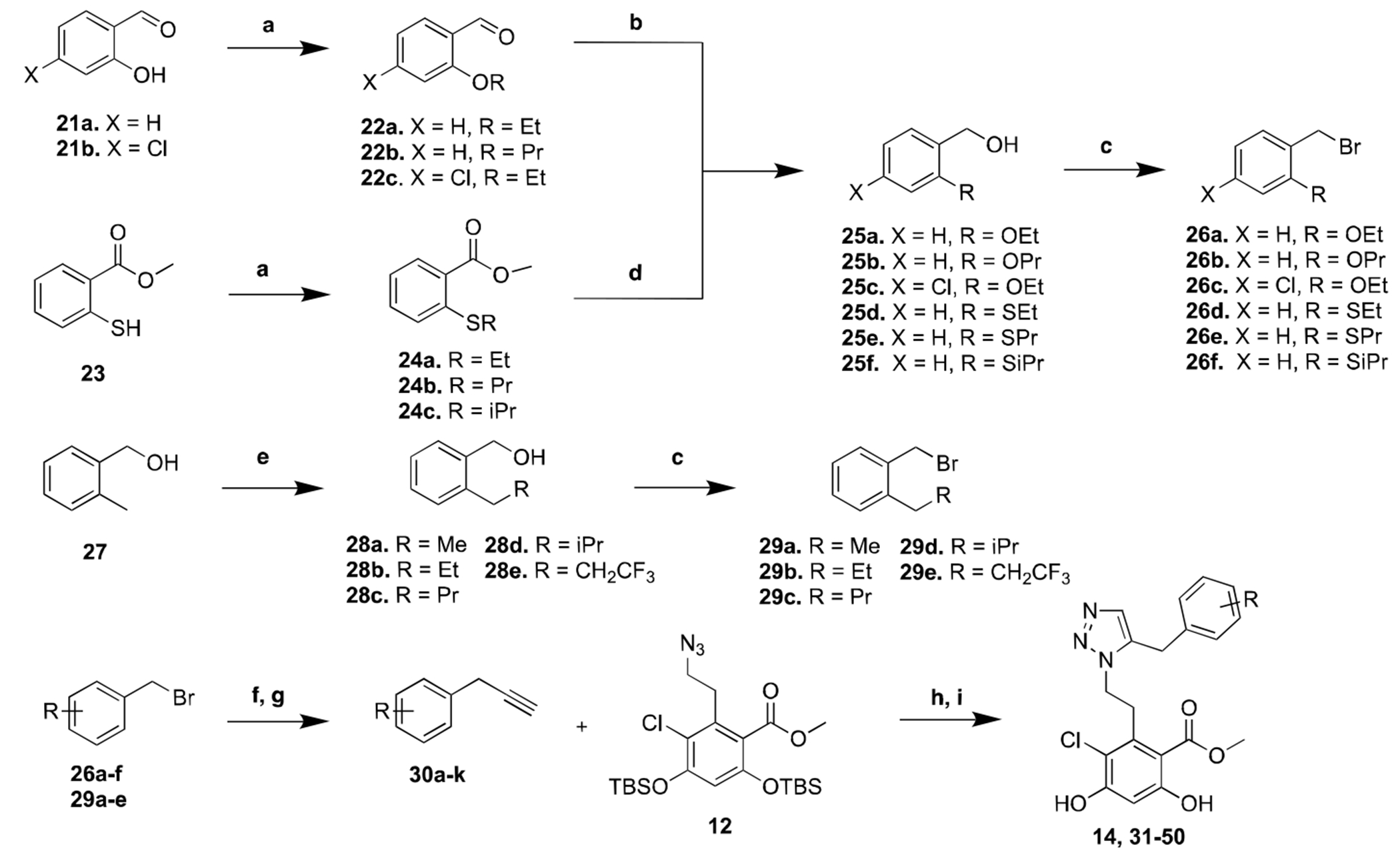
Synthesis of compund **14** analogs, **a)**, alkyl iodide, K_2_CO_3_, DMF, rt, 12 h; **b)**. NaBH_4_, EtOH, rt, 5 h; **c)**. PBr_3_,DCM, rt, 3 h; **d)**. LiAlH_4_, Et_2_O, 0 °C - rt, 3 h; **e)**. 1. nBuLi, Et_2_O, reflux, 4 h; 2. alkyl iodide, Et_2_O, rt, 1 h; **f)**. iPrMgCl, TMS acetylene, CuBr, THF, 70 °C, 5 h; **g)**. K_2_CO_3_, MeOH, rt, 2 h; **h)**. Cp*RuCl(PPh_3_)_2_,1,4-dioxane, 60 °C, 16 h; **i)**. TBAF, THF, rt, 2 h.

Based on the preparation of **14**, various substituted benzyl alkynes were pursued to rapidly elucidate SAR for this scaffold. Therefore, a series of analogs substituted with ethers, thioethers, or alkyl moieties were sought, and synthesized as depicted in Scheme 3. First, commercially available salicylaldehydes, **21a** and **21b**, or 2-mercaptobenzoic ester, **23**, were alkylated with alkyl halides to produce the corresponding aryl ethers, **22a-c**, or thioethers, **24a-c**, respectively. Next, **22a-c** underwent a reduction mediated by sodium borohydride (NaBH_4_), while **24a-c** were reduced with lithium aluminum hydride (LiAlH_4_), to give alcohols, **25a-f**, which were subsequently converted to the benzyl bromides **26a-f** via phosphorus tribromide (PBr_3_). 2-Methyl benzyl alcohol (**27**) underwent benzyl alkylation with various alkyl iodides to generate benzyl alcohols **28a-e**. Similarly, **28a-e** were treated in PBr_3_ to afford **29a-e**. Terminal alkynes **30a-k** were prepared via a copper-catalyzed addition of the Grignard derived from trimethylsilyl (TMS) acetylene with the corresponding benzyl bromides, followed by removal of the TMS group. Finally, a ruthenium-catalyzed cycloaddition between **12** and **30a-u**, followed by removal of the TBS groups yielded a series of the 1,5-disubstituted triazoles analogs, **31–50**.

SAR studies on the **BnIm** scaffold revealed that optimization of the benzyl side chain at both 2- and 4-positions were effective at Grp94 inhibition [19,20]. Substitutions at the 4-position were designed to extend deeper into the hydrophobic pocket while mitigating metabolic liabilities. Compounds **31** to **33**, which contain a halogen substitution at the 4-position exhibited comparable affinity and selectivity as **14**. Introduction of a 4-methyl group **(34**, see Table 2) manifested similar affinity/selectivity, whereas extension to a 4-ethyl substituent **(35)** resulted in the loss of selectivity. As anticipated, polar substitutions such as the 4-methoxyl derivative **36** and alteration of the 3-position **(37, 38)** did not afford compounds with increased Grp94 affinity. Therefore, next we focused on substitutions at the 2-position. For example, incorporation of a 2-ethoxyl group **(40)** increased Grp94 affinity to 0.3 μM along with 30-fold selectivity. Compound 41 was proposed to combine the beneficial properties of substituents at both the 2- and 4-positions. However, the combination of 4-Cl with 2-ethoxy resulted in decreased affinity. Extending the ethyl ether chain to the propyl ether (**42**) without modifications at the 4-position led to enhanced affinity (K_d_ = 85 nM), providing evidence of a hydrophobic subpocket within this region of the ATP-binding site.

**Fig. 2.**
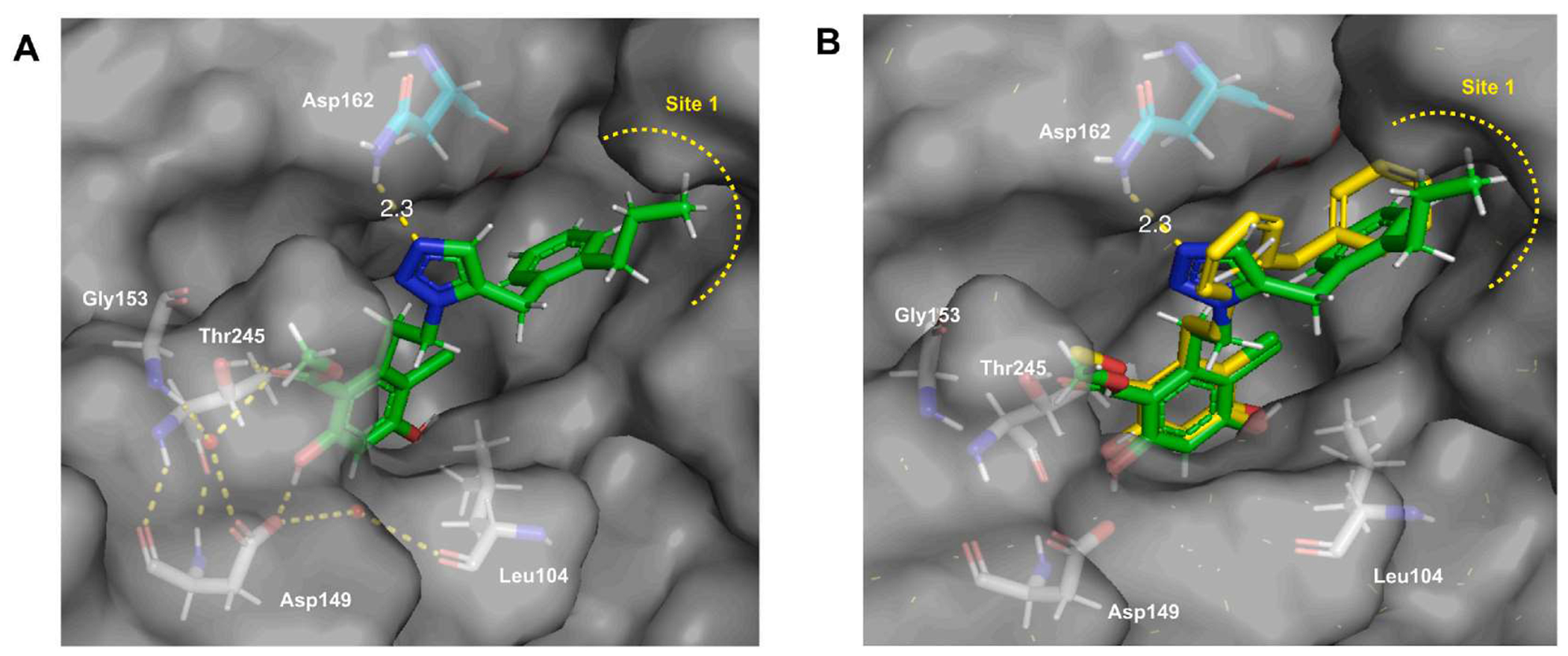
(A) Predicted binding mode of compound **47** bound to the Grp94 N-terminus (PDB: 6AOL). (B) Overlay of compound **47** and **BnPh** bound to Grp94 (PDB: 6AOM).

Replacement of the ether with a thioether (compound **43** to **45**) provided additional insights. The thiophenol ether exhibits a weaker hydrogen bond acceptor due to the larger atomic size of sulfur relative to oxygen, and instead it provided additional hydrophobicity. Compound **43** exhibited a similar affinity to **40**, whereas **44** displayed higher affinity (K_d_ = 56 nM with 78-fold selectivity) when compared to **42**. The loss of affinity for **45** indicated an intolerance to bulky substituents. Based on these observations, we introduced hydrocarbon chains that lack polarity at the 2-position. Extension of an ethyl to a propyl group resulted in a 10-fold increase in affinity (compound **47**, K_d_ = 76 nM and 121-fold selectivity). In contrast, the introduction of a butyl or iso-butyl group (**48, 49**) led to decreased selectivity. Compound **50** was prepared for comparison to the propyl analog **47**, but the affinity remained comparable, and the selectivity was lowered. The predicted binding mode of **47** revealed that the resorcinol ring is stabilized within the N-terminal binding pocket of Grp94 via a hydrogen-bonding network with Gly153, Thr245, Asp149, and Leu104. The triazole ring appears to form direct interactions with Asn162 via a contact distance of approximately 2.3 Å (Fig. 2A), which minimizes rotation of the ethylene linker. Overlay of compound **47** and **BnPh** suggests this hydrogen-bond interaction induces in a slight rotation of the linker ring to project the benzylic side chain towards hydrophobic site 1 (Fig. 2B). The 2-propyl substituent further extends into site 1, which appears to occupy the pocket more completely than **BnPh**.

### 2.3. Cellular evaluation of compound 47

**Fig. 3.**
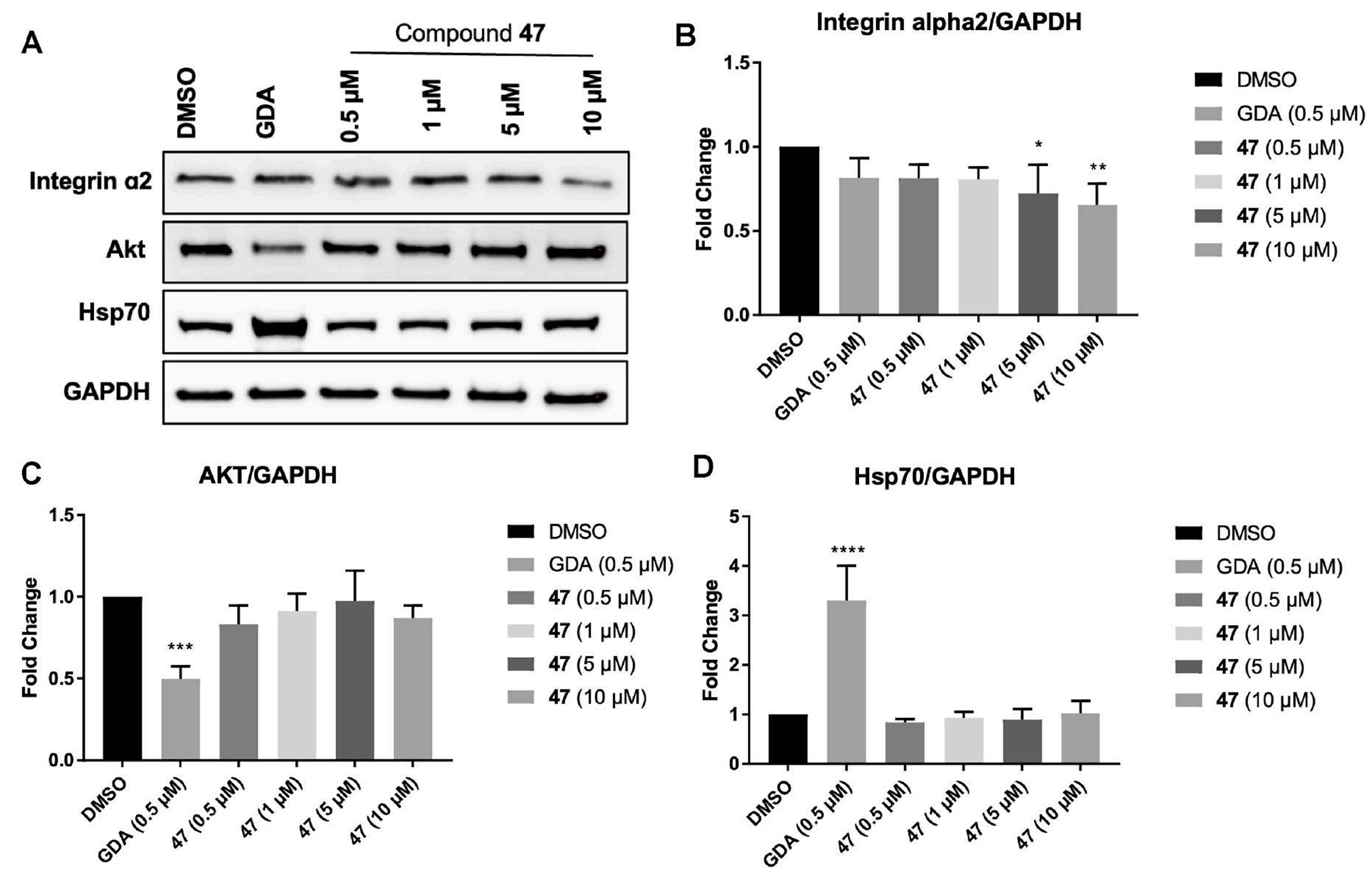
(A) Western blot analysis of integrin α2, Akt, Hsp70 and GAPDH in MDA-MB-231 cells treated with compound **47** at indicated concentrations, geldanamycin (GDA, 0.5 μM), or vehicle control (DMSO, 0.1 % final concentration). Ratio of integrin α2 (B), Akt (B), and Hsp70 (C) normalized to GAPDH for each concentration of compound **47**.

Integrin α2 is a cell surface receptor that mediates cancer cell adhesion, migration, and signaling, and Grp94 is required for the proper folding and maturation of integrin α2. Inhibition of Grp94 promotes the degradation of integrin α2, thereby suppressing tumor cell invasion and metastasis. As shown in Fig. 3, treatment with compound **47** reduced the levels of Integrin α2 at 5 μM in the breast cancer cell line MDA-MB-231. The observed decrease in Integrin α2 is likely due to impaired maturation. In contrast, compound **47** did not affect Akt, a cytosolic Hsp90 client protein. This is consistent with the observation that compound **47** is selective for Grp94 and, in contrast with the pan-inhibitor geldanamycin (GDA), does not induce Hsp70 expression (see Fig. 3a).

**Fig. 4.**
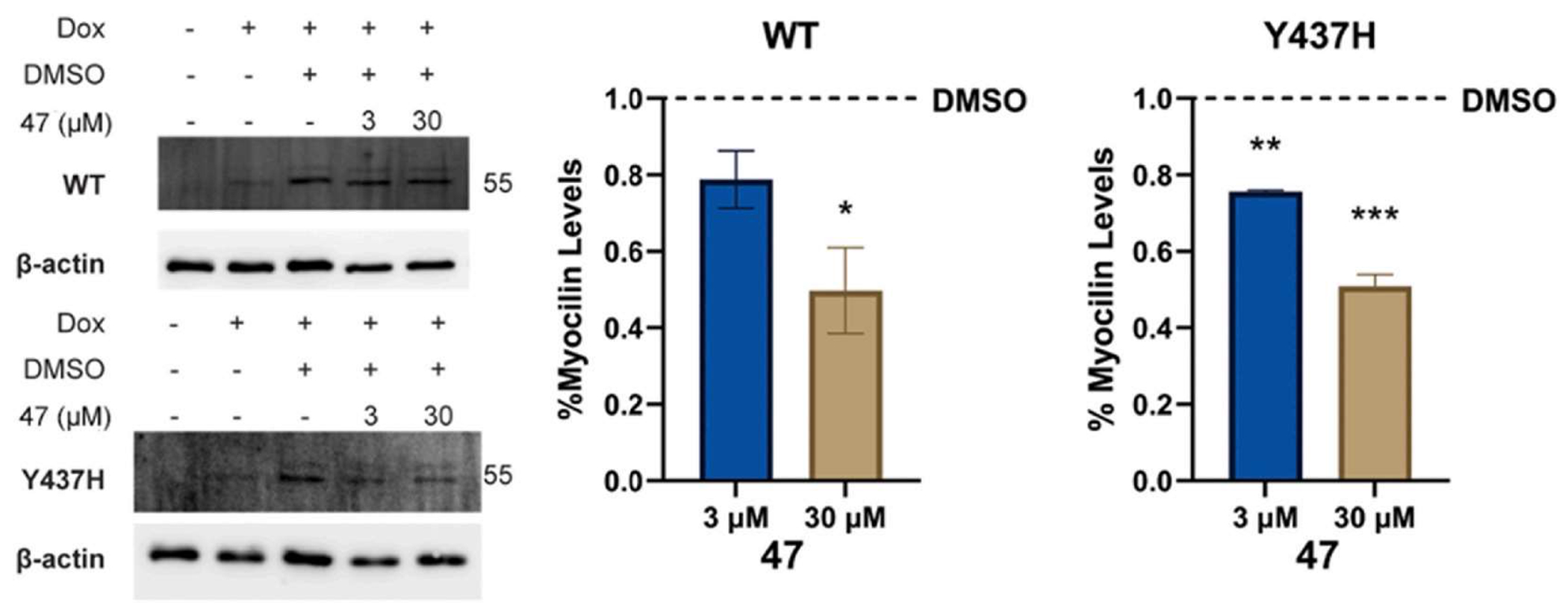
Cellular assay for mutant myocilin. An immortalized, doxycycline-inducible (dox) human trabecular meshwork cell line was used to test the effect of compound **47** on mutant myocilin localization. Both compounds decreased intracellular accumulation of myocilin compared to vehicle control (DMSO). Blots are representative of three independent experiments; statistics were conducted on quantification of all replicates combined.

Grp94 functions as an important chaperone that stabilizes mutant myocilin by promoting its aggregation. Inhibition or knockdown of Grp94 enhances autophagic removal of mutant myocilin and reduces intracellular levels [16,17]. Therefore, compound **47** was also tested in a cellular model of myocilin-associated glaucoma. An immortalized, doxycycline-inducible (dox) human trabecular meshwork cellular model stably transduced with either wild-type or mutant Y437H myocilin was treated with 3 μM or 30 μM of compound **47** for 24 h (Fig. 4). A statistically significant reduction in Y437H-mutant myocilin levels was observed upon treatment with **47** at 3 μM for 24 h and reduced the levels of intracellular myocilin by 50 % or more upon treatment at 30 μM. Interestingly, at the higher dose, there was also a statistically significant decrease in wild-type myocilin expression perhaps suggestive of a misfolded species present due to the relatively high levels of expression.

## 3. Conclusion

A nitrogen scan was performed on the first generation of Grp94-selective inhibitor, **BnIm**, which yielded a triazole ring system that exhibited enhanced binding affinity. Subsequent structure-activity relationship studies focused on the aryl side chain, wherein incorporation of a 2-propyl hydrocarbon chain produced compound **47** that manifests in 76 nM affinity for Grp94 and 121-fold selectivity over Hsp90α. Molecular docking studies revealed an essential hydrogen bond interaction between Asp162 and the triazole nitrogen that stabilizes the ethylene linker and thereby minimizes rotation and projects the aryl side chain into the site 1 pocket. Evaluation of compound **47** on cells via Western blot analysis demonstrated the degradation of integrin α2 at 5 μM, indicating that Grp94 inhibition prevents cell migration. Compound **47** also reduced the intracellular accumulation of mutant myocilin in a trabecular meshwork cell line, highlighting its potential use to treat glaucoma.

## 4. Materials and methods

### 4.1. Chemistry

All reagents and solvents were obtained from commercial sources and used as received unless otherwise noted. Reactions were conducted in flame- or oven-dried glassware under an argon atmosphere. Thin-layer chromatography (TLC) was performed with TLC Silica gel 60F254 plates purchased from Millipore Sigma and visualized using molybdenum stain or UV light at 254 nm. Flash column chromatography was performed using silica gel (40–63 μm particle size).^1^H and ^13^C NMR spectra were recorded on Bruker 400 MHz or 500 MHz instruments, with chemical shifts (*δ*, ppm) referenced to the residual solvent peaks and coupling constants (J) reported in Hertz (Hz). High-resolution mass spectral data were obtained using a time-of-flight mass spectrometer with electrospray ionization. HPLC purity analyses were determined on an Agilent 1260 Infinity II HPLC system equipped with an autosampler, using an Agilent Eclipse Plus C18 column (3.5 μm, 4.6 × 100 mm) and an 80:20 acetonitrile/water mobile phase (4.0 mL/min, 20 min). UV detection was monitored at 214 nm.

#### 4.1.1. Synthesis of methyl 4,6-bis((tert-butyldimethylsilyl)oxy)-3-chloro-2-methylbenzoate (7)

Orcinol (**4**) (3 g, 1 eq, 24.2 mmol) was dissolved in acetonitrile (40.0 mL) followed by the addition of DBU (11.0 g, 10.9 mL, 3 Eq, 72.5 mmol). The reaction flask was vacuum, and CO_2_ was then charged. The reaction mixture was stirred at room temperature for 12 h. 6 M HCl aq. (ca. 100 mL) was used to quench the reaction mixture. The resulting mixture was subsequently extracted with ethyl acetate 3 times. The organic layer was then combined, dried over Na_2_SO_4_, and was concentrated under reduced pressure. The crude was used without further purification.

The crude product was dissolved in acetone (70 mL), followed by the addition of iodomethane (2.11 mL, 1.2 eq, 22.1 mmol) and sodium bicarbonate (1.86 g, 1.2 eq, 22.1 mmol). The reaction mixture was stirred at room temperature for 12 h, after which water (50 mL) and ethyl acetate (50 mL) were added. The organic phase was separated and washed with water (3 × 50 mL), dried over anhydrous Na_2_SO_4_, and concentrated under reduced pressure to afford methyl 2,4-dihydroxy-6-methylbenzoate as a white amorphous solid, used without further purification.

To a solution of methyl 2,4-dihydroxy-6-methylbenzoate in DMF (30 mL) was added TBSCl (7 g, 3 eq, 50 mmol) and imidazole (2 g, 2eq, 30 mmol). The mixture was stirred at room temperature for 12 h, after which water (150 mL) and ethyl acetate (150 mL) were added. The organic layer was washed with water (5 × 50 mL), brine (1 × 20 mL), dried over anhydrous Na_2_SO_4_, and concentrated to provide methyl 2,4-bis((*tert*-butyldimethylsilyl)oxy)-6-methylbenzoate (**6**) as a transparent oil.

Without purification, the crude was dissolved in THF (50 mL) and cooled to −40 °C followed by the addition of SO_2_Cl_2_ (0.876 mL, 1 eq, 10.8 mmol) dropwise over 15 min. The reaction was stirred at −40 °C for another 30 min and quenched with saturated NH_4_Cl and extracted with ethyl acetate (3 × 50 mL). The organic layers were combined, dried, and concentrated. The reaction mixture was purified via flash chromatography (SiO_2_, 5 % EtOAc in hexanes) to provide methyl 4,6-bis((tertbutyldimethylsilyl)oxy)-3-chloro-2-methylbenzoate (**7**) as a transparent oil (3.8 g, 34 %).

#### 4.1.2. General synthesis of methyl 2-(but-3-yn-1-yl)-4,6-bis((tertbutyldimethylsilyl)oxy)-3-chlorobenzoate (8) and methyl 2-(2-bromoethyl)-4,6-bis((tert-butyldimethylsilyl)oxy)-3-chlorobenzoate (11)

To a solution of **7** (200 mg, 1 eq, 0.45 mmol) dissolved in anhydrous THF (2 mL) at −78 °C was added dropwise of a 1.0 M solution of lithium diisopropylamide LDA (0.539 mL, 1.2 eq, 0.539 mmol). The mixture was stirred for 10 min under argon before the addition of propargyl bromide or dibromomethane (1.2 eq, 0.539 mmol) at once. The reaction was then left running at −78 °C for 10 min at which point cold saturated aqueous NH_4_Cl (10 mL) was added to quench the reaction. The mixture was extracted with ethyl acetate (10 mL) and the aqueous layer was washed with EtOAc (3 × 10 mL). The organic layer was combined and dried with anhydrous Na_2_SO_4_, and concentrated. The mixture was purified via flash chromatography (SiO_2_, 10 % EtOAc in Hexanes) to afford as an amorphous solid (168 mg, 60–77 %).

#### 4.1.3. General procedure for ruthenium catalyzed azide-alkyne cycloaddition (RuAAC) reactions

Azides (1 Eq, 0.1 mmol) and terminal alkynes (1 eq, 0.10 mmol) were mixed in 1.0 mL of 1,4-dioxane followed by the addition of Cp*RuCl (PPh_3_)_2_ (0.008 eq, 0.008 mmol). The vial was charged with nitrogen, sealed, and heated in an oil bath at 60 °C for 12 h. After the reaction finished, water (10 mL) and EtOAc (10 mL) were added. The organic layer was extracted with water (3 × 10 mL), brine (1 × 10 mL), and dried over anhydrous Na_2_SO_4_. The residue was purified via flash chromatography (SiO_2_, 40 % EtOAc in Hexanes) to provide corresponding triazoles. The triazole product (1 eq, 74 μmol) was subsequently dissolved in THF followed by the addition of TBAF (1.0 M, 1.2 Eq, 89 μmol). The reaction was stirred at room temperature for 2 h at which point the solvent was removed under reduced pressure and the mixture was purified via flash chromatography (SiO_2_, 50 % EtOAc in Hexanes) to provide the corresponding final product.

#### 4.1.4. General procedure for copper catalyzed azide-alkyne cycloaddition (CuAAC) reactions

**15** (1 Eq, 0.6 mmol) and **13** (1.5 eq, 0.9 mmol) were dissolved in 1.0 mL of THF followed by the addition of CuSO_4_*5H_2_O (0.3 eq, 0.18 mmol) and sodium ascorbate (0.3 eq, 0.18 mmol). The vial was charged with nitrogen, sealed, and heated at 80 “C for 12 h, at which point water (10 mL) and EtOAc (10 mL) were added. The organic layer was washed with water (3 × 10 mL), brine (1 × 10 mL), and dried over anhydrous Na_2_SO_4_. The residue was purified via flash chromatography (SiO_2_, 40 % EtOAc in Hexanes). The triazole product (1 eq, 84 μmol) was subsequently dissolved in THF followed by the addition of TBAF (1.0 M, 1.2 Eq, 89 μmol). The reaction was stirred at room temperature for 2 h at which point the solvent was removed under reduced pressure and the mixture was purified via flash chromatography (SiO_2_, 50 % EtOAc in Hexanes) to provide corresponding final product.

#### 4.1.5. Synthesis of methyl 4,6-bis(benzyloxy)-2-(2-bromoethyl)-3-chlorobenzoate (17)

To a solution of **11** in 1.0 mL of THF was added to TBAF (1.0 M, 1.2 Eq, 89 μmol), and the reaction was stirred at room temperature for 2 h. The mixture was extracted with ethyl acetate and water (3 × 5 mL). The organic layer was combined, dried over anhydrous Na_2_SO_4_, and concentrated under reduced pressure. The reaction mixture was then dissolved in DMF (3 mL), followed by the addition of K_2_CO_3_ (2.5 Eq, 1.70 mmol) and benzyl bromide (2.5 Eq, 6.78 mmol). The reaction was stirred at room temperature for 16 h, after which water (10 mL) and EtOAc (10 mL) were added. The organic layer was washed with water (3 × 10 mL), brine (1 × 10 mL), and dried over anhydrous Na_2_SO_4_. The residue was purified via flash chromatography (SiO_2_, 10 % EtOAc in Hexanes) to afford white solid in quantitative yield.

#### 4.1.6. Synthesis of methyl 2-(2-(5-benzyl-lH-tetrazol-l-yl)ethyl)-3-chloro-4,6-dihydroxybenzoate (20)

**17** (1 mmol), **19** (1 mmol), and K_2_CO_3_ (2 mmol) were dissolved in DMF (5 mL). The mixture was stirred under argon atmosphere at 60 °C for 16 h. The reaction mixture was extracted with EtOAc (3 × 10 mL) and water (3 × 10 mL). The combined organic layers were combined and dried over Na_2_SO_4_ and concentrated under reduced pressure. The crude was purified via flash chromatography (SiO_2_, 50 % EtOAc in Hexanes). The product was then dissolved in ethyl acetate (1 mL), evacuated, and purged with argon for 3 times followed by the addition of 10 % Pd/C. The reaction was then evacuated again and charged with two hydrogen balloons. The reaction was stirred under hydrogen atmosphere for 16 h before filtered through a pad of celite and concentrated to afford white amorphous solid (5 mg, 18 %).

#### 4.1.7. General procedure for the synthesis of 22a-c and 24a-c

To the solution of 2-hydroxybenzaldehyde or 2-mercaptobenzoate (200 mg, 1 Eq) (200 mg, 1 Eq) in DMF (20 mL) was added to K_2_CO_3_ (296 mg, 1.5 Eq) and corresponding alkyl iodides (0.168 mL, 5 Eq). The mixture was stirred at room temperature and under nitrogen atmosphere for 16 h after which ice (10 mL) and EtOAc (10 mL) were added. After ice melted, the aqueous was extracted with EtOAc (3 × 10 mL). The organic layer was combined, dried over anhydrous Na_2_SO_4_, and concentrated to provide **22a-c** or **24a-c** as a clear or yellow oil.

#### 4.1.8. General procedure A for the synthesis of benzyl alcohols 25a-c

Commercially available or pre-synthesized aldehydes **22a-c** (1.0 eq, 2.43 mmol) were dissolved in absolute EtOH (5 mL) and NaBH_4_ (1.2 eq, 2.02 mmol) was added portion-wise. The mixture was stirred at room temperature for 3 h. After the completion of the reaction, water was added dropwise to quench the reaction. The organic layer was then extracted and washed with water, brine, dried and concentrated under reduced pressure. The mixture was purified via flash chromatography (SiO_2_, 20 % EtOAc in Hexanes).

#### 4.1.9. General procedure B for the synthesis of benzyl alcohols 25e-f

Commercially available or pre-synthesized esters **24a-c** (1.0 eq, 2.00 mmol) were dissolved in Et_2_O (5 mL) at 0 °C followed by dropwise addition of LiAlH_4_ (1.5 eq, 2.52 mmol, 2 M in THF). Ice bath was removed, and the mixture was stirred at room temperature for 3 h. After the completion of the reaction, water was added dropwise to quench the reaction. EtOAc (5 mL) and then 1 M HCl (2 mL) was added dropwise to the reaction mixture at 0 °C. The organic layer was then extracted and washed with water, brine, dried and concentrated under reduced pressure followed by flash chromatography (SiO_2_, 15 % EtOAc in Hexanes).

#### 4.1.10. General procedure C for the synthesis of benzyl alcohols 28a-e

To a solution of 2-methylbenzyl alcohol (500 mg, 1 Eq, 8.2 mmol) in Et_2_O was added n-butyllithium (2.5 M in hexane, 6.5 mL, 2 Eq, 16 mmol) at 0 °C. The resulting cloudy mixture was stirred and heated to reflux for 4 h. After cooling to room temperature, corresponding alkyl iodide (0.40 mL, 1.2 Eq, 8.2 mmol) was added dropwise over a course of 15 min and the reaction was stirred for an additional 1 h before slowly quenched with water. The mixture was extracted with EtOAc, and the combined organic layers were dried, concentrated, and purified via flash chromatography (20 % EtOAc/hexanes) to afford the corresponding alcohols.

#### 4.1.11. General procedure for the synthesis of benzyl bromides 26a-f and 29a-e

To a solution of benzyl alcohols **25a-f** or **28a-c** (1 eq, 2.31 mmol) in anhydrous DCM (8 mL) was added dropwise phosphorus tribromide PBr_3_ (1.3 eq, 3.00 mmol) at room temperature. The mixture was stirred at room temperature for another 2 h. Water (15 mL) was added to quench the reaction. The aqueous layer was washed with DCM (3 × 10 mL). The combined organic layers were washed with brine, dried over anhydrous Na_2_SO_4_, and concentrated to afford benzyl bromides. The products were used without further purification.

#### 4.1.12. General procedure for the synthesis of terminal alkynes 30a-k

*i*-PrMgCl (2.0 M in THF, 4.40 mL, 4 eq, 8,81 mmol) was added dropwise to a solution of ethynyltrimethylsilane (0.94 mL, 4 eq, 8.81 mmol) in THF (2 mL) at 0 °C. The reaction mixture was stirred for 30 min at 0 °C and then for an additional 30 min at room temperature. CuBr (189 mg, 1.32 mmol, 0.6 equiv) was added in one portion, and the mixture was stirred for 30 min at room temperature. Benzyl bromide (500 mg, 2.20 mmol, 1.0 equiv) was then added, and the reaction was heated to reflux for 5 h. After cooling to room temperature, saturated aqueous NH_4_Cl (10 mL) was poured into the reaction mixture and extracted with EtOAc (3 × 20 mL). The combined organic layers were washed with water (10 mL), dried over Na_2_SO_4_, and concentrated under reduced pressure. The crude was purified by flash chromatography (100 % hexanes) to afford the product.

The obtained product (1 eq, 1.35 mmol) was dissolved in methanol (5 mL) and K_2_CO_3_ (0.5 eq, 0.674 mmol) was added. The mixture was stirred at room temperature for 2 h, after which water (10 mL) and EtOAc (10 mL) were added. The organic layer was separated, washed with water (3 × 10 mL) and brine (10 mL), dried over anhydrous Na_2_SO_4_, and concentrated to give compounds **32a-k**.

### 4.2. Fluorescence polarization (FP) assay

The Fluorescence Polarization Assay (FP) was performed in 96-well flat-bottom plates (Santa Cruz Biotechnology) with a final volume of 100 μL per well. The assay buffer consisted of 20 mM HEPES, 50 mM KCl, 10.5 mM MgCl_2_, 20 mM Na_2_MoO_4_, 0.01 % NP-40 detergent (NP-40), and pH 7.3 with fresh 2 mM dithiothreitol (DTT) and 0.1 mg/mL bovine γ-globulin (BGG). Each well contained 10 nM of Hsp90α or Grp94 (Enzo Biochem Inc.) and 6 nM of FITC-GDA (fluorescent tracer, stock in DMSO). Compounds were tested in triplicate at final concentrations of 100 μM, 10 μM, 1 μM, 0.1 μM and 0.01 μM with each final concentration containing 1 % DMSO. Each plate included control wells containing: (i) buffer only (background polarization), (ii) protein plus tracer (maximum polarization control), and (iii) tracer only (minimum polarization control). Plates were incubated on a rocker at 4 °C for 24 h. Fluorescence polarization (mP units) was measured at 37 °C using excitation and emission wavelengths of 485 and 528 nm, respectively. Apparent Kd values were determined via calculations from the polarization data obtained at which the tracer was displaced by 50 % by the compounds of interest.

### 4.3. Cell culture and immunoblot

MDA-MB-231 cells were grown in a water jacketed incubator at 37 °C with 5 % CO_2_. Cells were cultured in DMEM (Corning) media supplemented with 10 % heat-inactivated fetal bovine serum (FBS, Gibco, 10438-026) and 1 % Penicillin-Streptomycin (VWR, K952-100 mL). Cells were seeded in clear, flat bottom 6-well plates with each well containing 500,000 cells and grown to 80 % confluency. The medium was replaced with fresh DMEM that contains compounds of interest at the indicated concentrations or vehicle control, and cells were incubated for 24 h. Following treatment, MDA-MB-231 cells were then lysed in RIPA buffer supplemented with protease and phosphatase inhibitors. Protein concentration in lysates were determined by BCA assay (Thermo). Equal amounts of protein were separated by SDS-PAGE on 10 % polyacrylamide gels and transfected onto nitrocellulose membranes. Transfer efficiency and total protein loading were confirmed by Ponceau staining. Membranes were blocked with 6 % nonfat dry milk and probed with primary antibodies against GAPDH (Cell Signaling, 14C10), Hsp70 (Cell Signaling, C92F3A), Akt, and Integrin α2 (Cell Signaling, D8V7H) each diluted 1:1000. Secondary antibodies were also used at 1:1000 (Southern Biotech), immunoblots were developed using ECL substrate (Amersham, 45-000-999) and visualized on a ChemiDoc Imaging System (BioRad).

Immortalized human trabecular meshwork (TM-1) cells stably transduced with WT or Y437H myocilin (50 MOI) were grown in low-glucose (1 g/L) Dulbecco’s modified Eagle medium with glutamine (DMEM, Gibco) supplemented with 10 % FBS (Hyclone), 1–2 % PS (Gibco) at 37 °C, and 200 μg/mL hygromycin (Corning) [21], TM-1 cells were seeded in 6 well plates at equivalent cell counts/replicate. The following day, myocilin expression was induced by treating the cells with 2 μg/mL doxycycline (dox, Thermo-Scientific) in serum-free media. After 48 h, media was replaced with serum-free media containing 2 μg/mL dox to induce myocilin expression and 47 at the designated concentration for an additional 24 h.

Cells were lysed using lysis buffer (100 mM Tris-HCl pH 7.4, 3 mM EGTA, 5 mM MgCl_2_, 0.5 % Triton X 100) supplemented with 0.6 mM phenylmethylsulfonyl fluoride, and a 1:100 v/v protease inhibitor cocktail containing complete EDTA-free protease inhibitor (Roche) and 1 % (v/v) phosphatase inhibitor cocktails II and III (Sigma-Aldrich). Cells lysed by rocking in lysis buffer for ~2 h at 4 °C. Detergent-soluble and detergent insoluble fractions were separated by centrifugation. Protein concentration of the detergent-soluble fractions was determined by BCA assay (Pierce).

The detergent-soluble samples were prepared in equal protein concentration in 5X Laemmli buffer containing 5–10 % (v/v) β-mercaptoethanol. The samples were heated at 95 °C for 5 min and loaded on 12 % Tris-glycine gels. The gels were then transferred to methanol-activated PVDF membranes using the *Trans*-blot Turbo transfer system (Bio-Rad). The membranes were blocked with 5 % milk in PBS+0.5 % Tween, washed three times with PBS+0.1 % Tween, and then incubated with 1:1000 dilution of primary antibodies (Mouse anti-myocilin, MAM3446, R&D Systems and rabbit anti-beta actin, 4970, Cell Signaling). Following incubation, the membranes were again washed three times with PBS+0.1 % Tween, and then incubated with 1:3000 dilution of secondary antibodies (anti-mouse Starbright blue 520 and anti-rabbit Starbright blue 700, Bio-Rad). After a final three washes with PBS, the blots were visualized using ChemiDoc MP Image System (BioRad). Quantification of protein bands on Western blots was performed using ImageLab (Bio-Rad). Measurements of band density were normalized to actin loading control within each experiment, and the statistical significance was determined with one-way ANOVA with GraphPad Prism.

### 4.4. Computational molecular modeling

Computational studies were performed using the Maestro molecular modeling suite (Schrodinger, LLC, New York, NY, USA). Protein and ligand preparation, receptor grid generation, and molecular docking were conducted using the integrated modules LigPrep, Epik, and Glide, respectively. Protein structures were prepared using the Protein Preparation Wizard in Maestro, which included the addition of hydrogen atoms, assignment of bond orders, optimization of hydrogen-bonding networks, and restrained minimization using the OPLS4 force field. Ligands were generated and energy minimized with LigPrep, accounting for all possible tautomers and protonation states predicted by Epik at physiological pH (7.0 ± 2.0). Receptor grids were generated using Glide centered on the co-crystallized ligand or the known active site residues. Docking studies were performed using Glide Standard Precision (SP), and the top-ranked poses were evaluated based on docking scores and key protein–ligand interactions. Visualization was conducted on PyMOL.

## Supplementary Material

supporting data

## Figures and Tables

**Table 1:** Apparent binding affinity for Grp94 and selectivity over Hsp90α. K_d_ values reported as the mean of triplicates ± SD. NS = non-selective.

No.	Apparent K_d_ Grp94 (μM)	Apparent K_d_ Hsp90α (μM)	Fold selective for Grp94
**BnIm (1)**	1.14 ± 0.01	13.1 ± 1.10	12
**10**	0.57 ± 0.02	0.62 ± 0.04	n.s.
**14**	0.69 ± 0.12	5.24 ± 0.24	8
**16**	7.22 ± 0.41	6.79 ± 0.10	n.s.
**20**	0.56 ± 1.61	1.63 ± 0.88	3

**Table 2:** Apparent binding affinity of compound **14** analogs for Grp94 and selectivity over Hsp90α. K_d_ values reported as the mean of triplicates ± SD. n.s. = non-selective.

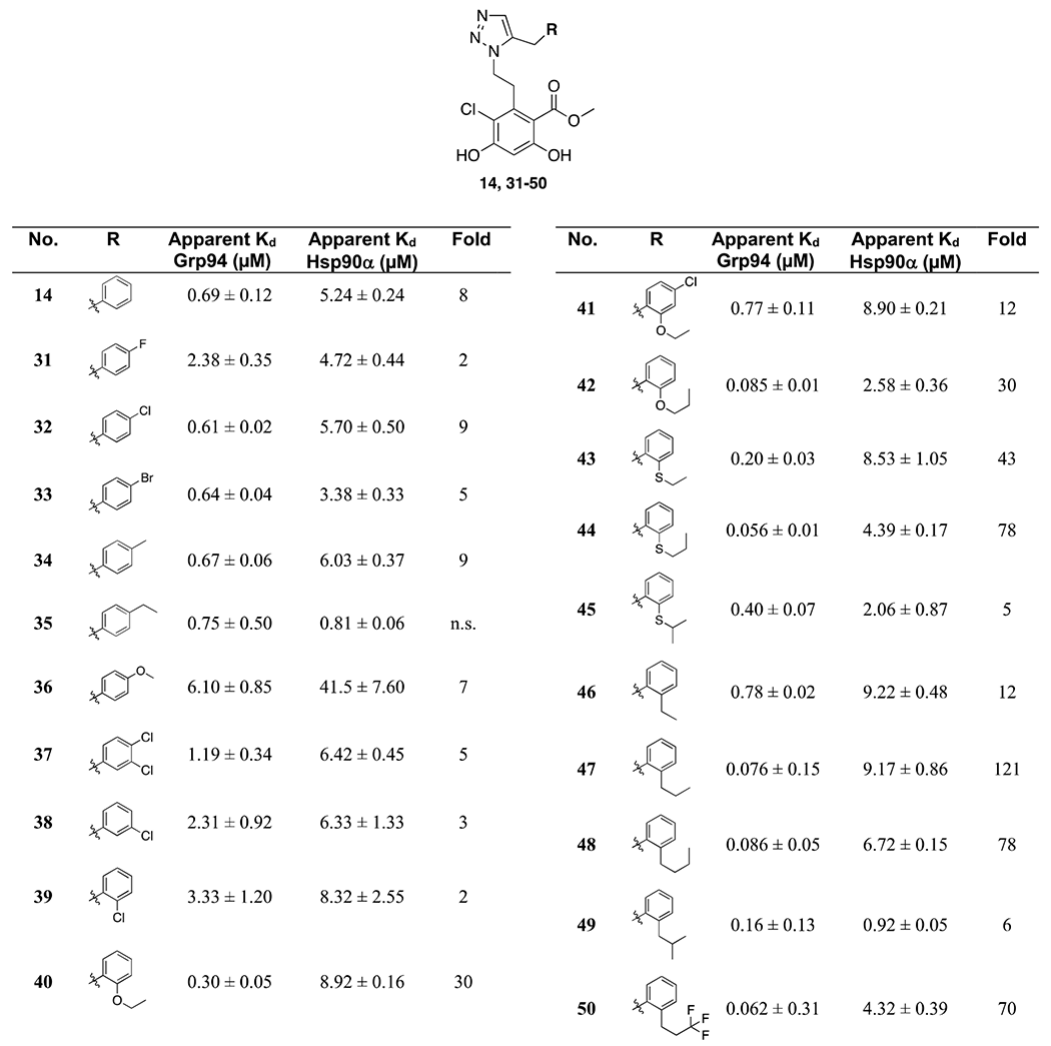

## Data Availability

Data will be made available on request.

## Appendix A. Supplementary data

Supplementary data to this article can be found online at https://doi.org/10.1016/j.ejmech.2025.118394.
