# Supplementary material for: Design, synthesis, and biological properties exhibited by 1,2,3-triazole based Grp94-selective inhibitors
Source: Eur J Med Chem. Author manuscript; Available in PMC 2026 May 26. (PMC13205134; doi:10.1016/j.ejmech.2025.118394)
Supplement: supporting data [file NIHMS2174109-supplement-supporting_data.docx]

**Supporting Information for**

**Design, Synthesis, and Biological Properties Exhibited by 1,2,3-Triazole Based Grp94-selective Inhibitors**

**Hao Xu^[1]^, Dustin J. E. Huard^[2]^, Elijah Dunn^[2]^, Lucas A. Chalfoun^[1]^, Felix Adulley^[1]^, Raquel L. Lieberman^[2]^, and Brian S. J. Blagg^[1]*^**

^1^Department of Chemistry and Biochemistry, Warren Center for Drug Discovery, The University of Notre Dame, 305 McCourtney Hall, Notre Dame, IN, 46556, USA, E-mail: [bblagg@nd.edu](mailto:bblagg@nd.edu)

^2^School of Chemistry & Biochemistry, Georgia Institute of Technology, Atlanta, GA, 30332, USA

**Table of Contents**

1. Characterization of Compounds S3 – S17
2. References S17
3. Fluorescence Polarization (FP) Assay S18
4. Spectra Data S18 – S42

**Experimental**

All reagents and solvents were obtained from commercial sources and used as received unless otherwise noted. Reactions were conducted in flame- or oven-dried glassware under an argon atmosphere. Thin-layer chromatography (TLC) was performed with TLC Silica gel 60F254 plates purchased from Millipore Sigma and visualized using molybdenum stain or UV light at 254 nm. Flash column chromatography was performed using silica gel (40–63 μm particle size). 1H- and 13C-NMR spectra were recorded on Bruker 400 MHz or 500 MHz instruments, with chemical shifts (δ, ppm) referenced to the residual solvent peaks and coupling constants (J) reported in Hertz (Hz). High-resolution mass spectral data were obtained using a time-of-flight mass spectrometer with electrospray ionization. HPLC purity analyses were determined on an Agilent 1260 Infinity II HPLC system equipped with an autosampler, using an Agilent Eclipse Plus C18 column (3.5 μm, 4.6 × 100 mm) and an 80:20 acetonitrile/water mobile phase (4.0 mL/min, 20 min). UV detection was monitored at 214 nm.

**methyl 4,6-bis((tert-butyldimethylsilyl)oxy)-3-chloro-2-methylbenzoate (7)**

Clear oil (34%). ^1^H NMR (400 MHz, CDCl_3_) δ 6.31 (s, 1H), 3.87 (s, 3H), 2.30 (s, 3H), 1.03 (s, 9H), 0.96 (s, 9H), 0.20 (d, *J* = 5.8 Hz, 12H). Spectra data were in agreement with literature values.^1^

**methyl 2-(but-3-yn-1-yl)-4,6-bis((tert-butyldimethylsilyl)oxy)-3-chlorobenzoate (8)**

White amorphous solid (77%). ^1^H NMR (400 MHz, CDCl_3_) δ 6.31 (s, 1H), 3.87 (s, 3H), 2.96 – 2.88 (m, 2H), 2.45 (m, 2H), 1.98 (t, *J* = 2.7 Hz, 1H), 1.03 (s, 9H), 0.95 (s, 9H), 0.21 (d, *J* = 6.9 Hz, 12H). ﻿^13^C NMR (126 MHz, CDCl_3_) δ 168.13, 153.25, 151.68, 137.73, 121.39, 118.88, 110.00, 83.74, 68.79, 52.44, 31.69, 25.87, 25.84, 25.69, 25.67, 18.67, 18.56.

**methyl 2-(2-(1-benzyl-1H-1,2,3-triazol-5-yl)ethyl)-3-chloro-4,6-dihydroxybenzoate (10)**

General procedure of RuAAC reactions. White amorphous solid (10 mg, 78%). ^1^H NMR (400 MHz, MeOD) δ 7.65 (s, 1H), 7.42 – 7.29 (m, 3H), 7.22 – 7.15 (m, 2H), 6.44 (s, 1H), 5.60 (s, 2H), 4.65 (s, 1H), 3.78 (s, 3H), 3.28 – 3.20 (m, 2H), 2.92 (dd, *J* = 9.4, 6.8 Hz, 2H). ^13^C NMR (101 MHz, CDCl_3_) δ 171.41, 162.70, 160.73, 146.57, 144.62, 133.02, 131.25, 128.91, 127.63, 125.19, 115.00, 107.37, 104.82, 55.74, 51.66, 32.16, 30.15. Purity: 98% HRMS (ESI) m/z [M+1] calculated for C_19_H_18_ClN_3_O_4_, 388.1059, found 388.1059.

******

**methyl 2-(2-bromoethyl)-4,6-bis((tert-butyldimethylsilyl)oxy)-3-chlorobenzoate (11)**

Brown oil (60%). ^1^H NMR (400 MHz, CDCl_3_) δ 6.34 (s, 1H), 3.88 (s, 3H), 3.55 – 3.46 (m, 2H), 3.24 – 3.15 (m, 2H)), 1.02 (d, *J* = 3.2 Hz, 9H), 1.00 – 0.91 (m, 9H), 0.26 (d, *J* = 6.8 Hz, 12H).

**methyl 2-(2-azidoethyl)-4,6-bis((tert-butyldimethylsilyl)oxy)-3-chlorobenzoate (12)**

Quantitative yield. ^1^H NMR (400 MHz, CDCl_3_) δ 6.28 (s, 1H), 3.85 (s, 3H), 3.48 – 3.40 (m, 2H), 3.02 – 2.93 (m, 2H), 1.02 (s, 9H), 0.95 (s, 9H), 0.21 (dd, *J* = 6.6, 3.0 Hz, 12H). ﻿^13^C NMR (126 MHz, CDCl3) δ 168.04, 153.36, 151.93, 135.10, 121.77, 119.11, 110.33, 52.50, 50.36, 31.82, 25.96, 25.94, 25.91, 25.88, 25.86, 25.82, 25.79, 25.77, 25.75, 25.72, 25.69, 25.66.

**methyl 2-(2-(5-benzyl-1H-1,2,3-triazol-1-yl)ethyl)-3-chloro-4,6-dihydroxybenzoate (14)**

General procedure of RuAAC reactions. White amorphous solid (12 mg, 69%). ^1^H NMR (400 MHz, CDCl_3_) δ 11.42 (s, 1H), 7.44 (s, 1H), 7.31 (q, *J* = 6.8 Hz, 3H), 7.12 – 7.05 (m, 2H), 6.65 (s, 1H), 4.48 – 4.39 (m, 2H), 4.00 (s, 3H), 3.93 (s, 2H), 3.67 (dd, *J* = 8.8, 6.7 Hz, 2H). ^13^C NMR (101 MHz, CDCl_3_) δ 170.34, 163.30, 156.50, 137.72, 135.91, 135.79, 133.30, 129.03, 128.29, 127.38, 114.42, 107.13, 103.80, 53.00, 46.76, 33.56, 29.06. Purity: 97% HRMS (ESI) m/z [M+1] calculated for C_19_H_18_ClN_3_O_4_, 388.1059, found 388.1069.

**methyl 2-(2-(4-benzyl-1H-1,2,3-triazol-1-yl)ethyl)-3-chloro-4,6-dihydroxybenzoate (16)**

White amorphous solid (52%). ^1^H NMR (400 MHz, CDCl_3_) δ 11.38 (s, 1H), 7.36 – 7.28 (m, 2H), 7.24 (s, 3H), 7.05 (s, 1H), 6.63 (s, 1H), 4.54 (t, *J* = 7.7 Hz, 2H), 4.10 (s, 2H), 3.94 (s, 3H), 3.66 (t, *J* = 7.6 Hz, 2H). ^13^C NMR (101 MHz, CDCl_3_) δ 170.27, 163.27, 156.67, 147.65, 138.71, 137.55, 128.74, 128.72, 126.67, 121.74, 114.53, 106.90, 103.82, 52.90, 49.23, 33.84, 32.10. Purity: 97% HRMS (ESI) m/z [M+1] calculated for C_19_H_18_ClN_3_O_4_, 388.1059, found 388.1053.

******

**methyl 4,6-bis(benzyloxy)-2-(2-bromoethyl)-3-chlorobenzoate (17)**

White solid. Quantitative yield. ^1^H NMR (400 MHz, CDCl_3_) δ 7.41 – 7.29 (m, 10H), 6.49 (s, 1H), 5.09 (s, 2H), 5.02 (s, *J* = 2.9 Hz, 2H), 3.89 (s, *J* = 9.9 Hz, 3H), 3.54 (dd, *J* = 9.6, 7.1 Hz, 2H), 3.25 (dd, *J* = 9.7, 7.1 Hz, 2H).

******

**5-benzyl-1*H*-tetrazole (19)**

Synthesis was previously reported. Spectra data were in agreement with literature values.^2^

***^^***

**methyl 2-(2-(5-benzyl-1*H*-tetrazol-1-yl)ethyl)-3-chloro-4,6-dihydroxybenzoate (20)**

White amorphous solid (18%). ^1^H NMR (500 MHz, CDCl_3_) δ 11.41 (s, 1H), 7.34 – 7.30 (m, 4H), 7.24 (tq, *J* = 6.6, 2.9 Hz, 1H), 6.96 (s, 1H), 6.57 (s, 1H), 4.85 – 4.78 (m, 2H), 4.25 (s, 2H), 3.91 (s, 3H), 3.76 – 3.70 (m, 2H). ^13^C NMR (126 MHz, CDCl_3_) δ 170.57, 165.91, 163.54, 156.76, 137.27, 136.83, 129.07, 128.92, 127.16, 114.82, 107.22, 104.06, 53.02, 51.72, 33.30, 32.10. Purity: 95% HRMS (ESI) m/z [M+23] calculated for C_23_H_26_ClN_3_NaO_4_, 411.0831, found 411.0824.

**1-(bromomethyl)-2-ethoxybenzene (26a)**

Brown oil (70%). ^1^H NMR (400 MHz, CDCl_3_) δ 7.36 (dd, *J* = 7.4, 1.7 Hz, 1H), 7.33 – 7.26 (m, 1H), 6.97 – 6.85 (m, 2H), 4.62 (s, 2H), 4.14 (q, *J* = 7.0 Hz, 2H), 1.50 (td, *J* = 7.0, 0.9 Hz, 3H).

**1-(bromomethyl)-2-propoxybenzene (26b)**

Brown oil (70%). ^1^H NMR (400 MHz, CDCl_3_) δ 7.33 (dd, *J* = 7.5, 1.7 Hz, 1H), 7.27 (td, *J* = 7.9, 1.8 Hz, 1H), 6.94 – 6.80 (m, 2H), 4.59 (s, 2H), 4.00 (t, *J* = 6.4 Hz, 2H), 1.87 (dtd, *J* = 13.6, 7.3, 6.3 Hz, 2H), 1.10 (t, *J* = 7.4 Hz, 3H).

**1-(bromomethyl)-4-chloro-2-ethoxybenzene (26c)**

Brown oil (70%). ^1^H NMR (400 MHz, CDCl_3_) δ 7.28 (d, *J* = 2.2 Hz, 1H), 6.92 (dd, *J* = 8.1, 2.0 Hz, 1H), 6.87 (d, *J* = 2.0 Hz, 1H), 4.54 (s, 2H), 4.11 (q, *J* = 7.0 Hz, 2H), 1.49 (t, *J* = 7.0 Hz, 3H).

**(2-(bromomethyl)phenyl)(ethyl)sulfane (26d)**

Brown oil (70%). ^1^H NMR (400 MHz, CDCl_3_) δ 7.38 (ddd, *J* = 7.7, 5.8, 1.5 Hz, 2H), 7.25 (ddtd, *J* = 29.3, 15.0, 7.5, 1.5 Hz, 2H), 4.70 (s, 2H), 2.96 (dq, *J* = 26.9, 7.4 Hz, 2H), 1.32 (dt, *J* = 18.6, 7.4 Hz, 3H).

**(2-(bromomethyl)phenyl)(propyl)sulfane (26e)**

Brown oil (70%). ^1^H NMR (400 MHz, CDCl_3_) δ 7.38 (ddd, *J* = 8.0, 4.5, 1.6 Hz, 2H), 7.34 – 7.20 (m, 1H), 7.17 (td, *J* = 7.5, 1.5 Hz, 1H), 4.70 (d, *J* = 1.9 Hz, 2H), 2.95 (td, *J* = 7.3, 1.8 Hz, 2H), 1.77 – 1.60 (m, 2H), 1.04 (td, *J* = 6.9, 4.8 Hz, 3H).

**(2-(bromomethyl)phenyl)(isopropyl)sulfane (26f)**

Brown oil (70%). ^1^H NMR (400 MHz, CDCl_3_) δ 7.53 – 7.43 (m, 1H), 7.46 – 7.40 (m, 1H), 7.34 – 7.19 (m, 2H), 4.75 (s, 1H), 3.46 (hept, *J* = 6.7 Hz, 1H), 1.32 (d, *J* = 6.6 Hz, 6H).

**1-(bromomethyl)-2-ethylbenzene (29a)**

Synthesis was previously reported. Spectra data were in agreement with literature values.^3^

**1-(bromomethyl)-2-propylbenzene (29b)**

Brown oil (40%). ^1^H NMR (400 MHz, CDCl_3_) δ 7.36 – 7.22 (m, 2H), 7.22 – 7.13 (m, 2H), 4.55 (s, 2H), 2.76 – 2.62 (m, 2H), 1.76 – 1.58 (m, 2H), 1.02 (t, *J* = 7.3 Hz, 3H).

**1-(bromomethyl)-2-butylbenzene (29c)**

Brown oil (40%). ^1^H NMR (400 MHz, CDCl_3_) δ 7.33 (dd, *J* = 7.4, 1.5 Hz, 1H), 7.25 (td, *J* = 7.4, 1.5 Hz, 1H), 7.18 (ddd, *J* = 14.4, 7.2, 1.6 Hz, 2H), 4.55 (s, 2H), 2.77 – 2.69 (m, 2H), 1.64 (tt, *J* = 8.0, 6.3 Hz, 2H), 1.44 (h, *J* = 7.4 Hz, 2H), 0.97 (t, *J* = 7.3 Hz, 3H).

**1-(bromomethyl)-2-isobutylbenzene (29d)**

Brown oil (40%). ^1^H NMR (400 MHz, CDCl_3_) δ 7.47 – 7.39 (m, 1H), 7.39 – 7.22 (m, 3H), 4.65 (s, 2H), 2.71 (d, *J* = 7.3 Hz, 2H), 2.04 (dp, *J* = 13.6, 6.8 Hz, 1H), 1.06 (d, *J* = 6.6 Hz, 6H).

**1-(bromomethyl)-2-(3,3,3-trifluoropropyl)benzene (29e)**

Brown oil (10%). ^1^H NMR (400 MHz, CDCl_3_) δ 7.36 – 7.12 (m, 4H), 4.55 (s, 2H), 2.77 – 2.68 (m, 2H), 1.66 (tq, *J* = 9.9, 7.4 Hz, 2H).

**1-fluoro-4-(prop-2-yn-1-yl)benzene (30a)**

Clear oil (250 mg, 52%). ^1^H NMR (400 MHz, CDCl_3_) δ 7.49 – 7.42 (m, 2H), 7.11 – 6.89 (m, 2H), 3.57 (d, *J* = 7.0 Hz, 2H), 2.19 (t, *J* = 2.6 Hz, 1H).

**1-chloro-4-(prop-2-yn-1-yl)benzene (30b)**

Clear oil (208 mg, 58%). ^1^H NMR (400 MHz, CDCl_3_) δ 7.30 – 7.27 (m, 2H), 7.10 - 6.48 (m, 2H), 2.77 (d, *J* = 2.8 Hz, 2H), 1.40 (t, *J* = 2.8 Hz, 1H).

**1-bromo-4-(prop-2-yn-1-yl)benzene (30c)**

Clear oil (238 mg, 41%). ^1^H NMR (400 MHz, CDCl_3_) δ 7.31 – 7.23 (m, 2H), 7.12 – 7.05 (m, 2H), 3.40 (d, *J* = 2.7 Hz, 2H), 2.04 (t, *J* = 2.7 Hz, 1H).

**1-methyl-4-(prop-2-yn-1-yl)benzene (30d)**

Clear oil (256 mg, 44%). ^1^H NMR (400 MHz, CDCl_3_) δ 7.29 (dd, *J* = 8.1, 3.5 Hz, 2H), 7.18 (dd, *J* = 8.2, 2.3 Hz, 2H), 3.61 (d, *J* = 2.7 Hz, 2H), 2.22 (t, *J* = 2.8 Hz, 1H).

**1-ethyl-4-(prop-2-yn-1-yl)benzene (30e)**

Clear oil (206 mg, 62%). ^1^H NMR (400 MHz, CDCl_3_) δ 7.27 (d, *J* = 7.7 Hz, 1H), 7.16 (d, *J* = 7.9 Hz, 2H), 3.58 (d, *J* = 2.7 Hz, 2H), 2.17 (t, *J* = 2.7 Hz, 1H).

**1-methoxy-4-(prop-2-yn-1-yl)benzene (30f)**

Clear oil (231 mg, 64%). ^1^H NMR (400 MHz, CDCl_3_) δ 7.10 – 7.04 (m, 2H), 6.71 – 6.66 (m, 2H), 3.36 (d, *J* = 2.7 Hz, 2H), 1.99 (dt, *J* = 2.9, 1.5 Hz, 1H).

**1,2-dichloro-4-(prop-2-yn-1-yl)benzene (30g)**

Clear oil (180 mg, 47%). ^1^H NMR (400 MHz, CDCl_3_) δ 7.26 (d, *J* = 2.1 Hz, 1H), 7.18 (s, 1H), 6.98 (dd, *J* = 8.3, 2.1 Hz, 1H), 3.37 (d, *J* = 2.8 Hz, 2H), 2.04 (d, *J* = 2.7 Hz, 1H).

**1-chloro-3-(prop-2-yn-1-yl)benzene (30h)**

Clear oil (251 mg, 51%). ^1^H NMR (400 MHz, CDCl_3_) δ 7.37 (s, 1H), 7.22–7.25 (m, 3H), 3.59 (s, 2H), 2.22 (t, J = 2.6 Hz, 1H).

******

**1-chloro-2-(prop-2-yn-1-yl)benzene (30i)**

Clear oil (233 mg, 63%). ^1^H NMR (400 MHz, CDCl_3_) δ 7.62 (dd, J = 7.4, 1.7 Hz, 1H), 7.36 (dd, J = 8.0, 1.1 Hz, 1H), 7.26–7.29 (m, 1H), 7.20–7.23 (m, 1H), 3.69 (s, 2H), 2.24 (t, J = 2.6 Hz, 1H).

******

**1-ethoxy-2-(prop-2-yn-1-yl)benzene (30j)**

Clear oil (203 mg, 53%). ^1^H NMR (400 MHz, CDCl_3_) δ 7.40 (td, *J* = 7.4, 1.8 Hz, 1H), 7.13 – 6.93 (m, 1H), 6.87 – 6.79 (m, 1H), 6.79 – 6.63 (m, 1H), 4.00 (q, *J* = 7.2 Hz, 2H), 3.48 (d, *J* = 2.8 Hz, 2H), 2.02 (t, J = 2.7 Hz, 1H), 1.24 – 1.11 (m, 3H).

**4-chloro-2-ethoxy-1-(prop-2-yn-1-yl)benzene (30k)**

Clear oil (159 mg, 31%). ^1^H NMR (400 MHz, CDCl_3_) δ 7.42 (dd, *J* = 8.2, 4.3 Hz, 1H), 6.92 (dd, *J* = 8.1, 1.9 Hz, 1H), 6.80 (dd, *J* = 6.0, 2.0 Hz, 1H), 4.02 (q, *J* = 6.9 Hz, 2H), 3.52 (d, *J* = 2.7 Hz, 2H), 2.17 (t, *J* = 2.7 Hz, 1H), 1.42 (t, *J* = 6.9 Hz, 3H).

******

**1-(prop-2-yn-1-yl)-2-propoxybenzene (30l)**

Clear oil (198 mg, 51%). ^1^H NMR (400 MHz, CDCl_3_) δ 7.51 (dd, *J* = 7.5, 1.8 Hz, 1H), 7.28 – 7.17 (m, 1H), 6.94 (td, *J* = 7.5, 1.2 Hz, 1H), 6.83 (dd, *J* = 8.2, 1.3 Hz, 1H), 3.94 (td, *J* = 6.4, 1.8 Hz, 2H), 3.60 (d, *J* = 2.7 Hz, 2H), 2.16 (t, *J* = 2.3 Hz, 1H), 1.89 – 1.72 (m, 2H), 1.05 (td, *J* = 7.5, 1.7 Hz, 3H).

******

**ethyl(2-(prop-2-yn-1-yl)phenyl)sulfane (30m)**

Yellow oil (189 mg, 39%). ^1^H NMR (400 MHz, CDCl_3_) δ 7.69 – 7.59 (m, 1H), 7.35 (ddd, *J* = 10.0, 5.6, 1.8 Hz, 1H), 7.26 – 7.15 (m, 1H), 7.15 – 6.99 (m, 1H), 3.72 (d, *J* = 2.7 Hz, 2H), 3.01 – 2.87 (q, *J* = 6.8 Hz, 2H), 2.21 (t, *J* = 2.7 Hz, 1H), 1.51 – 1.18 (t, *J* = 2.8 Hz, 3H).

******

**(2-(prop-2-yn-1-yl)phenyl)(propyl)sulfane (30n)**

Yellow oil (120 mg, 40%). ^1^H NMR (400 MHz, CDCl_3_) δ 7.56 (dd, *J* = 6.9, 2.4 Hz, 1H), 7.41 – 7.29 (m, 2H), 7.27 – 7.14 (m, 1H), 7.18 (s, 1H), 3.72 (d, *J* = 2.8 Hz, 2H), 2.94 – 2.73 (t, *J* = 6.8 Hz, 2H), 2.22 (t, *J* = 2.7 Hz, 1H), 1.74 – 1.64 (m, 2H), 1.08 – 0.95 (t, *J* = 1.8 Hz, 3H).

******

**isopropyl(2-(prop-2-yn-1-yl)phenyl)sulfane (30o)**

Yellow oil (89 mg, 42%). ^1^H NMR (400 MHz, CDCl_3_) δ 7.59 (d, *J* = 7.3 Hz, 1H), 7.43 (d, *J* = 7.4 Hz, 2H), 7.28 – 7.16 (m, 1H), 3.79 (d, *J* = 2.8 Hz, 2H), 3.42 – 3.30 (m, 1H), 2.20 (d, *J* = 2.9 Hz, 1H), 1.33 – 1.20 (d, *J* = 6.5 Hz, 9H).

******

**1-ethyl-2-(prop-2-yn-1-yl)benzene (30p)**

Clear oil (258 mg, 61%). ^1^H NMR (400 MHz, CDCl_3_) δ 7.51 (dd, *J* = 7.7, 1.9 Hz, 1H), 7.26 (tq, *J* = 4.9, 2.2 Hz, 3H), 3.63 (d, *J* = 2.7 Hz, 2H), 2.73 (q, *J* = 7.6 Hz, 2H), 2.23 (t, *J* = 2.7 Hz, 1H), 1.30 (t, *J* = 7.5 Hz, 3H).

**1-(prop-2-yn-1-yl)-2-propylbenzene (30q)**

Clear oil (259mg, 61%). ^1^H NMR (400 MHz, CDCl_3_) δ 7.51 (dd, *J* = 7.7, 1.9 Hz, 1H), 7.26 (tq, *J* = 4.9, 2.2 Hz, 3H), 3.58 (d, *J* = 2.7 Hz, 2H), 2.68 – 2.56 (m, 2H), 2.18 (t, *J* = 2.8 Hz, 1H), 1.33 – 1.23 (m, 2H), 1.30 (t, *J* = 7.5 Hz, 3H).

**1-butyl-2-(prop-2-yn-1-yl)benzene (30r)**

Clear oil (200 mg, 60%). ^1^H NMR (400 MHz, CDCl_3_) δ 7.45 (q, *J* = 5.0 Hz, 1H), 7.27 – 7.11 (m, 3H), 3.58 (d, *J* = 2.7 Hz, 2H), 2.68 – 2.56 (m, 2H), 2.18 (t, *J* = 2.8 Hz, 1H), 1.64 – 1.50 (m, 2H), 1.41 (h, *J* = 7.4 Hz, 2H), 1.26 (t, *J* = 7.1 Hz, 3H).

**1-isobutyl-2-(prop-2-yn-1-yl)benzene (30s)**

Clear oil (166 mg, 45%). ^1^H NMR (400 MHz, CDCl_3_) δ 7.52 – 7.43 (m, 1H), 7.26 – 7.15 (m, 2H), 7.14 (td, *J* = 9.3, 5.0 Hz, 1H), 3.58 (d, *J* = 2.7 Hz, 2H), 2.51 (d, *J* = 7.2 Hz, 2H), 2.18 (t, *J* = 2.7 Hz, 1H), 1.87 (dp, *J* = 13.5, 6.7 Hz, 1H), 0.94 (d, *J* = 6.6 Hz, 6H).

^^

**1-(prop-2-yn-1-yl)-2-(3,3,3-trifluoropropyl)benzene (30t)**

Clear oil (80 mg, 55%). ^1^H NMR (400 MHz, CDCl_3_) δ 7.45 (q, *J* = 4.5 Hz, 1H), 7.19 (tt, *J* = 5.3, 2.6 Hz, 3H), 3.64 – 3.55 (d, 2H), 2.61 (t, *J* = 7.4 Hz, 2H), 2.17 (t, *J* = 2.8 Hz, 1H), 1.26 (t, *J* = 7.1 Hz, 2H).

**methyl 3-chloro-2-(2-(5-(4-fluorobenzyl)-1H-1,2,3-triazol-1-yl)ethyl)-4,6-dihydroxybenzoate (31)**

White amorphous solid (9 mg, 67%). ^1^H NMR (400 MHz, CDCl_3_) δ 11.32 (s, 1H), 7.34 (s, 1H), 7.00 – 6.90 (m, 4H), 6.58 (s, 1H), 4.36 (t, *J* = 7.7 Hz, 2H), 3.94 (s, 3H), 3.81 (s, 2H), 3.60 (t, *J* = 7.8 Hz, 2H). ^13^C NMR (101 MHz, CDCl_3_) δ 170.34, 163.29, 156.40, 137.49, 136.89, 135.25, 134.36, 133.45, 129.90, 129.81, 116.14, 115.93, 103.93, 53.05, 46.99, 33.52, 28.36. Purity: 94%. HRMS (ESI) m/z [M+1] calculated for C_19_H_17_ClFN_3_O_4_, 406.0964, found 406.0957.

**methyl 3-chloro-2-(2-(5-(4-chlorobenzyl)-1H-1,2,3-triazol-1-yl)ethyl)-4,6-dihydroxybenzoate (32)**

White amorphous solid (9 mg, 55%). ^1^H NMR (400 MHz, CDCl_3_) δ 11.32 (s, 1H), 7.35 (s, 1H), 7.26 – 7.18 (m, 2H), 6.95 (d, *J* = 8.3 Hz, 2H), 6.58 (s, 1H), 4.39 – 4.31 (m, 2H), 3.94 (s, 3H), 3.82 (s, 2H), 3.59 (dd, *J* = 8.7, 6.6 Hz, 2H). ^13^C NMR (101 MHz, CDCl_3_) δ 170.27, 163.27, 156.50, 137.70, 135.25, 134.36, 133.45, 133.31, 129.62, 129.18, 114.36, 107.15, 103.84, 53.01, 46.71, 33.58, 28.45. Purity: 92% HRMS (ESI) m/z [M+1] calculated for C_19_H_17_Cl_2_N_3_O_4_, 422.0669, found 422.0669.

**methyl 2-(2-(5-(4-bromobenzyl)-1*H*-1,2,3-triazol-1-yl)ethyl)-3-chloro-4,6-dihydroxybenzoate (33)**

White amorphous solid (5 mg, 52%). ^1^H NMR (400 MHz, CDCl_3_) δ 11.38 (s, 1H), 7.44 (dd, *J* = 8.0, 6.1 Hz, 3H), 6.95 (d, *J* = 8.0 Hz, 2H), 6.65 (s, 1H), 4.41 (t, *J* = 7.7 Hz, 2H), 4.01 (s, 3H), 3.87 (s, 2H), 3.66 (t, *J* = 7.7 Hz, 2H). ^13^C NMR (101 MHz, CDCl_3_) δ 170.26, 163.27, 156.44, 137.71, 134.92, 133.53, 132.13, 129.97, 121.32, 114.30, 107.18, 103.83, 98.86, 53.01, 46.69, 33.57, 28.52. Purity: 92% HRMS (ESI) m/z [M+1] calculated for C_20_H_20_ClN_3_O_4_, 466.0164, found 466.0158.

**methyl 3-chloro-4,6-dihydroxy-2-(2-(5-(4-methylbenzyl)-1H-1,2,3-triazol-1-yl)ethyl)benzoate (34)**

White amorphous solid (15 mg, 80%). ^1^H NMR (400 MHz, CDCl_3_) δ 11.42 (s, 1H), 7.44 (s, 1H), 7.12 (d, *J* = 7.7 Hz, 2H), 6.96 (d, *J* = 7.7 Hz, 2H), 6.65 (s, 1H), 4.48 – 4.39 (m, 2H), 4.00 (s, 3H), 3.89 (s, 2H), 3.70 – 3.62 (m, 2H), 2.33 (s, 3H). ^13^C NMR (101 MHz, CDCl_3_) δ 170.32, 163.29, 156.56, 137.63, 137.15, 136.50, 132.75, 132.47, 129.71, 128.16, 114.45, 107.08, 103.83, 53.01, 46.90, 33.53, 28.68, 21.05. Purity: 96% HRMS (ESI) m/z [M+1] calculated for C_20_H_20_ClN_3_O_4_, 402.1215, found 402.1217.

******

**methyl 3-chloro-2-(2-(5-(4-ethylbenzyl)-1H-1,2,3-triazol-1-yl)ethyl)-4,6-dihydroxybenzoate (35)**

White amorphous solid (17 mg, 83%). ^1^H NMR (400 MHz, CDCl_3_) δ 11.41 (s, 1H), 7.46 (s, 1H), 7.15 (d, *J* = 7.7 Hz, 2H), 7.00 (d, *J* = 7.7 Hz, 2H), 6.68 (s, 1H), 4.50 – 4.41 (m, 2H), 4.00 (s, 3H), 3.90 (s, 2H), 3.67 (t, *J* = 7.7 Hz, 2H), 2.62 (q, *J* = 7.6 Hz, 2H), 1.30 – 1.18 (m, 3H). ^13^C NMR (101 MHz, CDCl_3_) δ 170.89, 151.11, 150.69, 143.69, 140.04, 137.41, 134.49, 134.17, 129.32, 114.75, 103.90, 103.87, 103.09, 64.23, 61.69, 32.35, 29.95, 26.43, 10.96. Purity: 91% HRMS (ESI) m/z [M+1] calculated for C_21_H_22_ClN_3_O_4_, 416.1372, found 416.1383.

**methyl 3-chloro-4,6-dihydroxy-2-(2-(5-(4-methoxybenzyl)-1H-1,2,3-triazol-1-yl)ethyl)benzoate (36)**

White amorphous solid (22 mg, 49%). ^1^H NMR (400 MHz, CDCl_3_) δ 11.40 (s, 1H), 7.53 (s, 1H), 7.01 (d, *J* = 8.6 Hz, 2H), 6.92 – 6.84 (m, 2H), 6.68 (s, 1H), 4.53 – 4.45 (m, 2H), 4.03 (s, 3H), 3.89 (s, 2H), 3.82 (s, 3H), 3.74 – 3.65 (m, 2H). ^13^C NMR (101 MHz, CDCl_3_) δ 170.33, 163.30, 157.96, 137.74, 137.25, 136.80, 134.15, 132.40, 129.88, 129.10, 120.49, 107.09, 104.33, 61.82, 54.22, 50.90, 35.09, 27.19. Purity: 97% HRMS (ESI) m/z [M+1] calculated for C_20_H_20_ClN_3_O_5_, 418.1164, found 418.1167.

******

**methyl 3-chloro-2-(2-(5-(3,4-dichlorobenzyl)-1H-1,2,3-triazol-1-yl)ethyl)-4,6-dihydroxybenzoate (37)**

White amorphous solid (8 mg, 44%). ^1^H NMR (400 MHz, CDCl_3_) δ 11.33 (s, 1H), 7.46 (s, 1H), 7.39 (d, *J* = 8.3 Hz, 1H), 7.18 (d, *J* = 2.1 Hz, 1H), 6.92 (dd, *J* = 8.2, 2.1 Hz, 1H), 6.65 (s, 1H), 4.47 – 4.38 (m, 2H), 4.02 (s, 3H), 3.87 (s, 2H), 3.70 – 3.62 (m, 2H), 1.61 (s, 1H), 1.30 – 1.22 (m, 1H), 0.91 – 0.86 (m, 1H). ^13^C NMR (101 MHz, CDCl_3_) δ 170.50, 160.02, 156.47, 153.71, 136.18, 135.30, 133.39, 131.81, 131.16, 130.30, 127.65, 122.89, 116.96, 114.35, 107.04, 53.21, 47.18, 33.42, 28.24. Purity: 94% HRMS (ESI) m/z [M+1] calculated for C_19_H_16_Cl_3_N_3_O_4_, 418.1164, found 418.1167.

******

**methyl 3-chloro-2-(2-(5-(3-chlorobenzyl)-1H-1,2,3-triazol-1-yl)ethyl)-4,6-dihydroxybenzoate (38)**

White amorphous solid (10 mg, 52%). ^1^H NMR (400 MHz, CDCl_3_) δ 10.92 (s, 1H), 7.36 – 7.28 (m, 4H), 7.11 (s, 1H), 6.22 (s, 1H), 4.28 – 4.12 (m, 2H), 3.90 (s, 3H), 3.81 (s, 2H), 3.03 (dd, *J* = 7.4, 5.6 Hz, 2H). ^13^C NMR (101 MHz, CDCl_3_) δ 170.04, 162.70, 160.04, 144.64, 138.35, 134.51, 134.20, 130.01, 128.84, 119.44, 115.08, 107.44, 104.68, 51.51, 48.05, 37.48, 25.40. Purity: 95% HRMS (ESI) m/z [M+1] calculated for C_19_H_17_Cl_2_N_3_O_4_, 422.0712, found 422.0753.

******

**methyl 3-chloro-2-(2-(5-(2-chlorobenzyl)-1H-1,2,3-triazol-1-yl)ethyl)-4,6-dihydroxybenzoate (39)**

White amorphous solid (10 mg, 68%). ^1^H NMR (400 MHz, CDCl_3_) δ 11.42 (s, 1H), 7.64 (d, 1H), 7.39 (s, 1H), 7.12 – 7.18 (m, 3H), 6.22 (s, 1H), 4.06 (dd, 2H), 3.92 (s, 3H), 3.80 (s, 2H), 3.33 (dd, *J* = 7.2, 5.6 Hz, 2H). ^13^C NMR (101 MHz, CDCl_3_) δ 171.04, 163.75, 161.70, 146.54, 144.63, 138.11, 134.49, 130.45, 128.76, 127.10, 126.71, 115.10, 107.04, 104.38, 54.61, 51.55, 48.05, 27.25, 25.40. Purity: 95% HRMS (ESI) m/z [M+1] calculated for C_19_H_17_Cl_2_N_3_O_4_, 422.0712, found 422.0753.

******

**methyl 3-chloro-2-(2-(5-(2-ethoxybenzyl)-1H-1,2,3-triazol-1-yl)ethyl)-4,6-dihydroxybenzoate (40)**

White amorphous solid (21 mg, 63%). ^1^H NMR (400 MHz, CDCl_3_) δ 11.43 (s, 1H), 7.43 (s, 1H), 7.23 (m, 1H), 6.97 (d, *J* = 7.4 Hz, 1H), 6.92 – 6.82 (m, 2H), 6.64 (s, 1H), 4.59 – 4.51 (m, 2H), 4.02 (s, 3H), 4.08 – 3.96 (m, 3H), 3.94 (s, 2H), 3.72 – 3.64 (m, 2H), 1.34 (t, *J* = 7.0 Hz, 3H). ^13^C NMR (101 MHz, CDCl_3_) δ 170.30, 163.37, 156.49, 156.41, 135.08, 137.77, 129.41, 128.78, 124.46, 120.60, 114.42, 111.32, 107.12, 105.55, 103.78, 63.52, 53.01, 33.66, 33.60, 23.79, 14.84. Purity: 97% HRMS (ESI) m/z [M+1] calculated for C_21_H_22_ClN_3_O_5_, 432.1321, found 432.1331.

******

**methyl 3-chloro-2-(2-(5-(4-chloro-2-ethoxybenzyl)-1H-1,2,3-triazol-1-yl)ethyl)-4,6-dihydroxybenzoate (41)**

White amorphous solid (6 mg, 32%). ^1^H NMR (400 MHz, CDCl_3_) δ 11.41 (s, 1H), 7.41 (s, 1H), 6.89 – 6.82 (m, 3H), 6.64 (s, 1H), 4.56 – 4.48 (m, 2H), 4.08 - 3.96 (m, *J* = 11.5 Hz, 5H), 3.88 (s, 2H), 3.71 – 3.63 (m, 2H), 1.35 (t, *J* = 7.0 Hz, 3H). ^13^C NMR (101 MHz, CDCl_3_) δ 170.32, 163.39, 156.94, 156.35, 137.54, 135.85, 134.26, 134.17, 132.76, 130.05, 122.98, 120.61, 112.14, 107.17, 103.84, 64.03, 53.02, 46.83, 33.62, 23.39, 14.67. Purity: 95% HRMS (ESI) m/z [M+1] calculated for C_21_H_21_Cl_2_N_3_O_5_, 466.0931, found 466.0939.

******

**methyl 3-chloro-4,6-dihydroxy-2-(2-(5-(2-propoxybenzyl)-1H-1,2,3-triazol-1-yl)ethyl)benzoate (42)**

White amorphous solid (13 mg, 66%). ^1^H NMR (400 MHz, CDCl_3_) δ 11.46 (s, 1H), 7.39 (s, 1H), 7.28 – 7.19 (m, 1H), 6.97 (dd, *J* = 7.4, 1.8 Hz, 1H), 6.87 (dd, *J* = 10.8, 7.8 Hz, 2H), 6.71 (s, 1H), 4.56 (t, *J* = 7.8 Hz, 2H), 4.01 (d, *J* = 9.1 Hz, 5H), 3.90 (t, *J* = 6.5 Hz, 2H), 3.68 (dd, *J* = 9.4, 6.4 Hz, 2H), 1.74 (h, *J* = 7.0 Hz, 2H), 0.95 (t, *J* = 7.4 Hz, 3H). ^13^C NMR (101 MHz, CDCl_3_) δ 170.66, 163.20, 157.60, 156.56, 137.72, 132.98, 129.43, 128.73, 124.53, 120.51, 114.97, 111.28, 106.53, 103.75, 102.68, 69.47, 52.92, 46.78, 33.62, 24.70, 22.56, 10.57. Purity: 93% HRMS (ESI) m/z [M+1] calculated for C_22_H_24_ClN_3_O_5_, 446.1477, found 446.1475.

^^

**methyl 3-chloro-2-(2-(5-(2-(ethylthio)benzyl)-1H-1,2,3-triazol-1-yl)ethyl)-4,6-dihydroxybenzoate (43)**

Tan amorphous solid (5 mg, 34%). ^1^H NMR (400 MHz, CDCl_3_) δ 11.40 (s, 1H), 7.40 – 7.33 (m, 2H), 7.29 (d, *J* = 7.5 Hz, 1H), 7.19 – 7.11 (m, 1H), 6.97 (d, *J* = 7.6 Hz, 1H), 6.66 (s, 1H), 4.58 (t, *J* = 7.8 Hz, 2H), 4.04 (d, *J* = 9.8 Hz, 5H), 3.76 – 3.67 (m, 2H), 2.90 (q, *J* = 7.3 Hz, 2H), 1.27 (t, *J* = 7.4 Hz, 3H). ^13^C NMR (101 MHz, CDCl_3_) δ 170.57, 163.21, 157.37, 139.76, 137.63, 136.10, 132.99, 131.91, 129.11, 129.02, 128.04, 126.27, 114.93, 106.68, 103.81, 52.97, 46.95, 33.59, 28.15, 27.38, 14.17. Purity: 96% HRMS (ESI) m/z [M+1] calculated for C_21_H_22_ClN_3_O_4_S, 448.1092, found 448.1117.

**methyl 3-chloro-4,6-dihydroxy-2-(2-(5-(2-(propylthio)benzyl)-1*H*-1,2,3-triazol-1-yl)ethyl)benzoate (44)**

Tan amorphous solid (3 mg, 28%). ^1^H NMR (400 MHz, CDCl_3_) δ 11.43 (s, 1H), 7.38 – 7.31 (m, 2H), 7.13 (t, *J* = 7.3 Hz, 2H), 6.94 (d, *J* = 7.6 Hz, 1H), 6.63 (s, 1H), 4.53 (t, *J* = 7.8 Hz, 2H), 4.04 (d, *J* = 7.5 Hz, 5H), 3.70 (t, *J* = 7.8 Hz, 2H), 2.85 (q, *J* = 7.1 Hz, 2H), 1.63 (h, *J* = 7.2 Hz, 3H), 0.99 (t, *J* = 7.4 Hz, 2H). ^13^C NMR (101 MHz, CDCl_3_) δ 170.38, 163.36, 158.34, 156.39, 140.52, 137.51, 136.35, 129.03, 128.90, 128.18, 126.44, 126.19, 114.42, 110.63, 103.83, 53.05, 47.12, 35.62, 33.58, 27.32, 22.38, 13.52. Purity: 99% HRMS (ESI) m/z [M+1] calculated for C_21_H_22_ClN_3_O_4_S, 462.1249, found 462.1255.

**methyl 3-chloro-4,6-dihydroxy-2-(2-(5-(2-(isopropylthio)benzyl)-1*H*-1,2,3-triazol-1-yl)ethyl)benzoate (45)**

Tan amorphous solid (6 mg, 33%). ^1^H NMR (400 MHz, CDCl_3_) δ 11.41 (s, 1H), 7.46 (d, *J* = 7.6 Hz, 1H), 7.37 (s, 1H), 7.28 (t, *J* = 5.8 Hz, 1H), 7.24 – 7.16 (m, 1H), 7.01 (d, *J* = 7.7 Hz, 1H), 6.65 (s, 1H), 4.57 (t, *J* = 7.8 Hz, 2H), 4.10 (s, 2H), 4.03 (s, 3H), 3.71 (t, *J* = 7.8 Hz, 2H), 3.33 (p, *J* = 6.6 Hz, 1H), 1.24 (d, *J* = 6.6 Hz, 6H). ^13^C NMR (101 MHz, CDCl_3_) δ 170.29, 163.35, 156.44, 143.42, 137.29, 136.58, 135.29, 132.31, 132.03, 129.30, 128.29, 127.33, 107.17, 103.94, 94.28, 53.09, 47.22, 38.55, 33.49, 27.65, 23.04. Purity: 96% HRMS (ESI) m/z [M+1] calculated for C_21_H_22_ClN_3_O_4_S, 462.1559, found 462.1580.

^^

**methyl 3-chloro-2-(2-(5-(2-ethylbenzyl)-1H-1,2,3-triazol-1-yl)ethyl)-4,6-dihydroxybenzoate (46)**

White amorphous solid (8 mg, 59%). ^1^H NMR (400 MHz, CDCl_3_) δ 11.39 (s, 1H), 7.30 – 7.20 (m, 3H), 7.19 – 7.10 (m, 1H), 6.88 (d, *J* = 7.6 Hz, 1H), 6.67 (d, *J* = 2.9 Hz, 1H), 4.52 (t, *J* = 7.7 Hz, 2H), 4.02 (d, *J* = 1.4 Hz, 3H), 3.89 (s, 2H), 3.71 (t, *J* = 7.6 Hz, 2H), 2.56 (q, *J* = 7.6 Hz, 2H), 1.18 (tt, *J* = 7.5, 2.1 Hz, 3H).  ^13^C NMR (101 MHz, CDCl_3_) δ 170.33, 163.23, 156.95, 141.94, 137.61, 136.55, 133.19, 132.91, 128.94, 128.84, 127.90, 126.52, 114.61, 106.93, 103.88, 53.00, 46.92, 33.54, 26.38, 25.66, 14.74. Purity: 93% HRMS (ESI) m/z [M+1] calculated for C_21_H_22_ClN_3_O_4_, 416.1372, found 416.1374.

***^^***

**methyl 3-chloro-4,6-dihydroxy-2-(2-(5-(2-propylbenzyl)-1H-1,2,3-triazol-1-yl)ethyl)benzoate (47)**

White amorphous solid (9 mg, 60%). ^1^H NMR (400 MHz, CDCl_3_) δ 11.32 (s, 1H), 7.16 (dt, *J* = 7.3, 3.3 Hz, 3H), 7.07 (td, *J* = 7.0, 2.5 Hz, 1H), 6.81 (d, *J* = 7.6 Hz, 1H), 6.62 (d, *J* = 1.5 Hz, 1H), 4.49 – 4.41 (m, 2H), 3.96 (s, 3H), 3.86 (s, 2H), 3.68 – 3.59 (m, 2H), 2.45 (dd, *J* = 9.1, 6.5 Hz, 2H), 1.50 (h, *J* = 7.4 Hz, 2H), 0.88 (t, *J* = 7.3 Hz, 3H). ^13^C NMR (101 MHz, CDCl_3_) δ 170.28, 163.23, 156.95, 140.50, 137.47, 136.84, 133.23, 132.64, 129.82, 128.95, 127.73, 126.59, 114.60, 106.92, 103.92, 53.02, 47.03, 34.92, 33.53, 26.41, 23.73, 14.21. Purity: 98% HRMS (ESI) m/z [M+1] calculated for C_22_H_24_ClN_3_O_4_, 452.1384, found 452.1359.

**methyl 2-(2-(5-(2-butylbenzyl)-1*H*-1,2,3-triazol-1-yl)ethyl)-3-chloro-4,6-dihydroxybenzoate (48)**

White amorphous solid (11 mg, 64%). ^1^H NMR (400 MHz, CDCl_3_) δ 11.37 (s, 1H), 7.34 (s, 1H), 7.29 – 7.19 (m, 2H), 7.15 (td, *J* = 6.9, 2.4 Hz, 1H), 6.87 (d, *J* = 7.5 Hz, 1H), 6.68 (s, 1H), 4.54 (t, *J* = 7.7 Hz, 2H), 4.03 (s, 3H), 3.95 (s, 2H), 3.71 (t, *J* = 7.6 Hz, 2H), 2.57 – 2.49 (m, 2H), 1.52 (p, *J* = 7.5 Hz, 2H), 1.35 (h, *J* = 7.3 Hz, 2H), 0.91 (t, *J* = 7.3 Hz, 3H). ^13^C NMR (101 MHz, CDCl_3_) δ 170.41, 163.21, 157.17, 140.75, 137.60, 136.50, 133.40, 133.07, 129.73, 128.97, 127.66, 126.50, 114.70, 106.80, 103.85, 52.98, 46.83, 33.59, 32.79, 32.61, 26.42, 22.70, 14.00. Purity: 95% HRMS (ESI) m/z [M+1] calculated for C_23_H_26_ClN_3_O_4_, 444.1685, found 444.1696.

***^^***

**methyl 3-chloro-4,6-dihydroxy-2-(2-(5-(2-isobutylbenzyl)-1H-1,2,3-triazol-1-yl)ethyl)benzoate (49)**

White amorphous solid (11 mg, 60%). ^1^H NMR (400 MHz, CDCl_3_) δ 11.42 (s, 1H), 7.26 – 7.10 (m, 4H), 6.88 (d, *J* = 7.5 Hz, 1H), 6.69 (s, 1H), 4.50 (dd, *J* = 10.4, 5.3 Hz, 2H), 4.03 (s, 3H), 3.95 (s, 2H), 3.70 (dd, *J* = 9.2, 6.4 Hz, 2H), 2.44 (d, *J* = 7.3 Hz, 2H), 1.79 (hept, *J* = 6.8 Hz, 1H), 0.90 (d, *J* = 6.6 Hz, 6H). ^13^C NMR (101 MHz, CDCl_3_) δ 170.46, 163.21, 157.21, 139.62, 137.67, 136.37, 133.87, 133.34, 130.79, 128.98, 127.32, 126.57, 114.73, 106.78, 103.82, 52.98, 46.72, 42.23, 33.63, 29.45, 26.44, 26.42, 22.56. Purity: 92% HRMS (ESI) m/z [M+1] calculated for C_23_H_26_ClN_3_O_4_, 444.1685, found 444.1680.

**methyl 3-chloro-4,6-dihydroxy-2-(2-(5-(2-(3,3,3-trifluoropropyl)benzyl)-1*H*-1,2,3-triazol-1-yl)ethyl)benzoate (50)**

white amorphous solid (2 mg, 30%). ^1^H NMR (400 MHz, CDCl_3_) δ 11.41 (s, 1H), 7.28 – 7.18 (m, 3H), 7.13 (td, *J* = 6.7, 2.9 Hz, 1H), 6.87 (d, *J* = 7.6 Hz, 1H), 6.67 (s, 1H), 4.49 (t, *J* = 7.8 Hz, 2H), 4.03 (s, 3H), 3.93 (s, 2H), 3.74 – 3.65 (m, 2H), 2.54 (t, *J* = 7.9 Hz, 2H), 1.53 (t, *J* = 7.7 Hz, 2H). ^13^C NMR (101 MHz, CDCl_3_) δ 170.39, 163.26, 156.79, 140.78, 137.71, 136.19, 133.54, 133.39, 129.69, 128.94, 127.61, 126.47, 120.07, 114.52, 107.01, 103.81, 52.99, 46.69, 33.61, 32.86, 31.79, 22.57. Purity: 92% HRMS (ESI) m/z [M+1] calculated for C_23_H_26_ClN_3_O_4_, 484.1890, found 484.1880.

**References:**

1. Clevenger, R.C.; Blagg, B.S.J., *Org. Lett.* **2004**, 6, 24, 4459–4462.
2. Bhagat, S.B.; Telvekar, V.N., *Syn. Lett.* **2018**, 29(07), 874–879.
3. Hayes, R.; Li, K.; Leeming, P.; Wallace, T.W.; Williams, R.C., *Tetrahedron,* **1999**, 55, 44, 12907–12928.
4. Ernst, J.T. et al., *J. Med. Chem.* **2014**, 57, 3382−3400

**Fluorescence Polarization Assay**

| **Compound 47 Polarization Read 1** | | | | **DMSO** | **Tracer** |
| --- | --- | --- | --- | --- | --- |
| 10 µM | 170 | 191 | 174 | 323 | 195 |
| 1 µM | 189 | 179 | 182 | 324 | 190 |
| 0.1 µM | 247 | 250 | 234 | 324 | 191 |
| 0.01 µM | 298 | 289 | 285 | 332 | 182 |
| **Compound 47 Polarization Read 2** | | | | **DMSO** | **Tracer** |
| 10 µM | 172 | 178 | 176 | 314 | 152 |
| 1 µM | 178 | 177 | 180 | 319 | 176 |
| 0.1 µM | 234 | 231 | 237 | 319 | 170 |
| 0.01 µM | 278 | 288 | 270 | 316 | 187 |
| **Compound 47 Polarization Read 3** | | | | **DMSO** | **Tracer** |
| 10 µM | 169 | 185 | 177 | 306 | 161 |
| 1 µM | 185 | 186 | 185 | 311 | 147 |
| 0.1 µM | 220 | 220 | 210 | 308 | 166 |
| 0.01 µM | 275 | 284 | 279 | 304 | 163 |

| **47 [µM]** | **% Bound** |
| --- | --- |
| 10 | 2% |
| 1 | 6% |
| 0.1 | 41% |
| 0.01 | 76% |

**
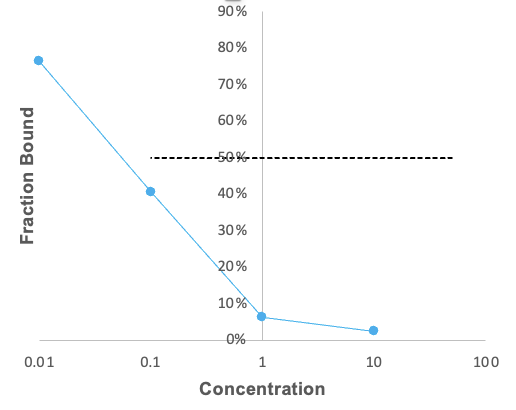
**

**K_d_ = [I50]/([L50]/K + [P0]/K +1)^4^**

[I50] = concentration of free inhibitor at 50% inhibition, [L50] = concentration of free fluorescence labeled ligand molecule at 50% inhibition, [P0] = concentration of free protein at 0% inhibition, K = dissociation constant of the protein−ligand complex.^4^

**Spectra Data**

**methyl 2-(but-3-yn-1-yl)-4,6-bis((tert-butyldimethylsilyl)oxy)-3-chlorobenzoate (8)**

**^1^H NMR**

**
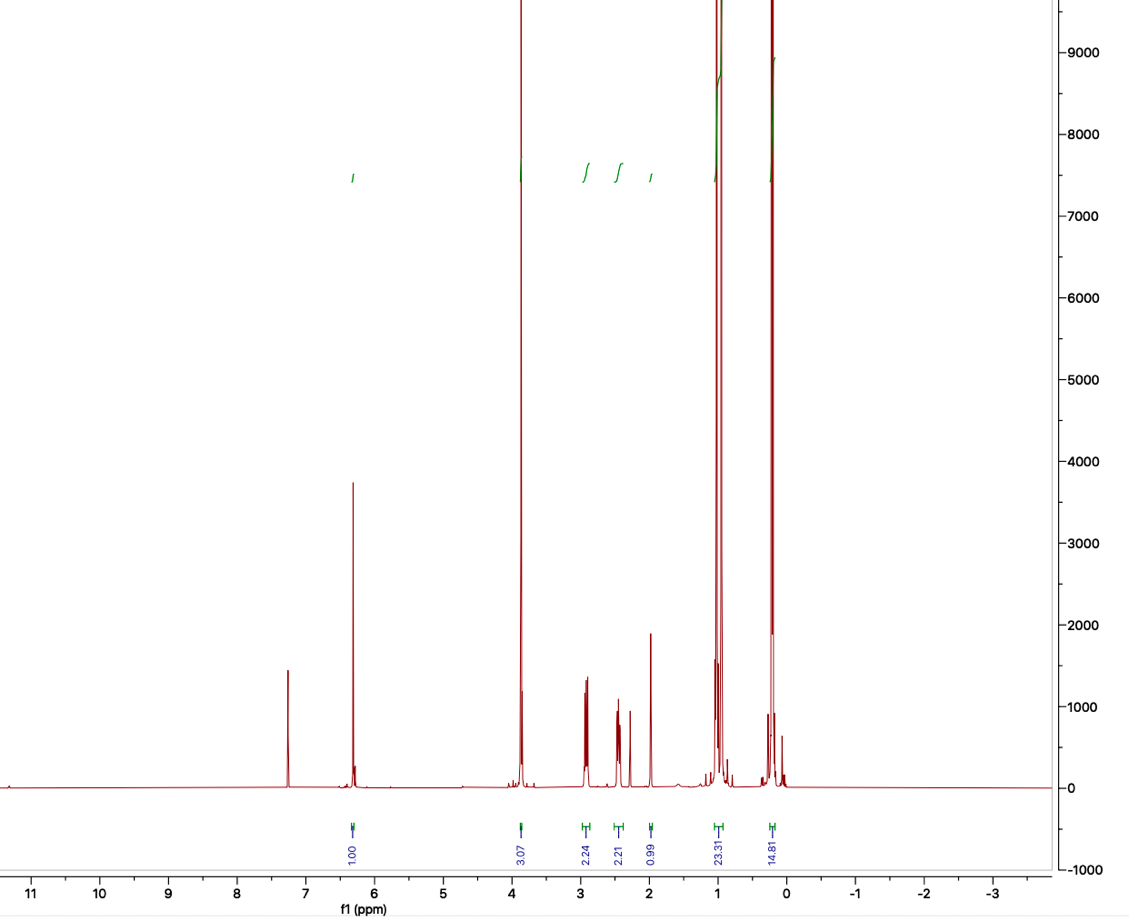
**

**methyl 2-(but-3-yn-1-yl)-4,6-bis((tert-butyldimethylsilyl)oxy)-3-chlorobenzoate (8)**

**^13^C NMR**

**
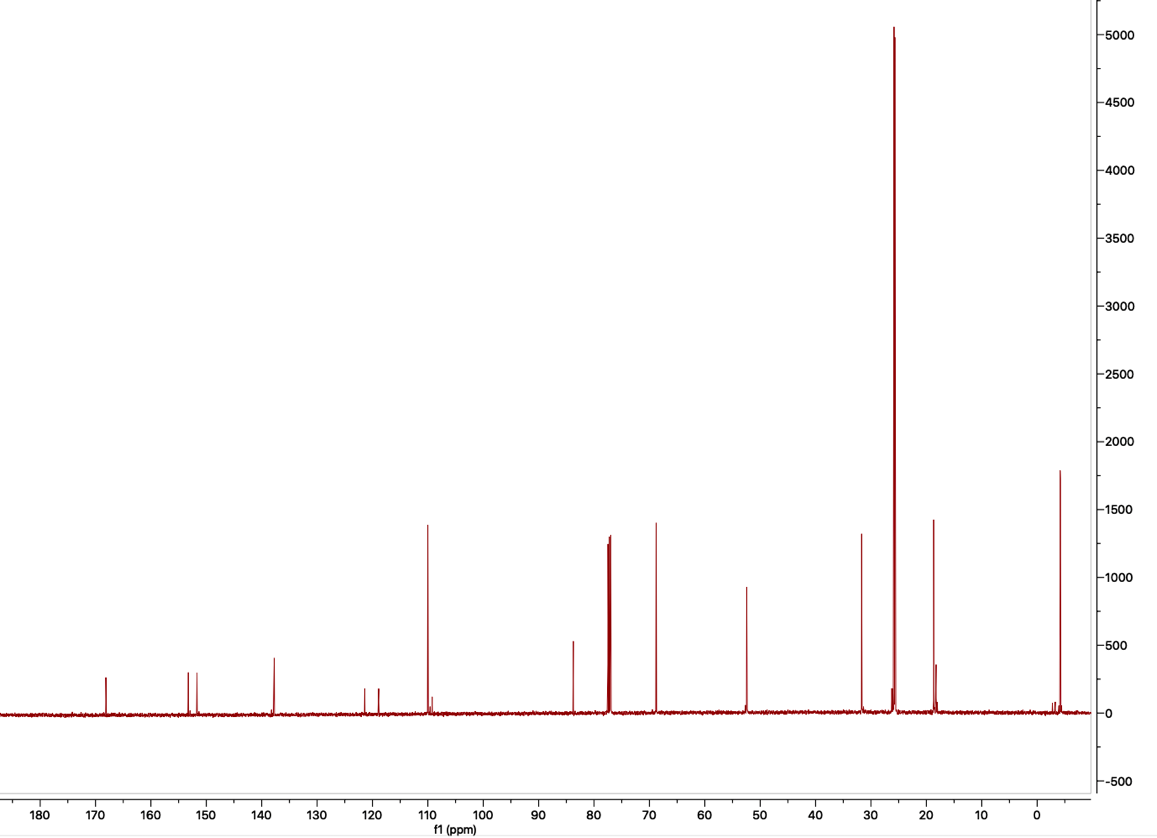
**

**methyl 2-(2-(1-benzyl-1H-1,2,3-triazol-5-yl)ethyl)-3-chloro-4,6-dihydroxybenzoate (10)**

**^1^H NMR**

**
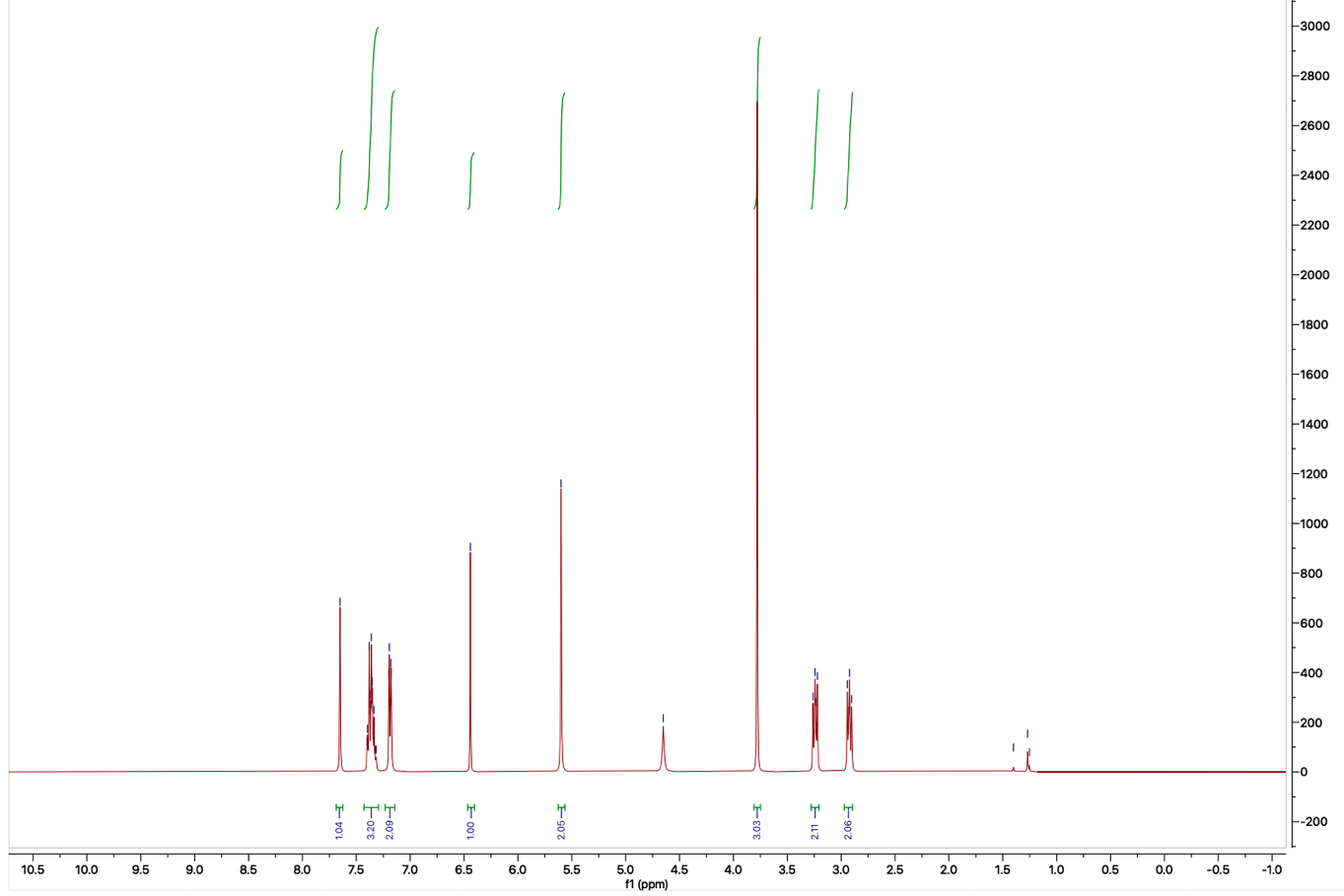
**

**methyl 2-(2-(1-benzyl-1H-1,2,3-triazol-5-yl)ethyl)-3-chloro-4,6-dihydroxybenzoate (10)**

**^13^C NMR**

**
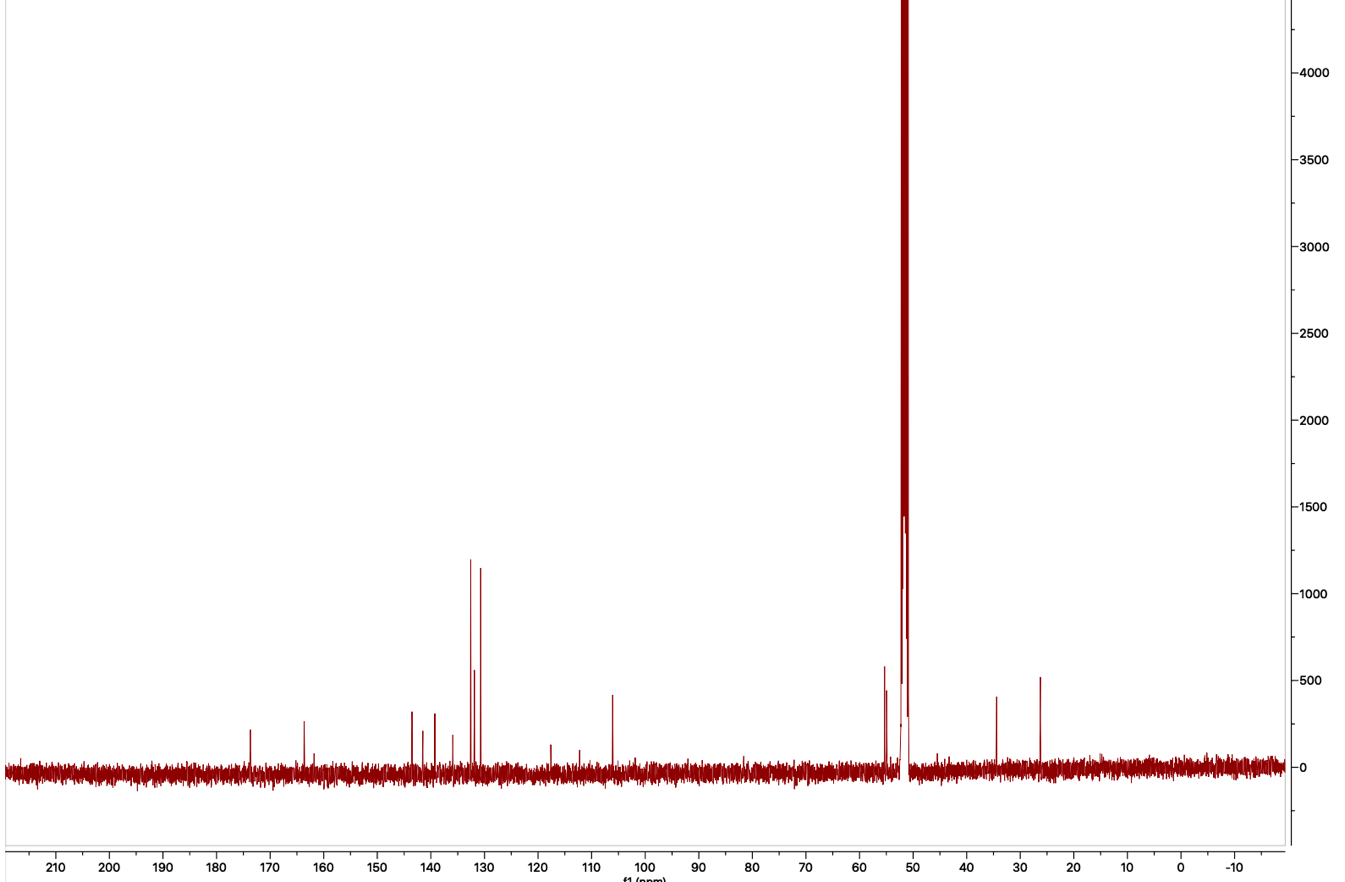
**

**methyl 2-(2-azidoethyl)-4,6-bis((tert-butyldimethylsilyl)oxy)-3-chlorobenzoate (12)**

**^1^H NMR**

**
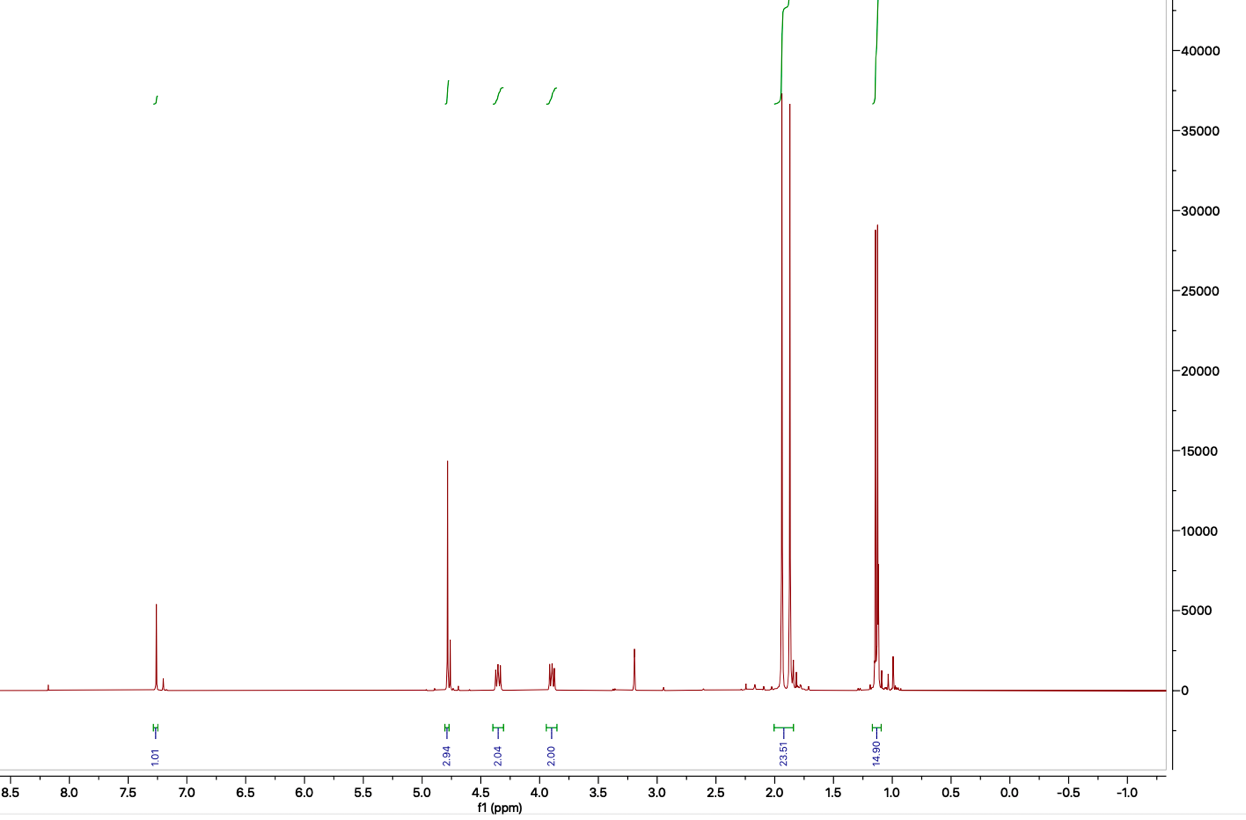
**

**methyl 2-(2-azidoethyl)-4,6-bis((tert-butyldimethylsilyl)oxy)-3-chlorobenzoate (12)**

**^13^C NMR**

**
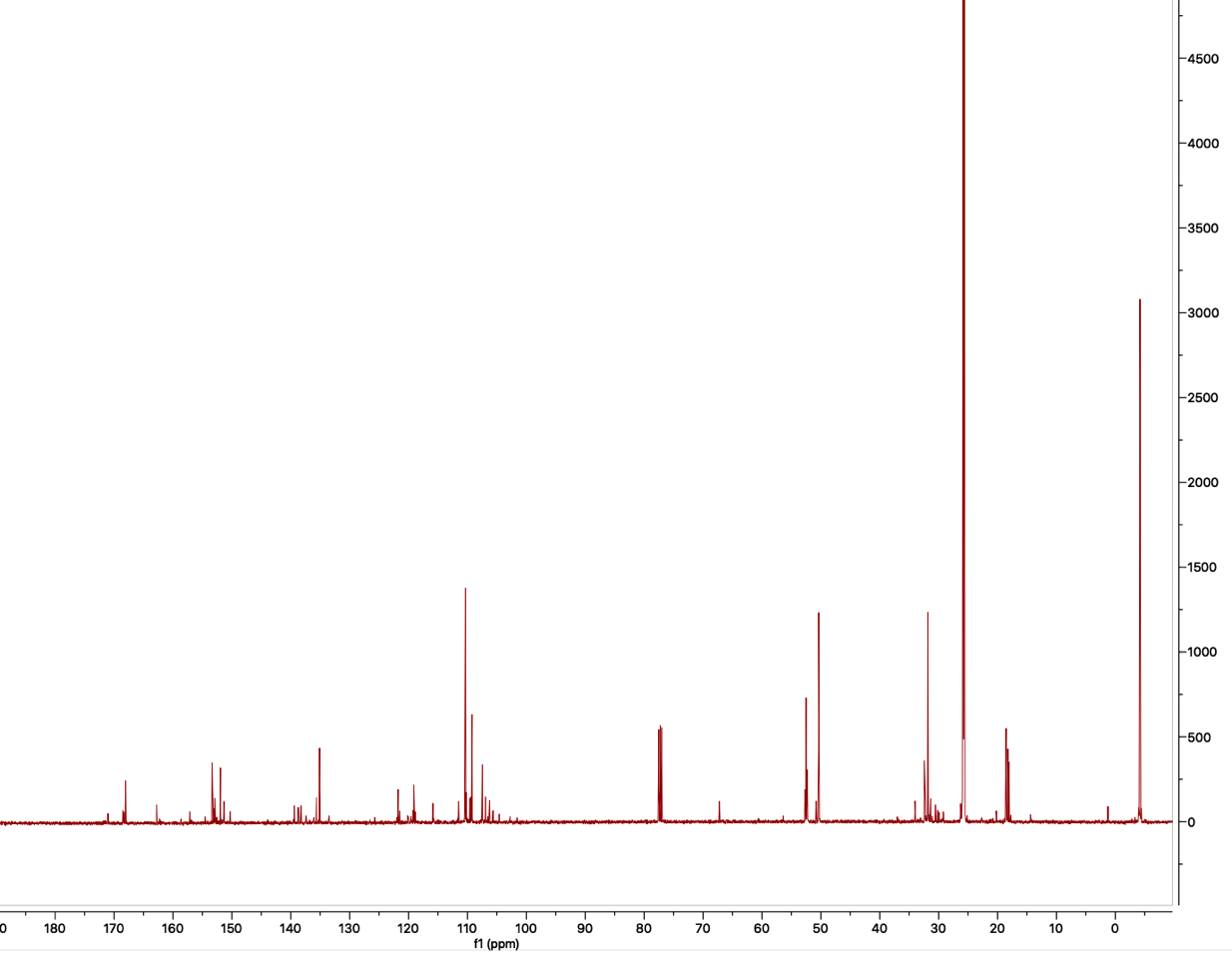
**

**methyl 2-(2-(5-benzyl-1H-1,2,3-triazol-1-yl)ethyl)-3-chloro-4,6-dihydroxybenzoate (14)**

**^1^H NMR**

**
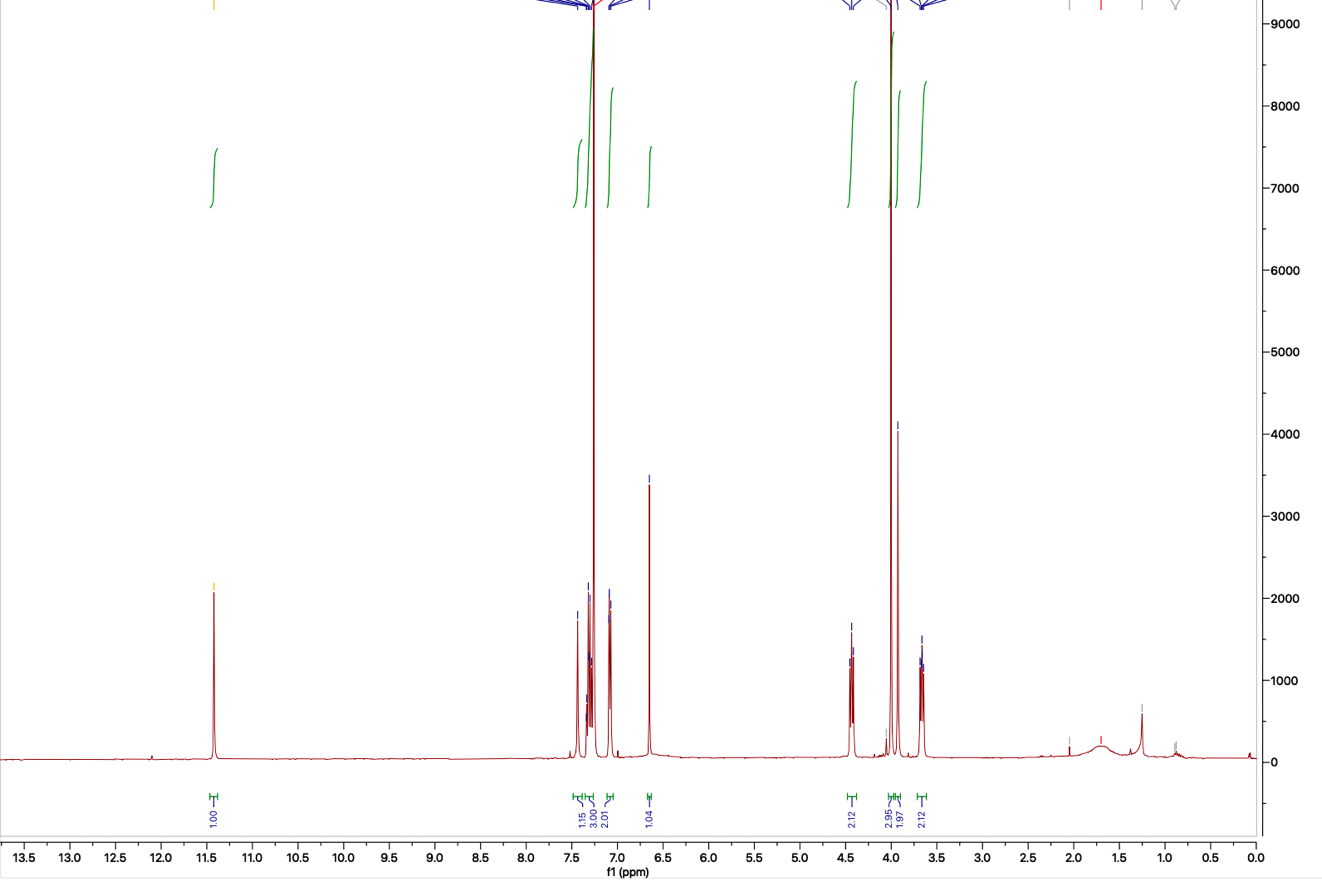
**

**methyl 2-(2-(5-benzyl-1H-1,2,3-triazol-1-yl)ethyl)-3-chloro-4,6-dihydroxybenzoate (14)**

**^13^C NMR**

**
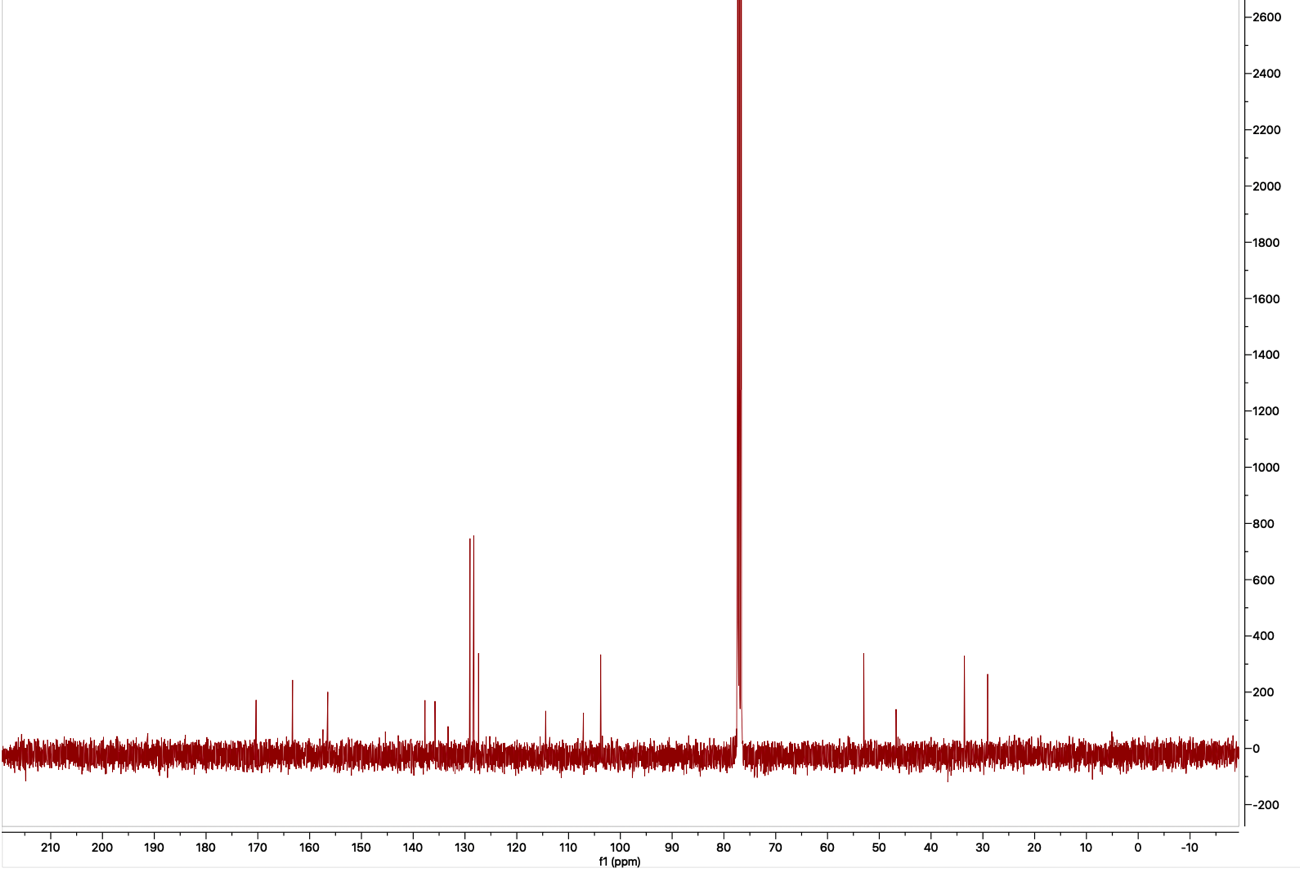
**

**methyl 2-(2-(4-benzyl-1H-1,2,3-triazol-1-yl)ethyl)-3-chloro-4,6-dihydroxybenzoate (16)**

**^1^H NMR**

**
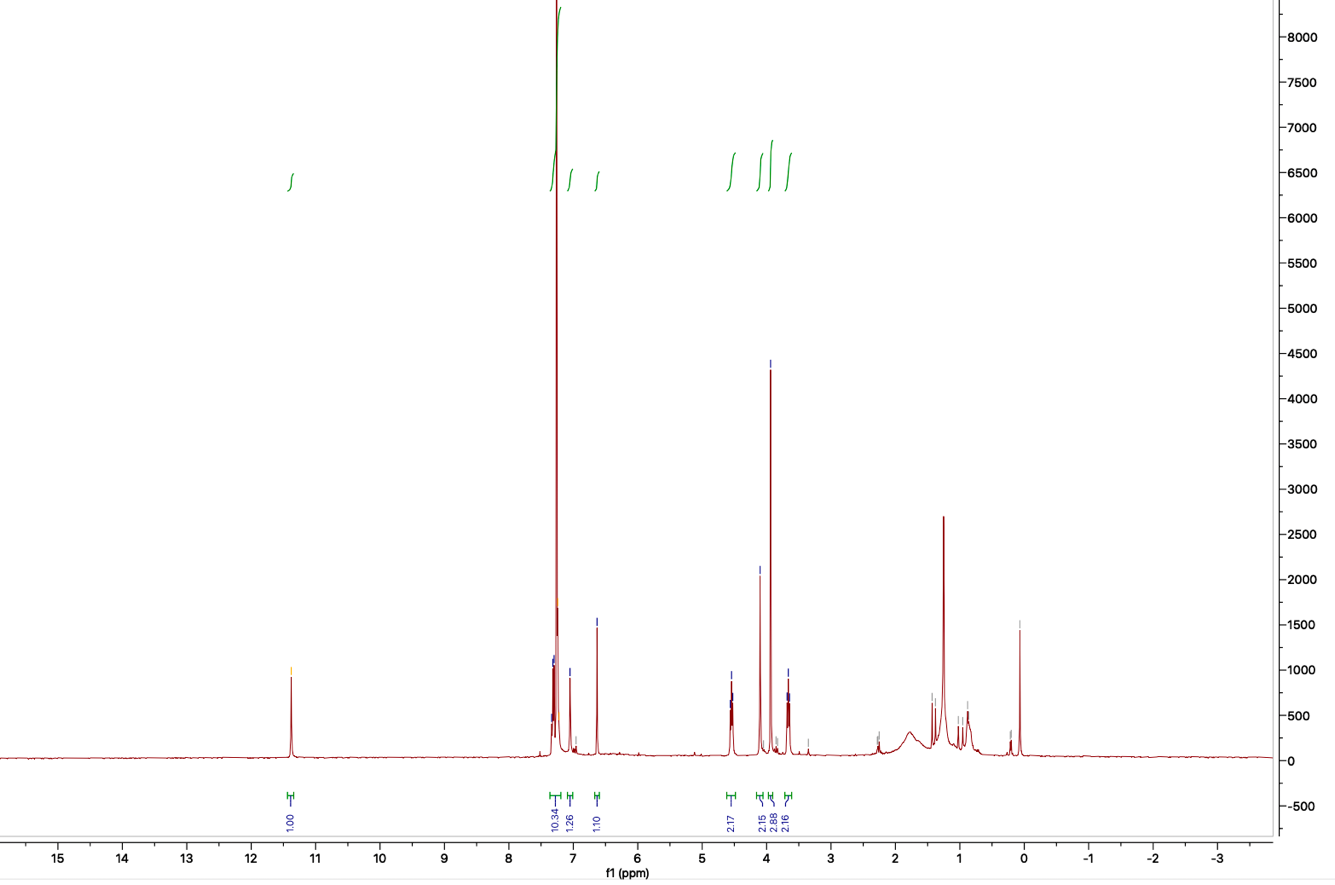
**

**methyl 2-(2-(4-benzyl-1H-1,2,3-triazol-1-yl)ethyl)-3-chloro-4,6-dihydroxybenzoate (16)**

**^13^C NMR**

**
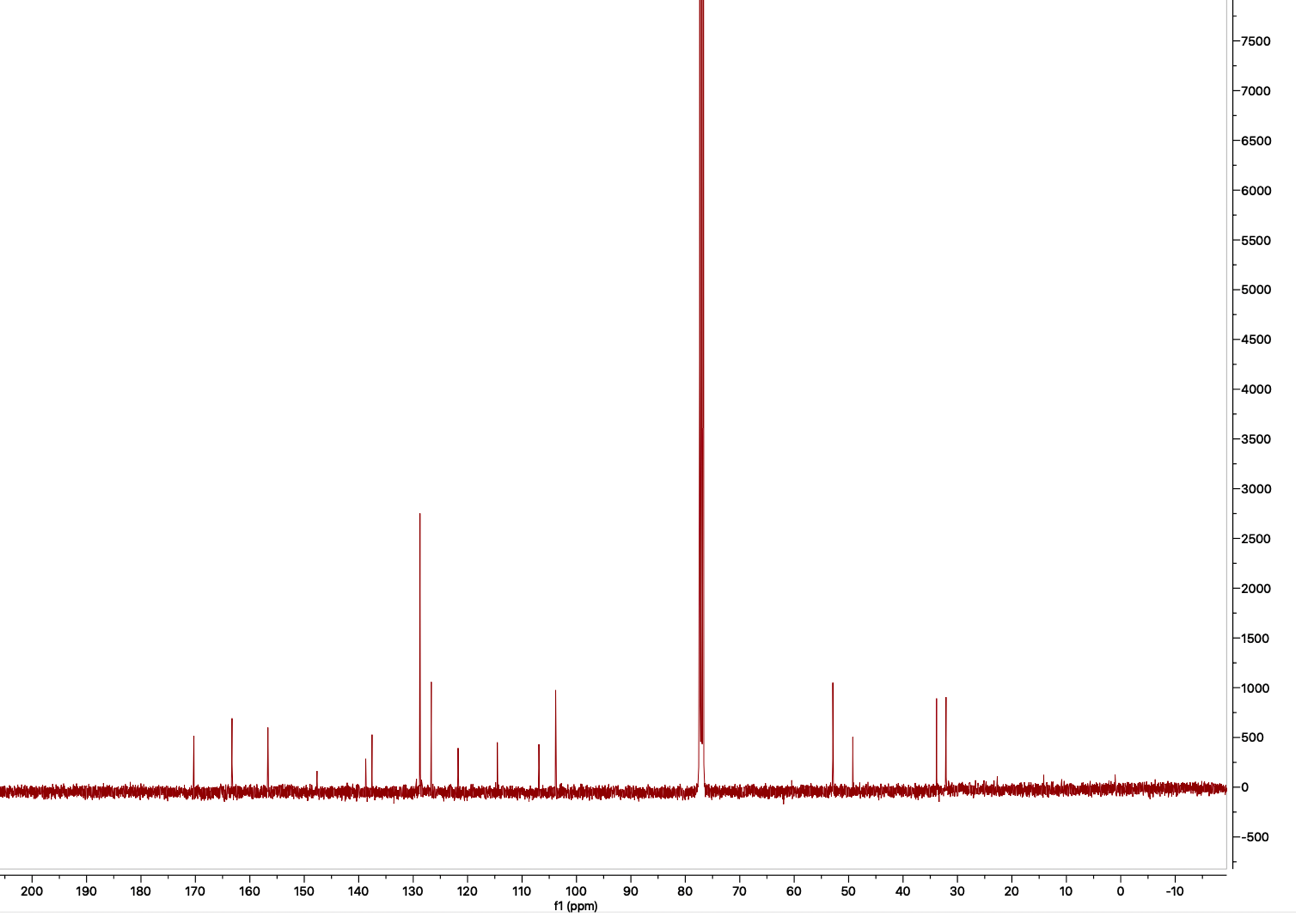
**

**methyl 2-(2-(5-benzyl-1*H*-tetrazol-1-yl)ethyl)-3-chloro-4,6-dihydroxybenzoate (20)**

**^1^H NMR**

**
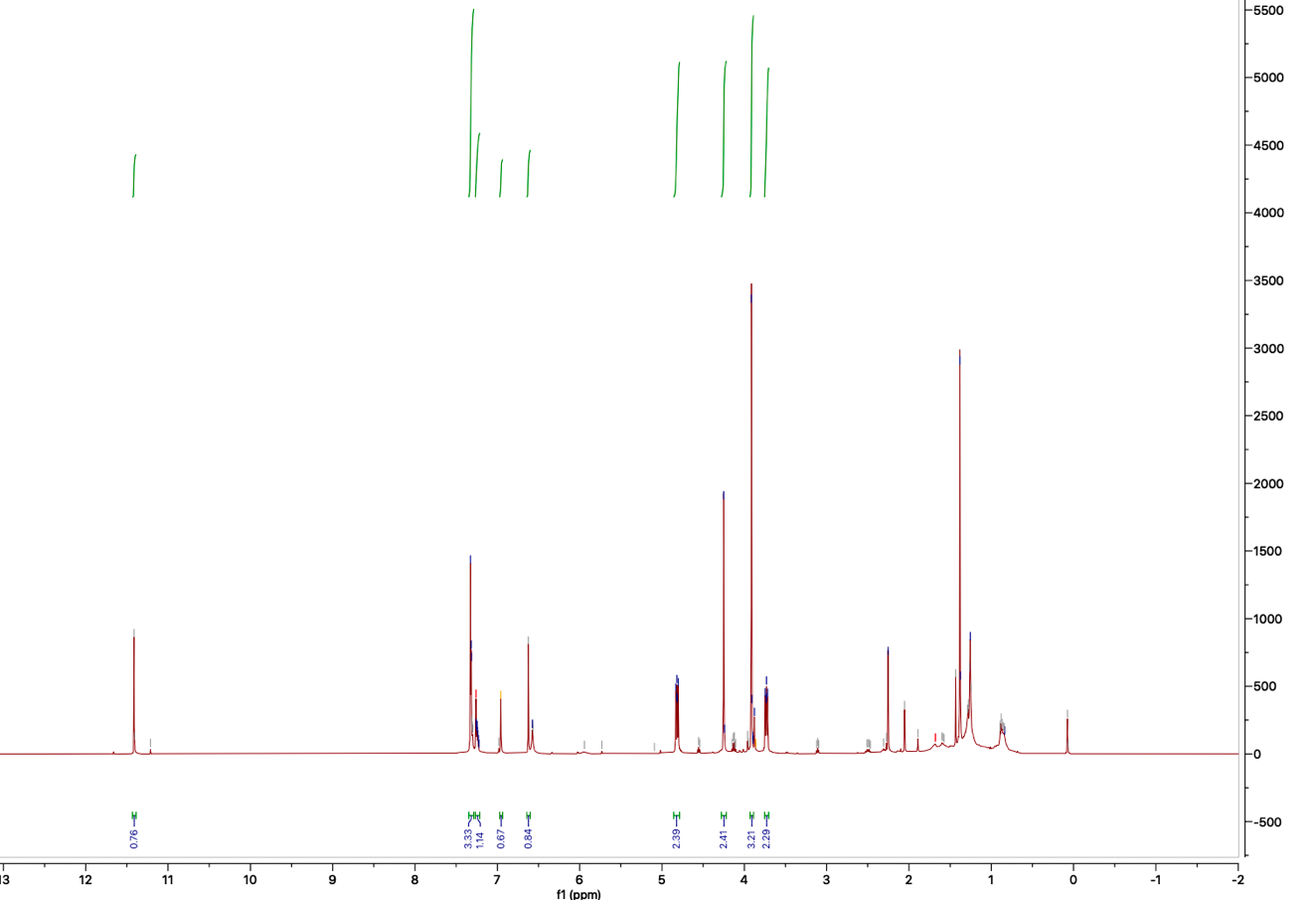
**

**methyl 2-(2-(5-benzyl-1*H*-tetrazol-1-yl)ethyl)-3-chloro-4,6-dihydroxybenzoate (20)**

**^13^C NMR**

**
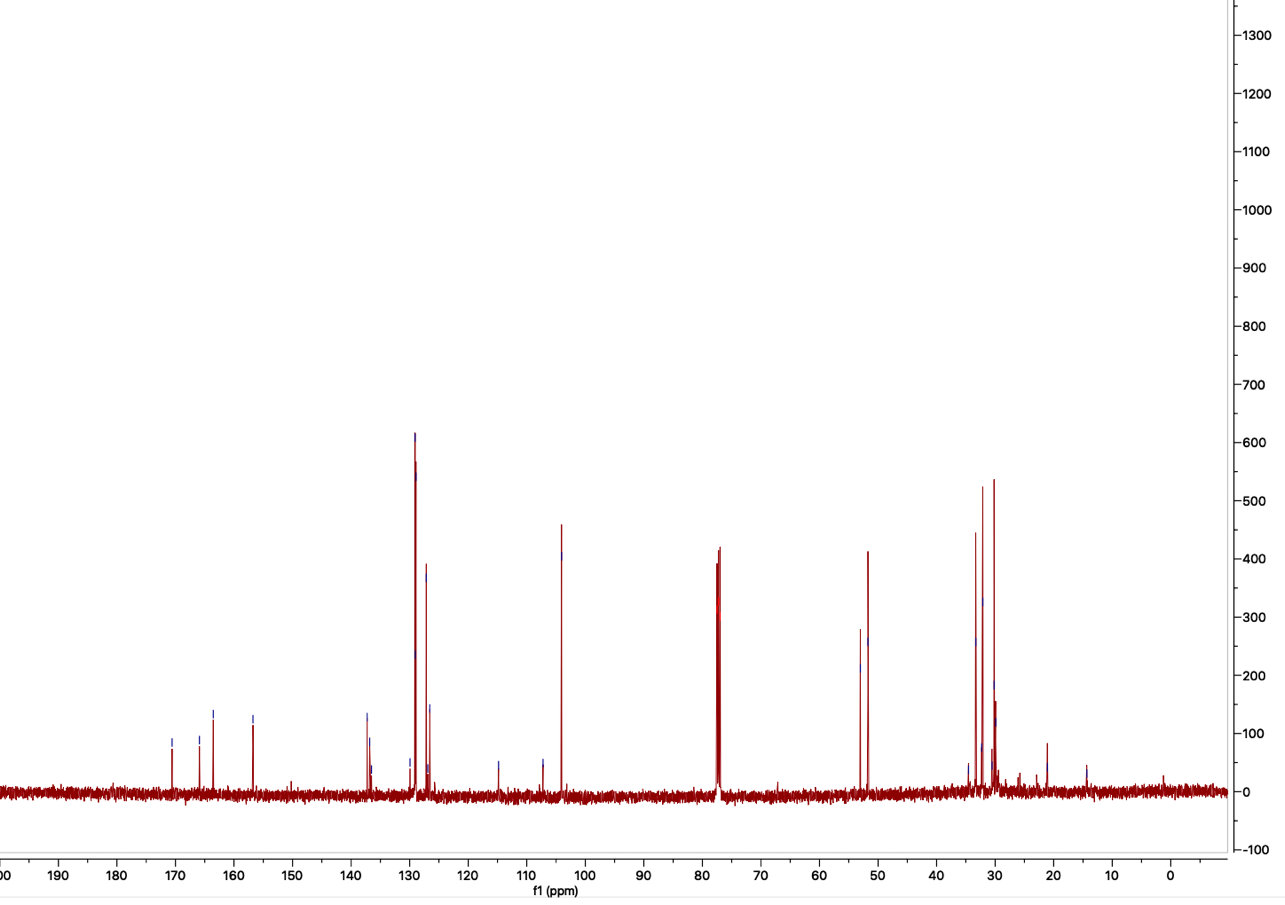
**

**methyl 3-chloro-2-(2-(5-(4-fluorobenzyl)-1H-1,2,3-triazol-1-yl)ethyl)-4,6-dihydroxybenzoate (31)**

**^1^H NMR**

**
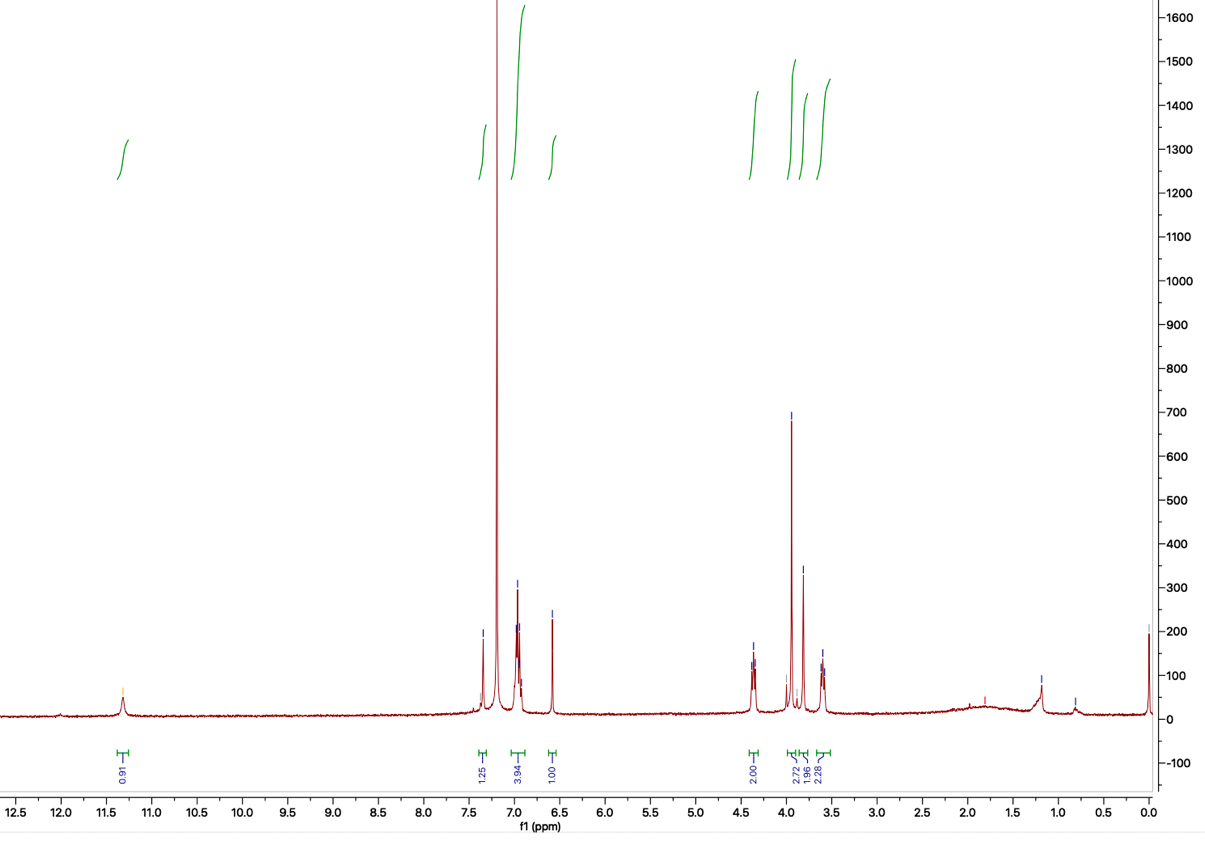
**

**methyl 3-chloro-2-(2-(5-(4-fluorobenzyl)-1H-1,2,3-triazol-1-yl)ethyl)-4,6-dihydroxybenzoate (31)**

**^13^C NMR**

**
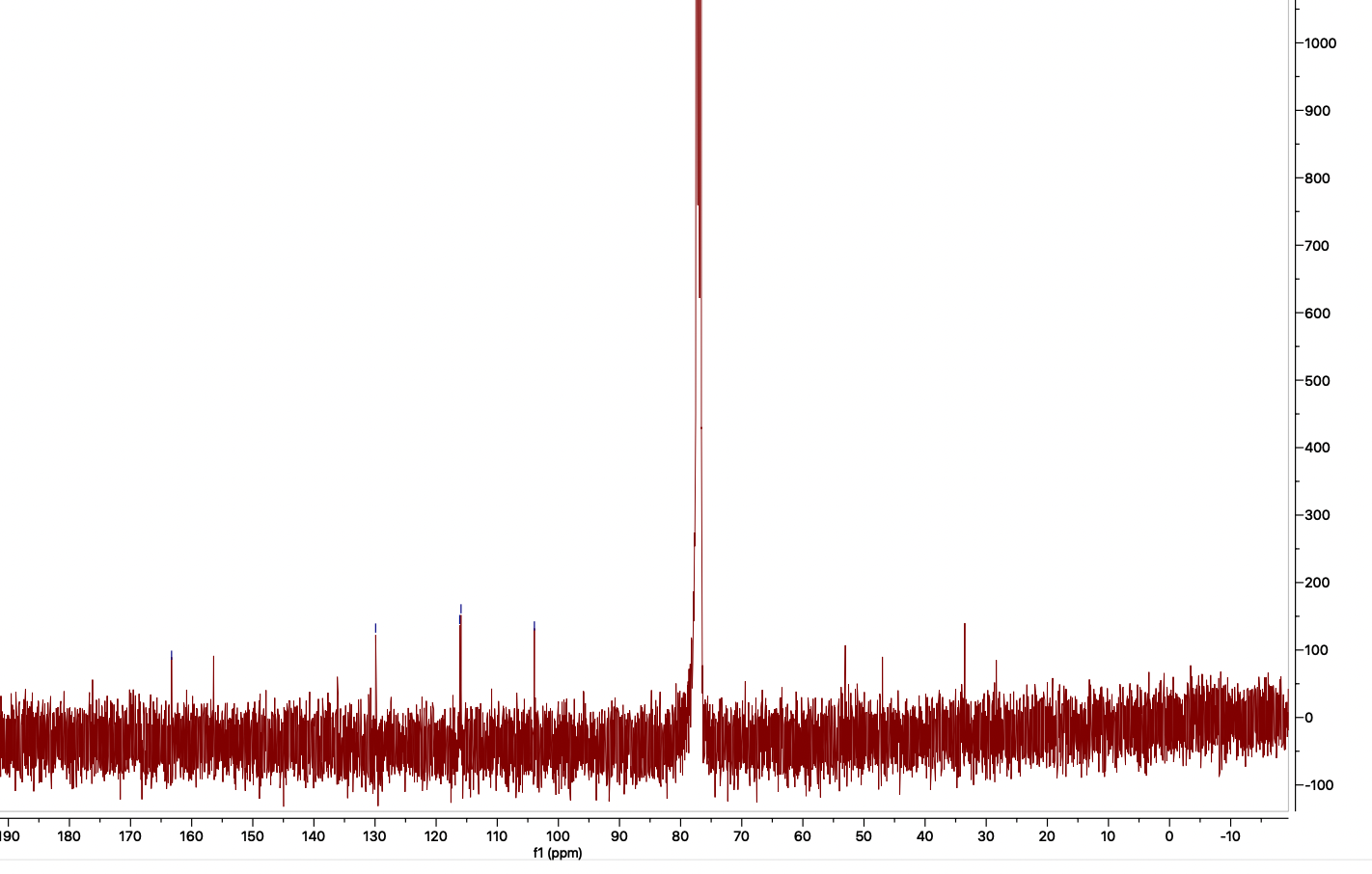
**

**methyl 3-chloro-2-(2-(5-(4-chlorobenzyl)-1H-1,2,3-triazol-1-yl)ethyl)-4,6-dihydroxybenzoate (32)**

**^1^H NMR**

**
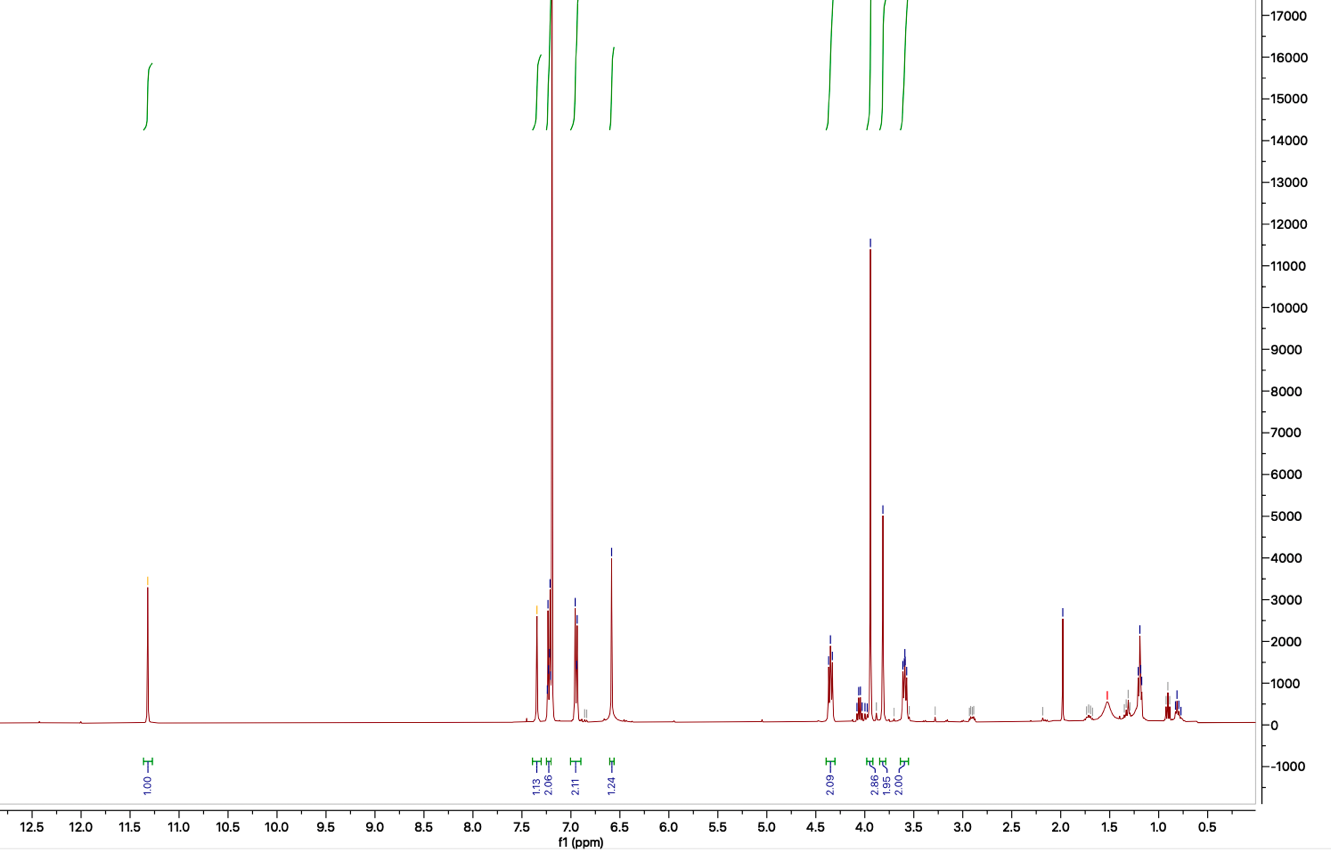
**

**methyl 3-chloro-2-(2-(5-(4-chlorobenzyl)-1H-1,2,3-triazol-1-yl)ethyl)-4,6-dihydroxybenzoate (32)**

**^13^C NMR**

**
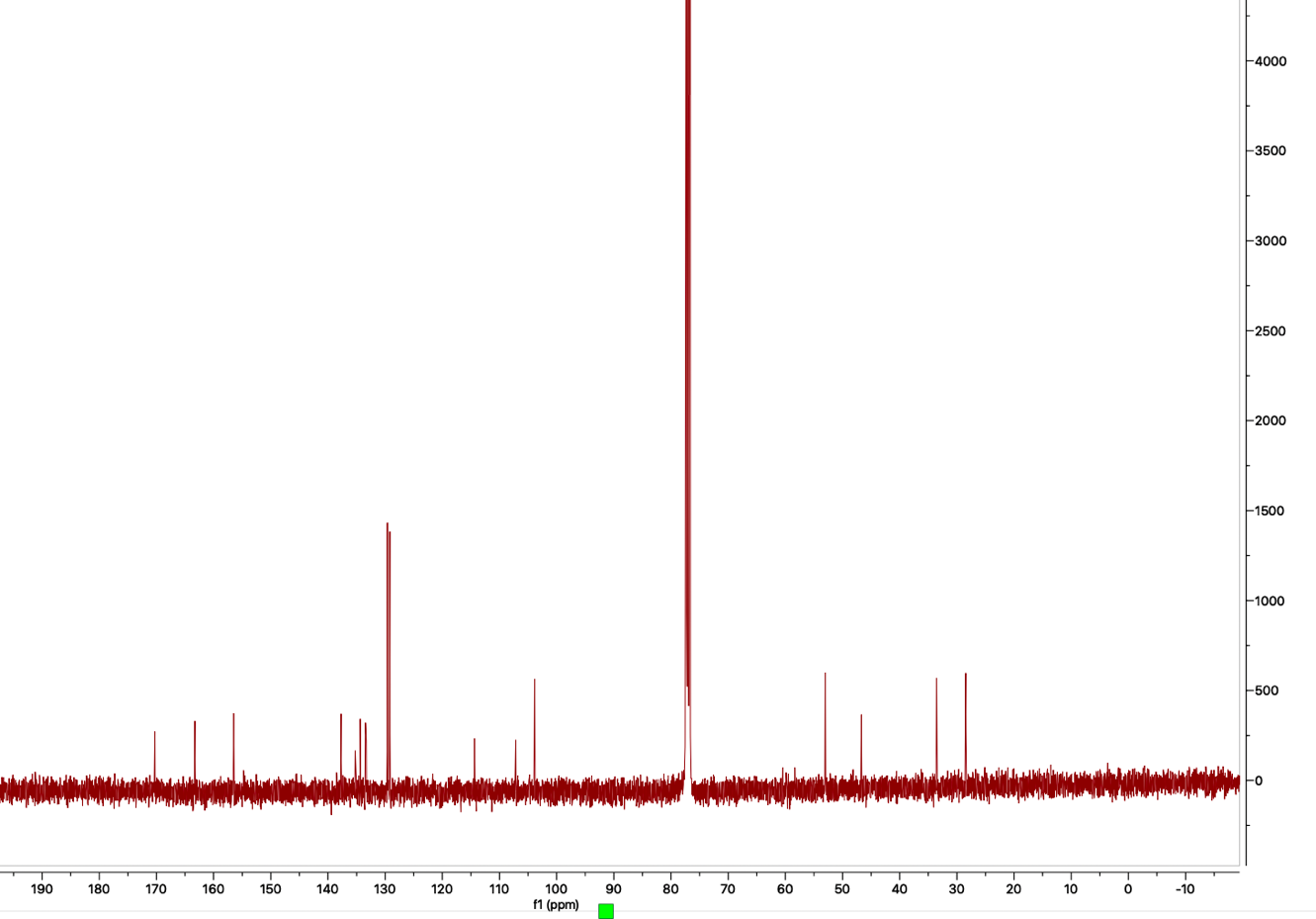
**

**methyl 2-(2-(5-(4-bromobenzyl)-1*H*-1,2,3-triazol-1-yl)ethyl)-3-chloro-4,6-dihydroxybenzoate (33)**

**^1^H NMR**

**
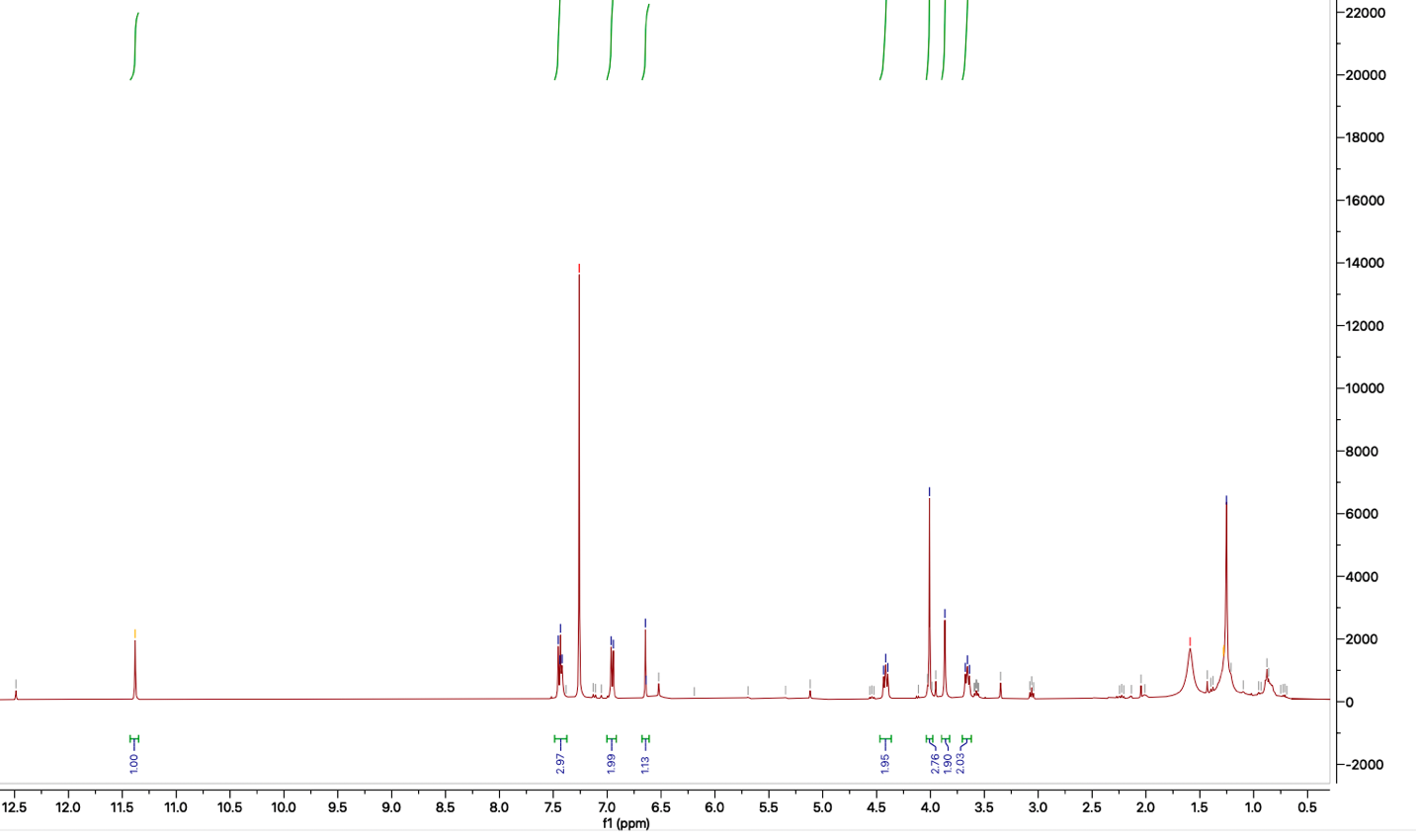
**

**methyl 2-(2-(5-(4-bromobenzyl)-1*H*-1,2,3-triazol-1-yl)ethyl)-3-chloro-4,6-dihydroxybenzoate (33)**

**^13^C NMR**

**
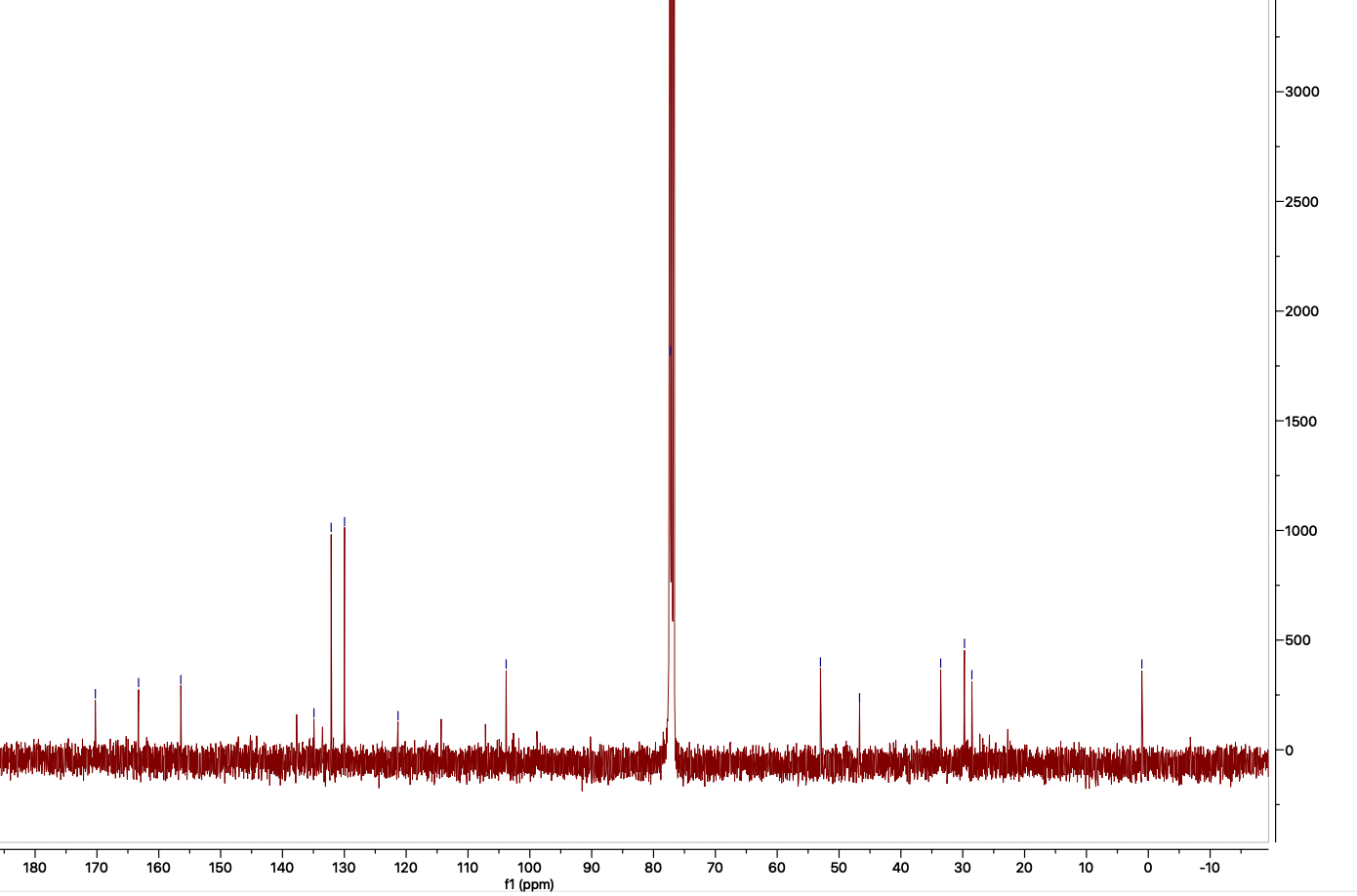
**

**methyl 3-chloro-4,6-dihydroxy-2-(2-(5-(4-methylbenzyl)-1H-1,2,3-triazol-1-yl)ethyl)benzoate (34)**

**^1^H NMR**

**
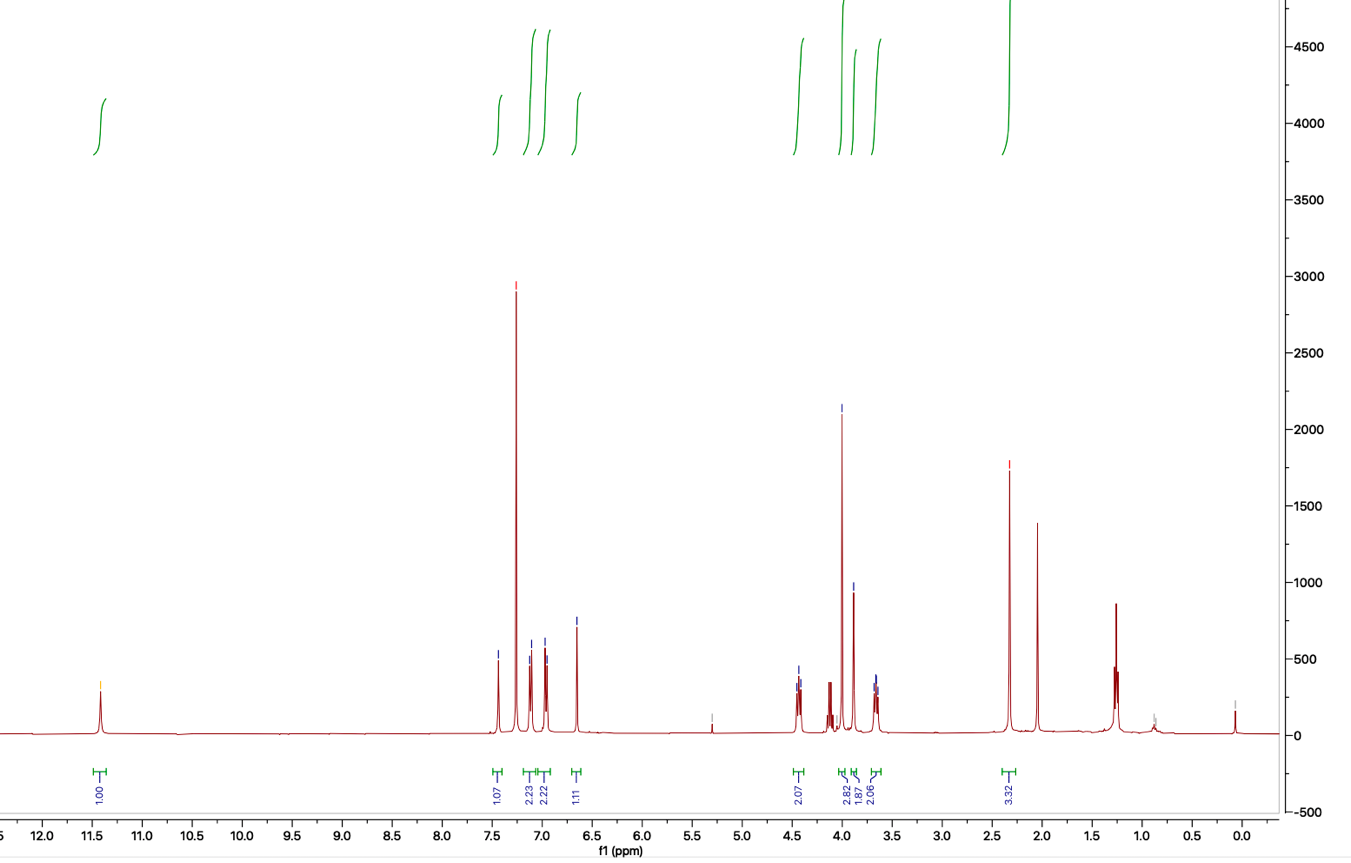
**

**methyl 3-chloro-4,6-dihydroxy-2-(2-(5-(4-methylbenzyl)-1H-1,2,3-triazol-1-yl)ethyl)benzoate (34)**

**^13^C NMR**

**
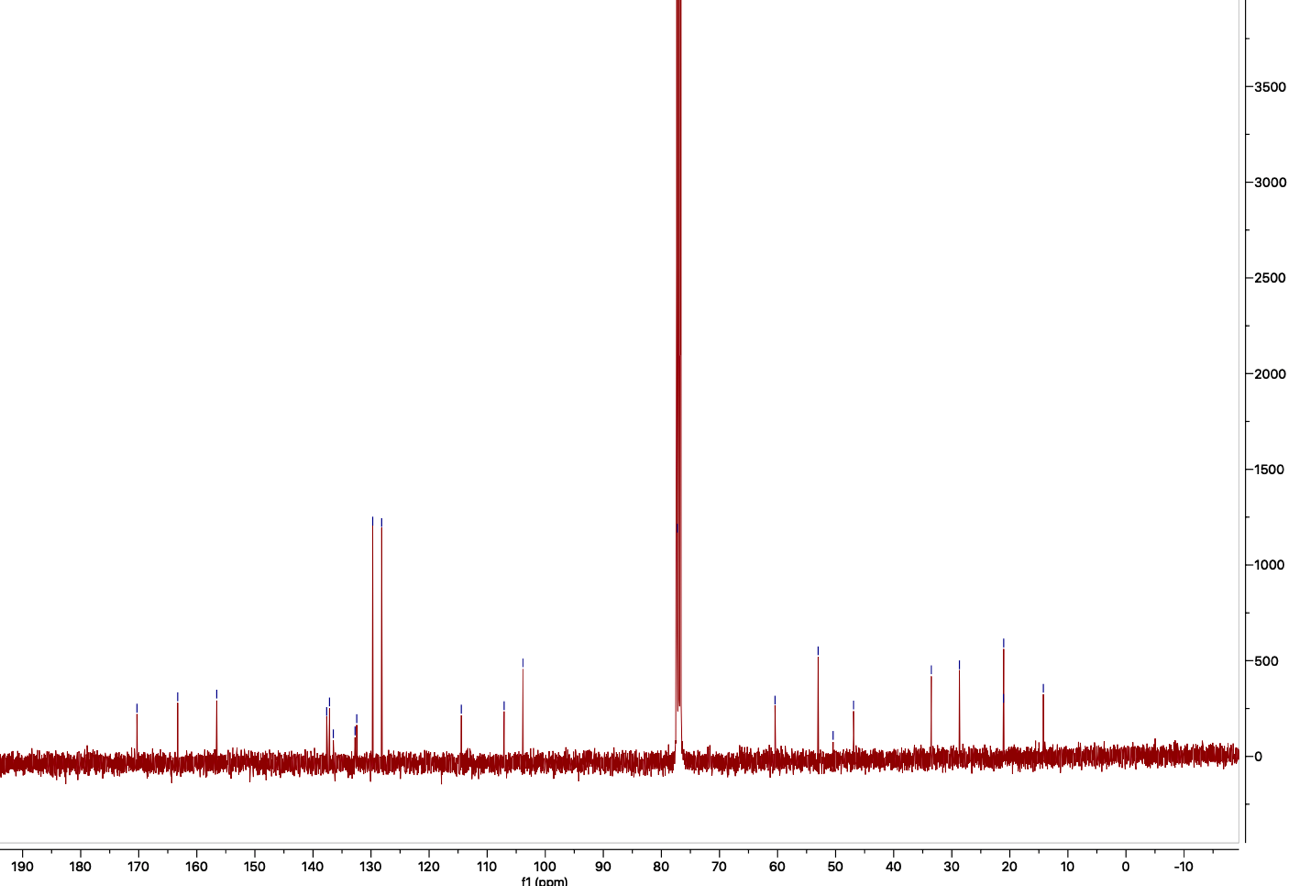
**

**methyl 3-chloro-2-(2-(5-(4-ethylbenzyl)-1H-1,2,3-triazol-1-yl)ethyl)-4,6-dihydroxybenzoate (35)**

**^1^H NMR**

**
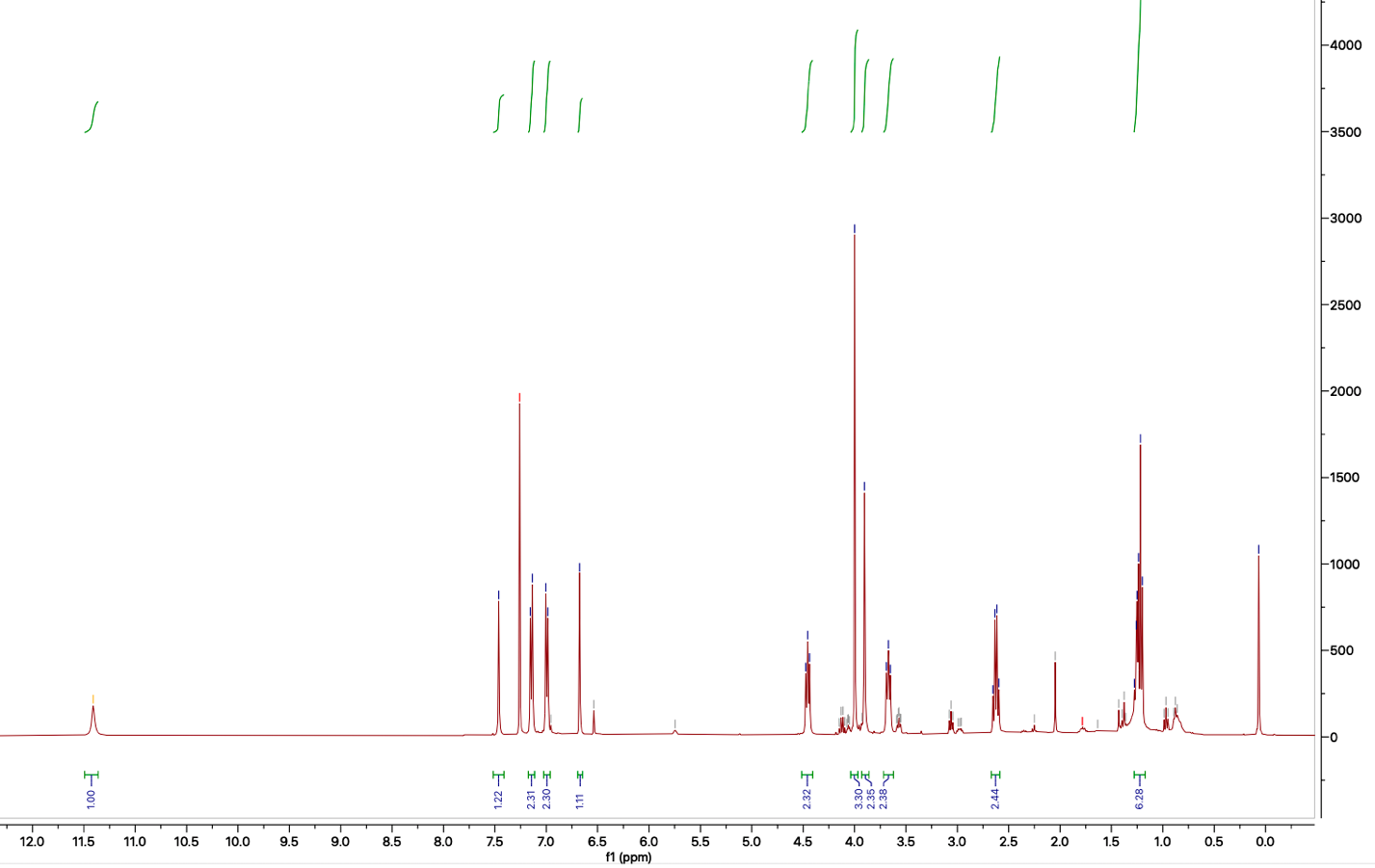
**

**methyl 3-chloro-2-(2-(5-(4-ethylbenzyl)-1H-1,2,3-triazol-1-yl)ethyl)-4,6-dihydroxybenzoate (35)**

**^13^C NMR**

**
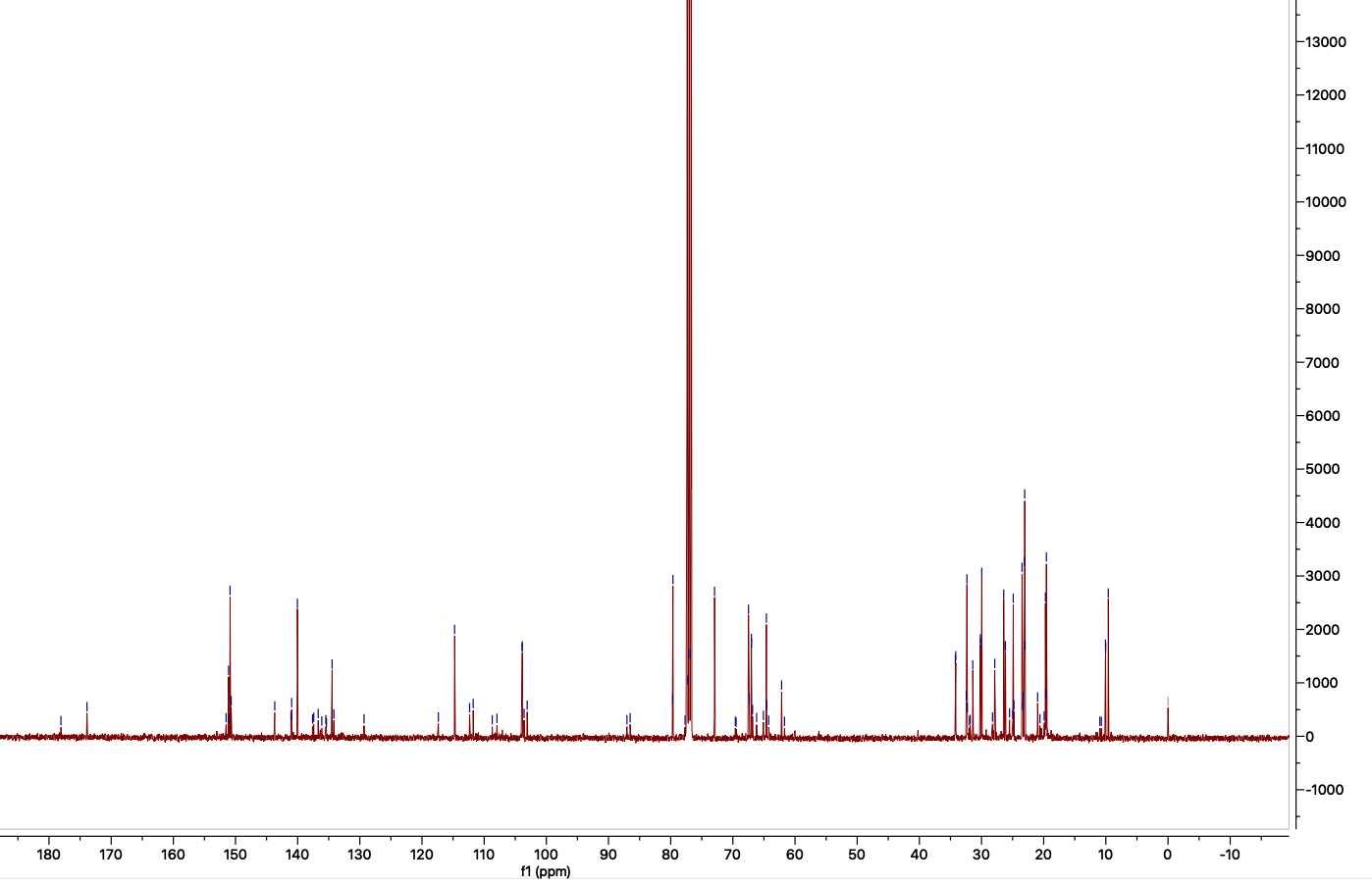
**

**methyl 3-chloro-4,6-dihydroxy-2-(2-(5-(4-methoxybenzyl)-1H-1,2,3-triazol-1-yl)ethyl)benzoate (36)**

**^1^H NMR**

**
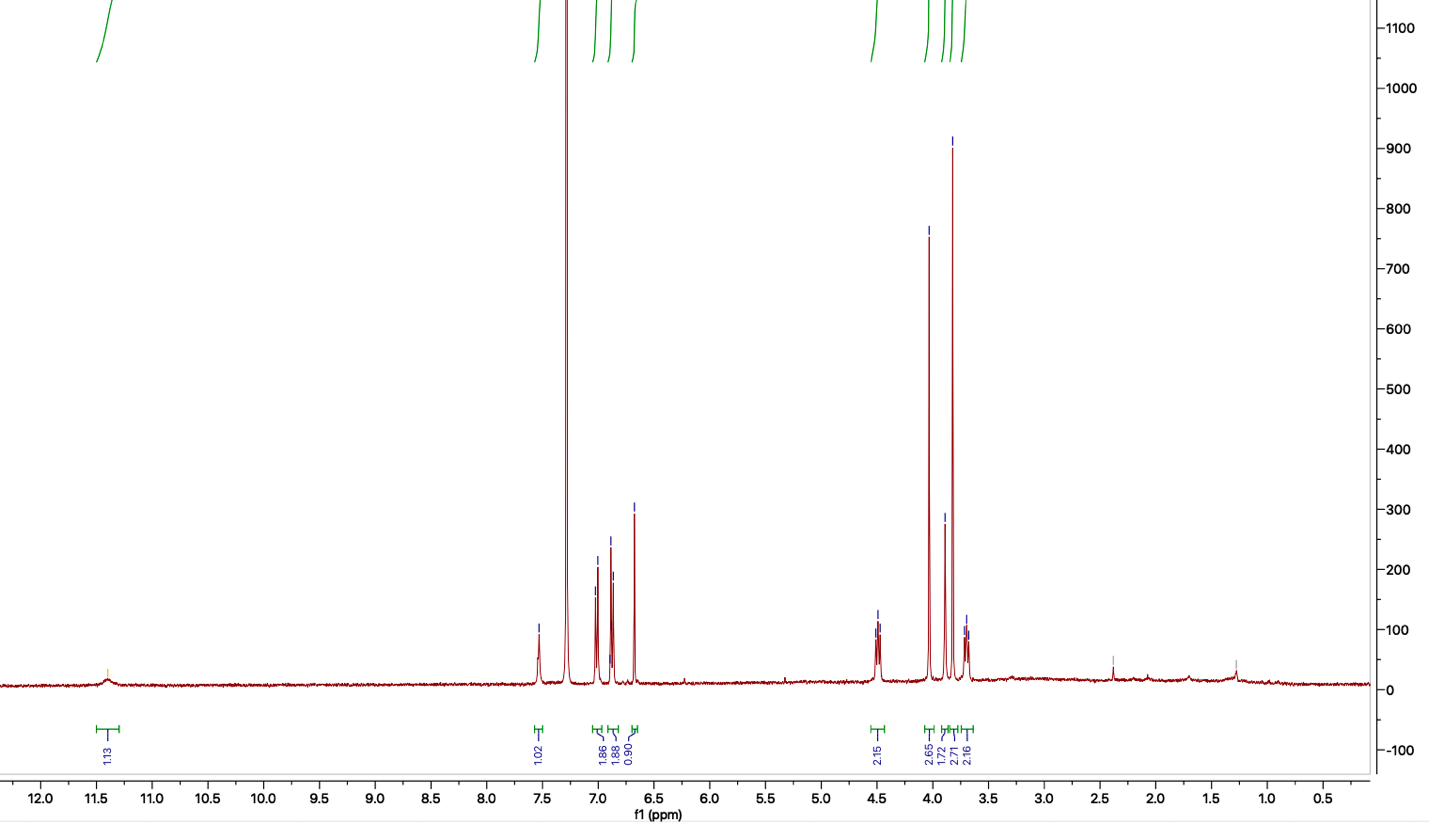
**

**methyl 3-chloro-4,6-dihydroxy-2-(2-(5-(4-methoxybenzyl)-1H-1,2,3-triazol-1-yl)ethyl)benzoate (36)**

**^13^C NMR**

**
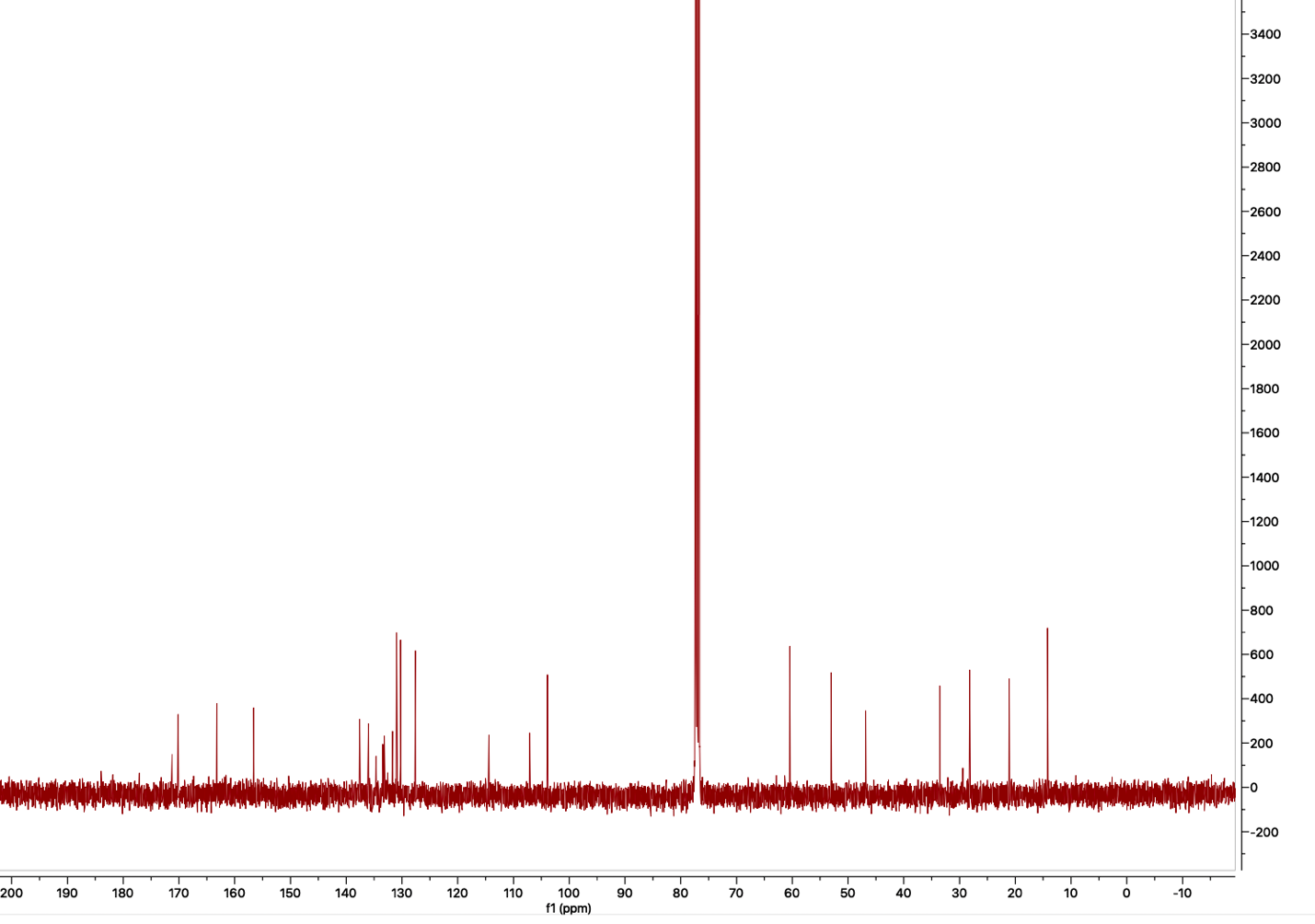
**

**methyl 3-chloro-2-(2-(5-(3,4-dichlorobenzyl)-1H-1,2,3-triazol-1-yl)ethyl)-4,6-dihydroxybenzoate (37)**

**^1^H NMR**

**
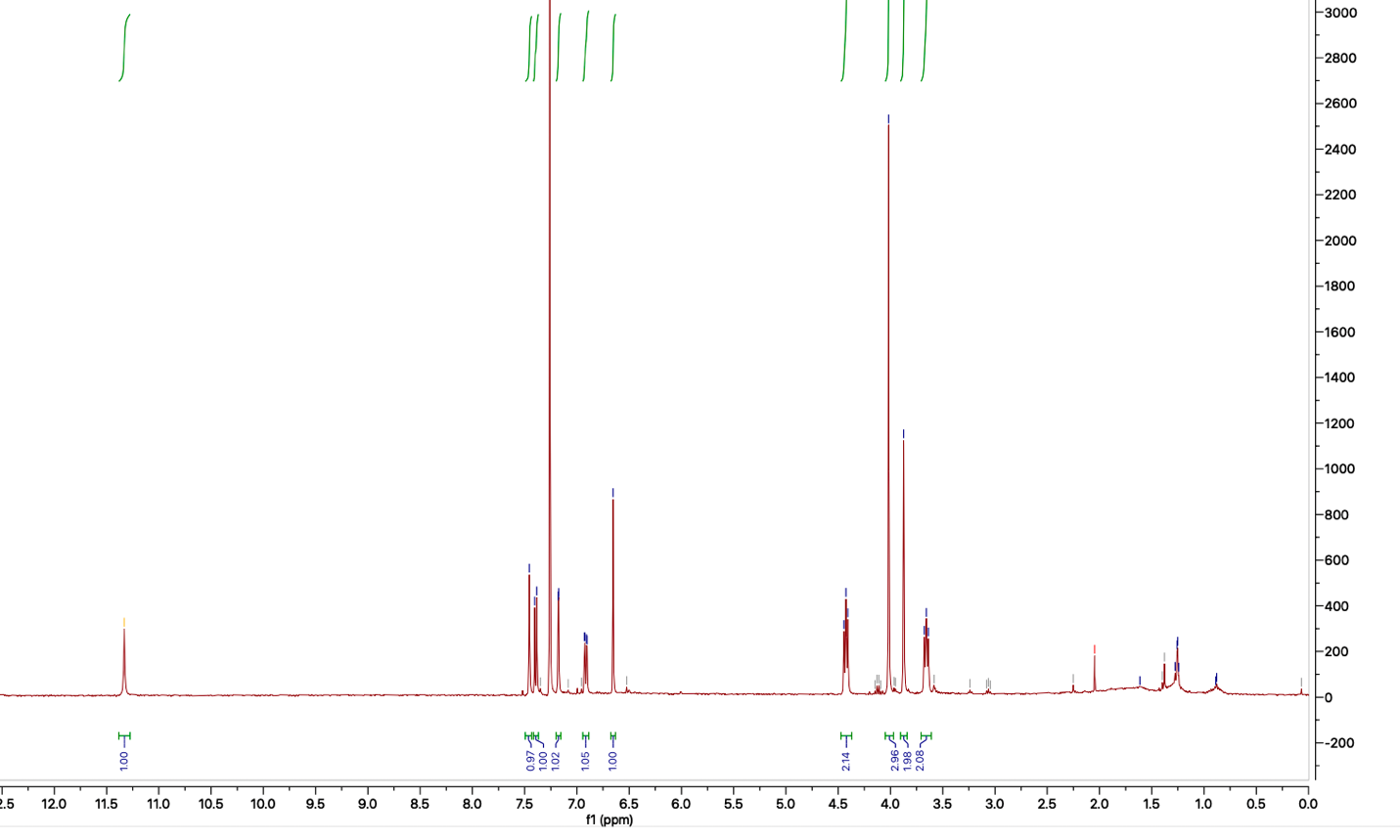
**

**methyl 3-chloro-2-(2-(5-(3,4-dichlorobenzyl)-1H-1,2,3-triazol-1-yl)ethyl)-4,6-dihydroxybenzoate (37)**

**^13^C NMR**

**
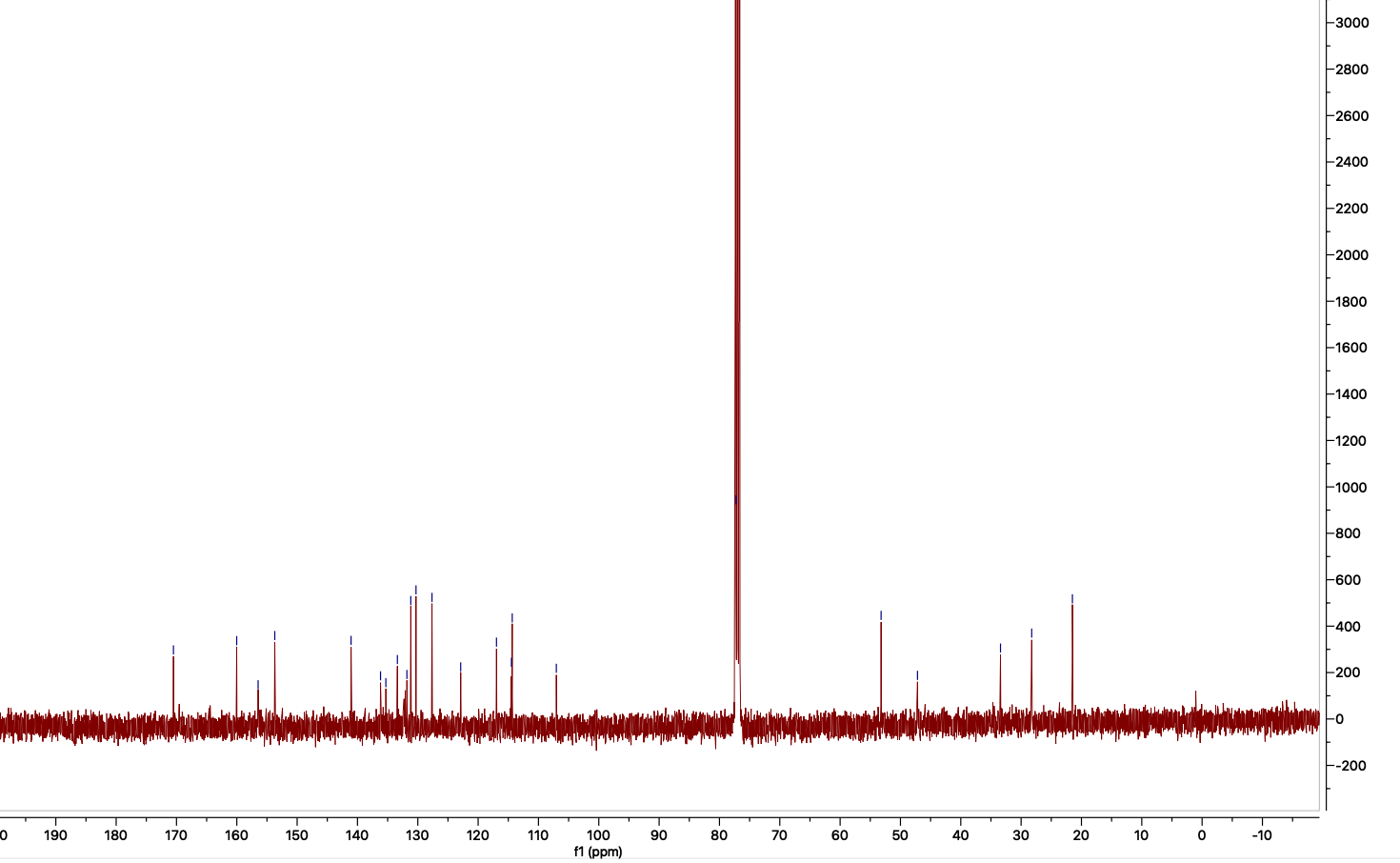
**

**methyl 3-chloro-2-(2-(5-(2-ethoxybenzyl)-1H-1,2,3-triazol-1-yl)ethyl)-4,6-dihydroxybenzoate (40)**

**^1^H NMR**

**
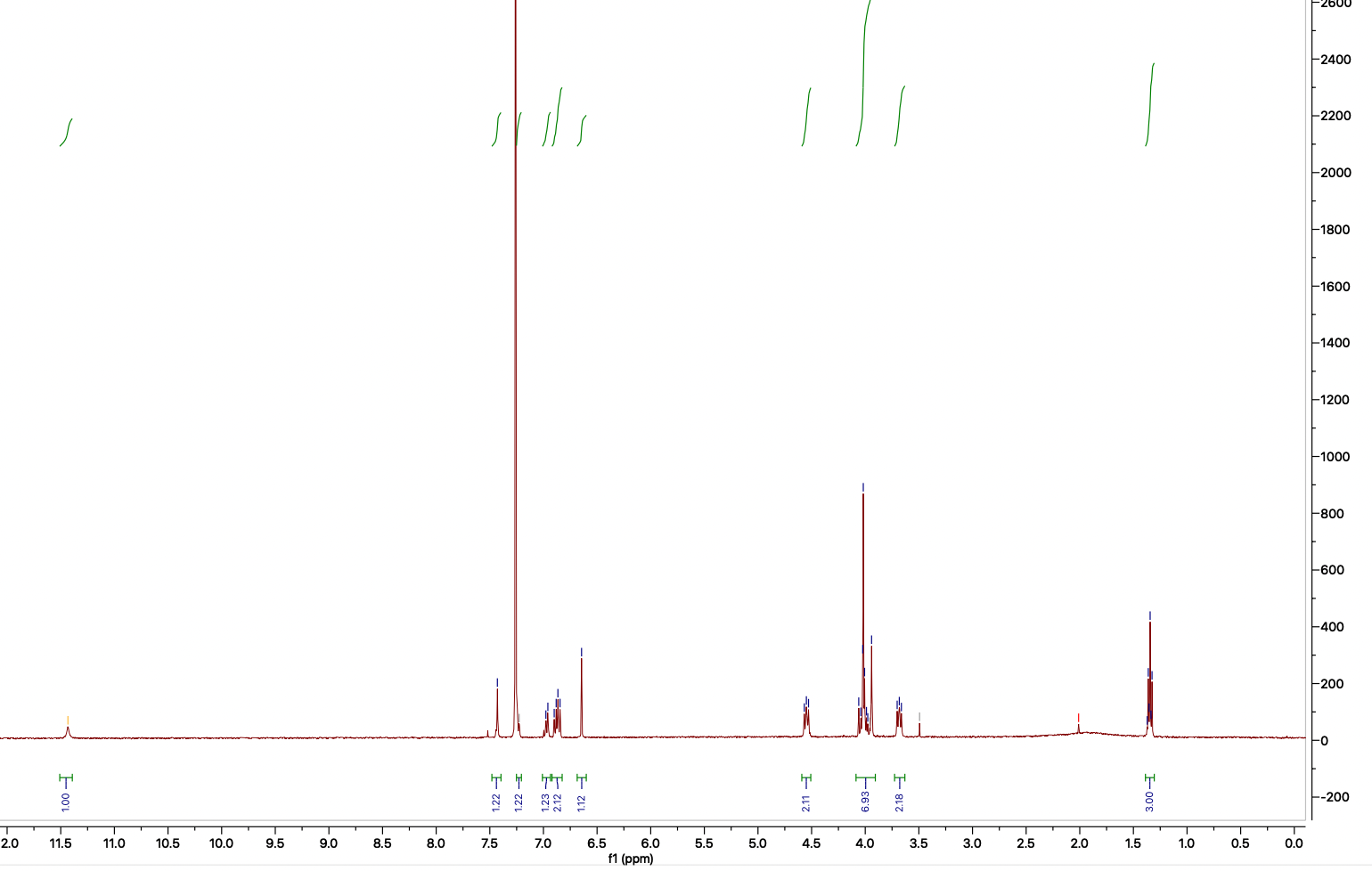
**

**methyl 3-chloro-2-(2-(5-(2-ethoxybenzyl)-1H-1,2,3-triazol-1-yl)ethyl)-4,6-dihydroxybenzoate (40)**

**^13^C NMR**

**
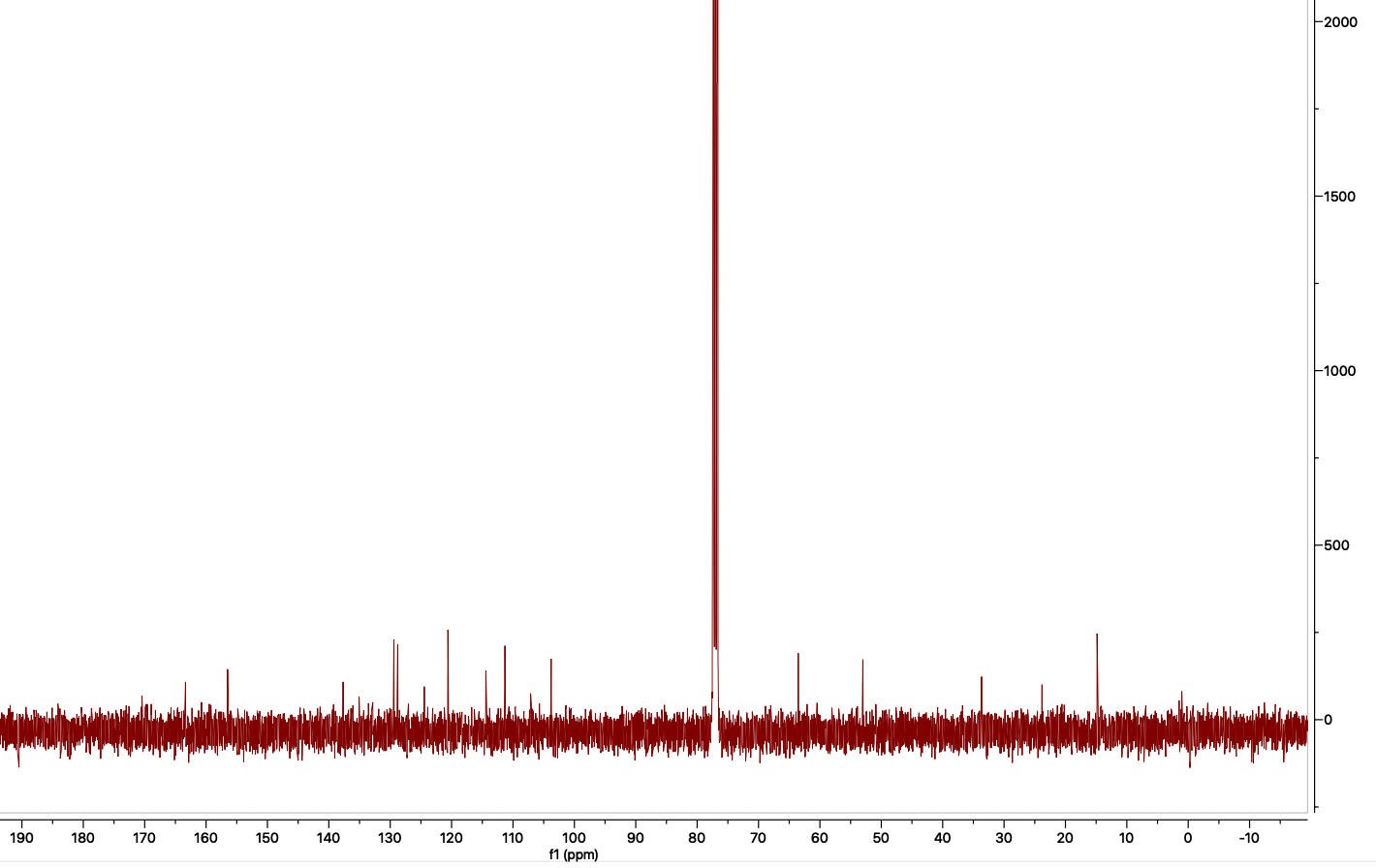
**

**methyl 3-chloro-2-(2-(5-(4-chloro-2-ethoxybenzyl)-1H-1,2,3-triazol-1-yl)ethyl)-4,6-dihydroxybenzoate (41)**

**^1^H NMR**

**
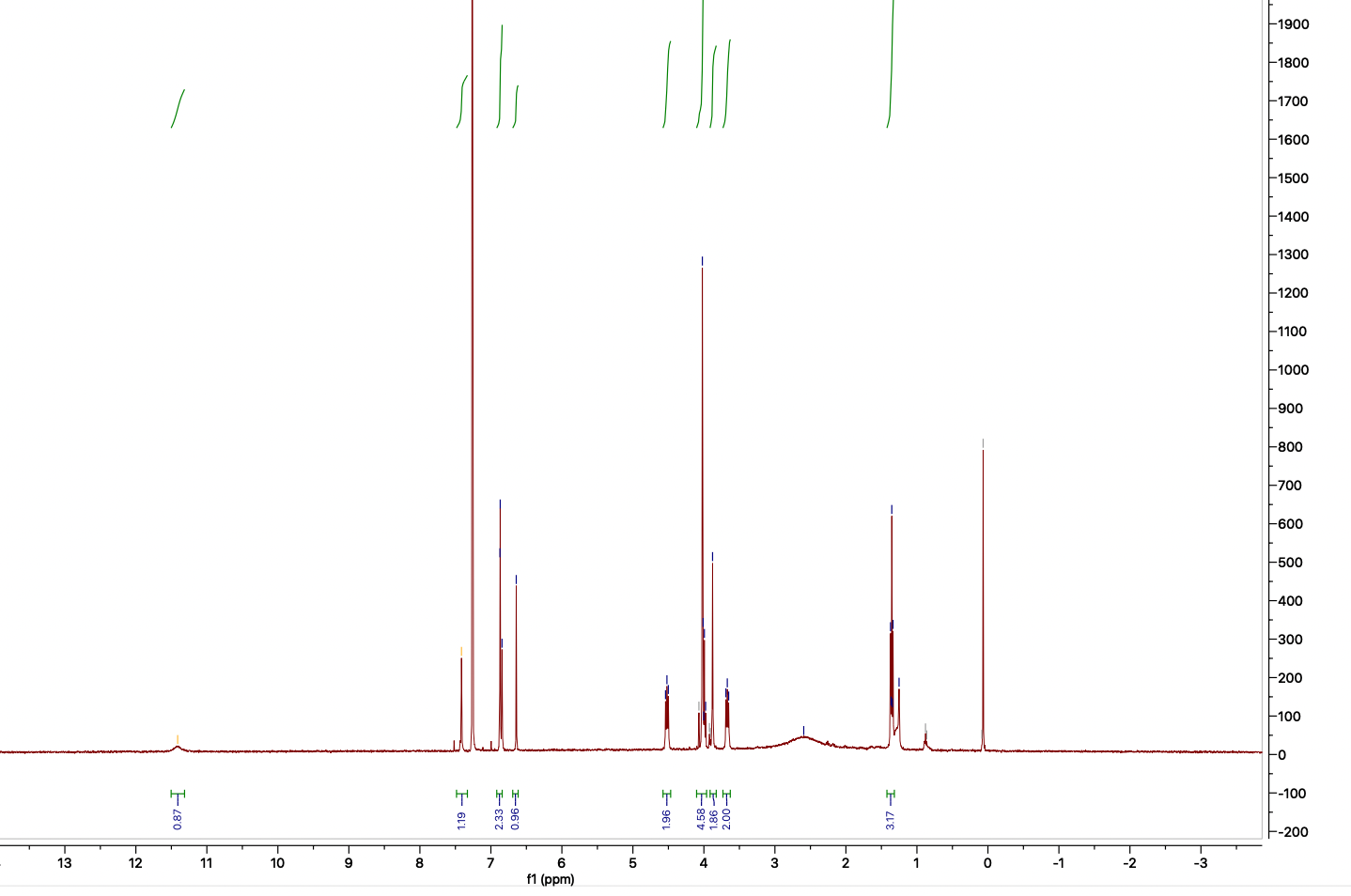
**

**methyl 3-chloro-2-(2-(5-(4-chloro-2-ethoxybenzyl)-1H-1,2,3-triazol-1-yl)ethyl)-4,6-dihydroxybenzoate (41)**

**^13^C NMR**

**
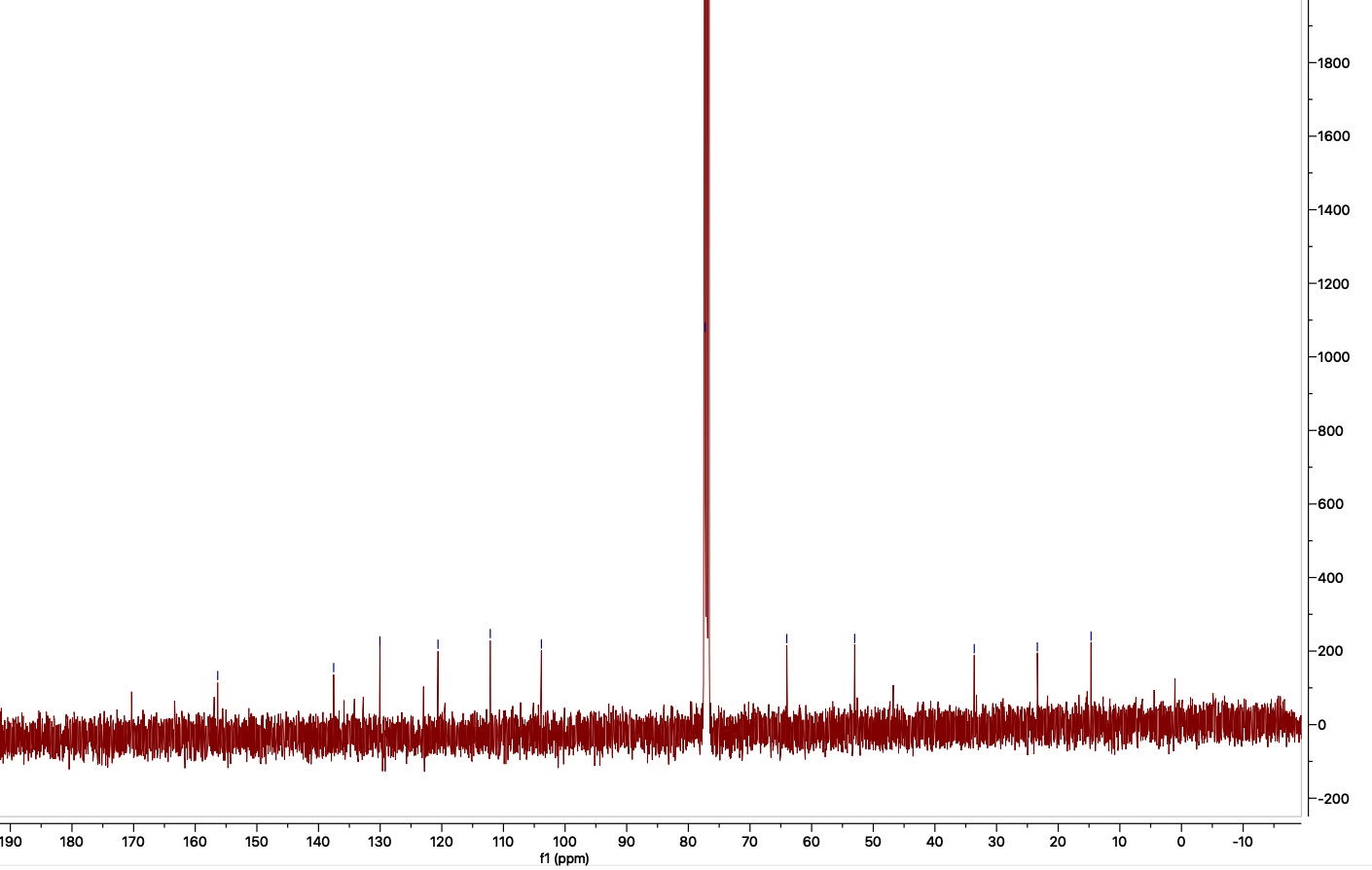
**

**methyl 3-chloro-4,6-dihydroxy-2-(2-(5-(2-propoxybenzyl)-1H-1,2,3-triazol-1-yl)ethyl)benzoate (42)**

**^1^H NMR**

**
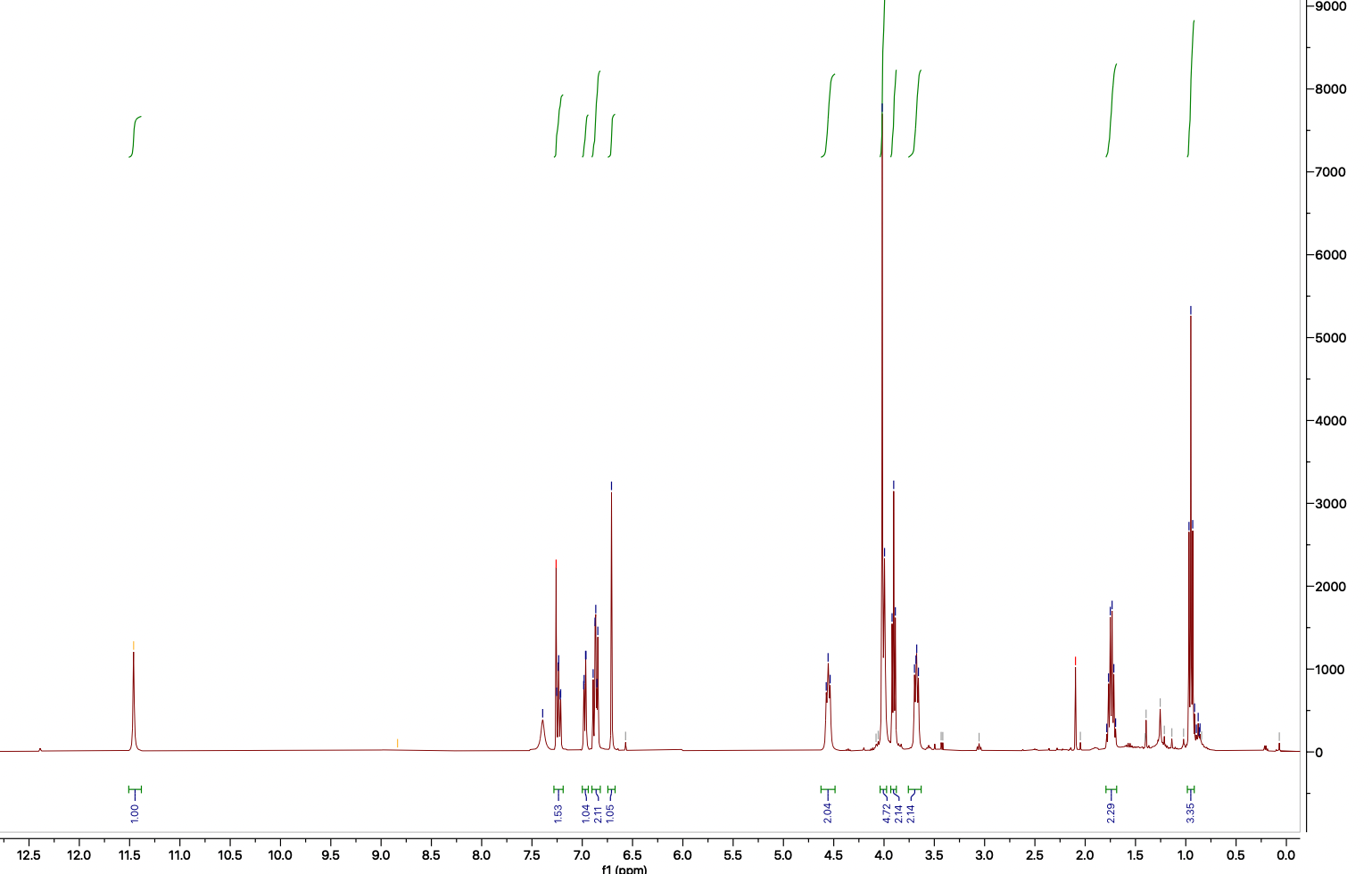
**

**methyl 3-chloro-4,6-dihydroxy-2-(2-(5-(2-propoxybenzyl)-1H-1,2,3-triazol-1-yl)ethyl)benzoate (42)**

**^13^C NMR**

**
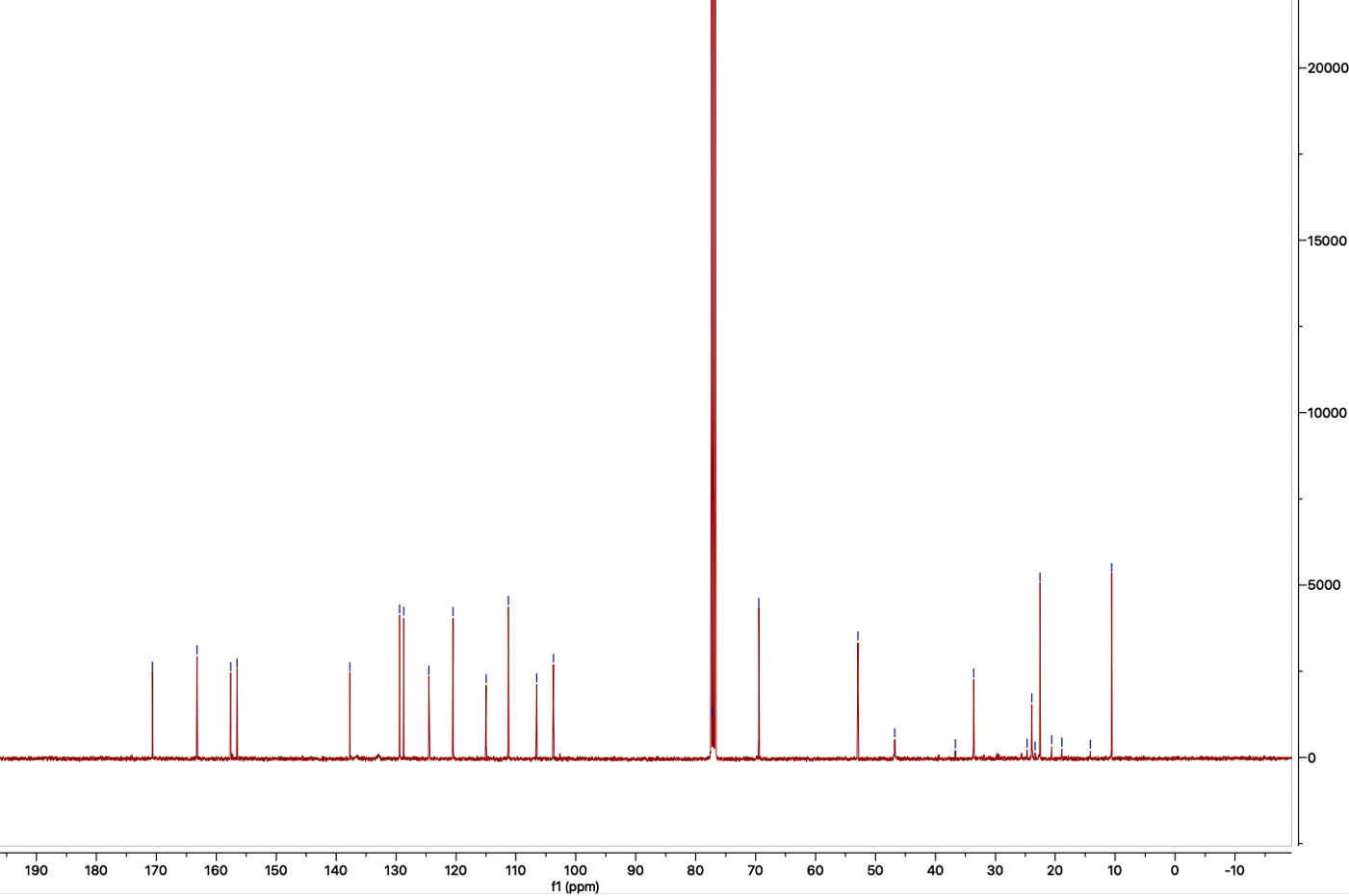
**

**methyl 3-chloro-2-(2-(5-(2-(ethylthio)benzyl)-1H-1,2,3-triazol-1-yl)ethyl)-4,6-dihydroxybenzoate (43)**

**^1^H NMR**

**
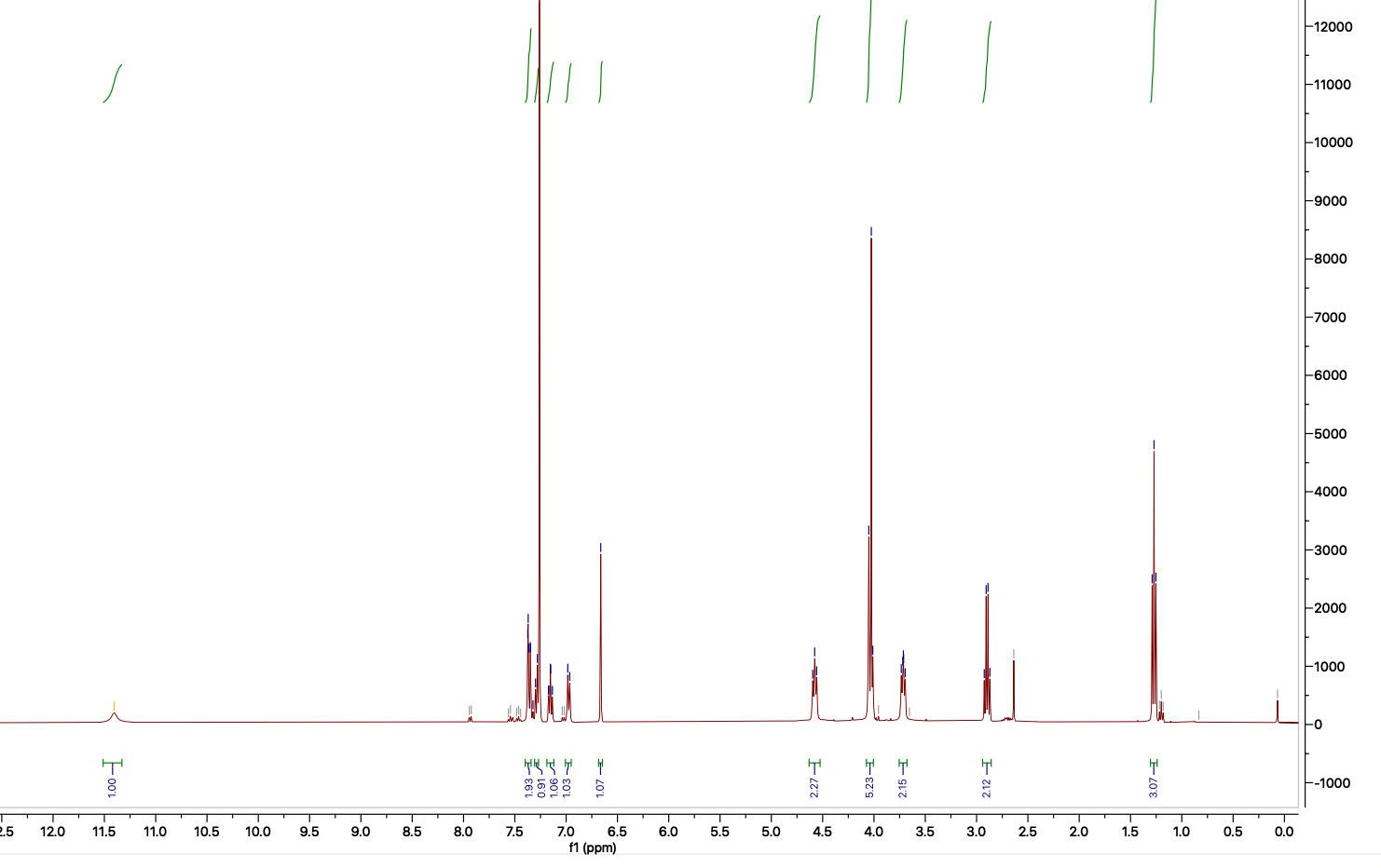
**

**methyl 3-chloro-2-(2-(5-(2-(ethylthio)benzyl)-1H-1,2,3-triazol-1-yl)ethyl)-4,6-dihydroxybenzoate (43)**

**^13^C NMR**

**
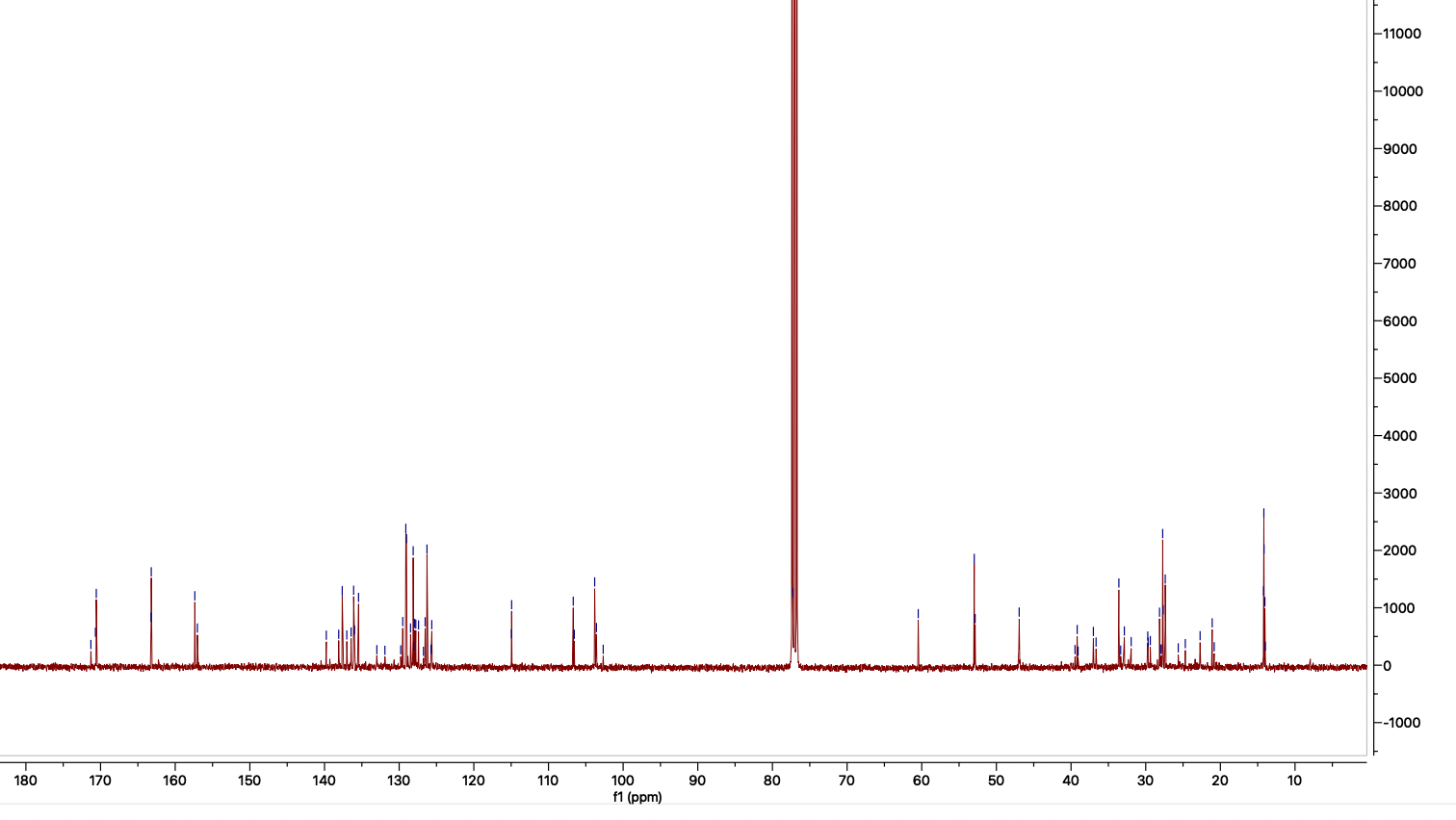
**

**methyl 3-chloro-4,6-dihydroxy-2-(2-(5-(2-(propylthio)benzyl)-1*H*-1,2,3-triazol-1-yl)ethyl)benzoate (44)**

**^1^H NMR**

**
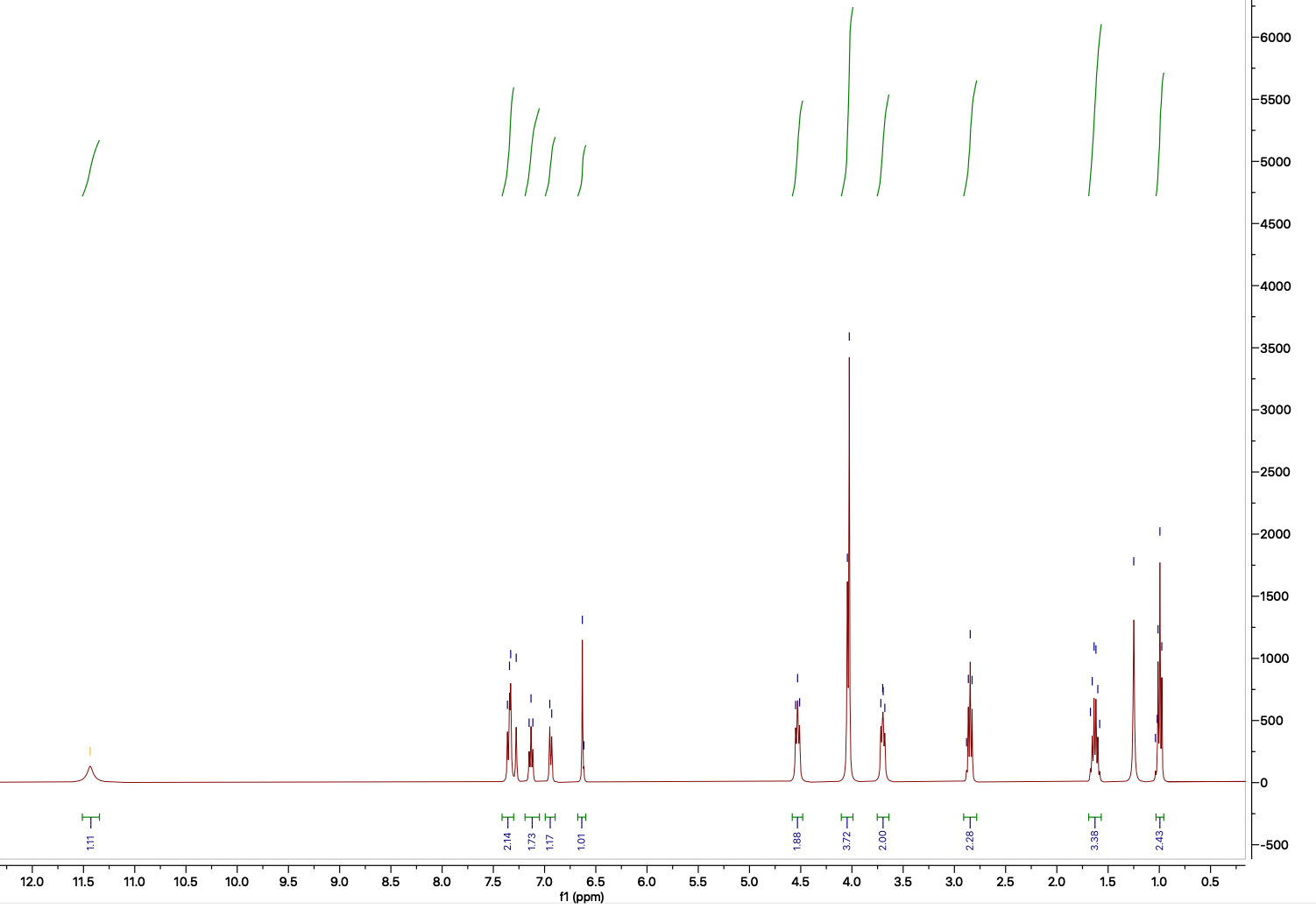
**

**methyl 3-chloro-4,6-dihydroxy-2-(2-(5-(2-(propylthio)benzyl)-1*H*-1,2,3-triazol-1-yl)ethyl)benzoate (44)**

**^13^C NMR**

**
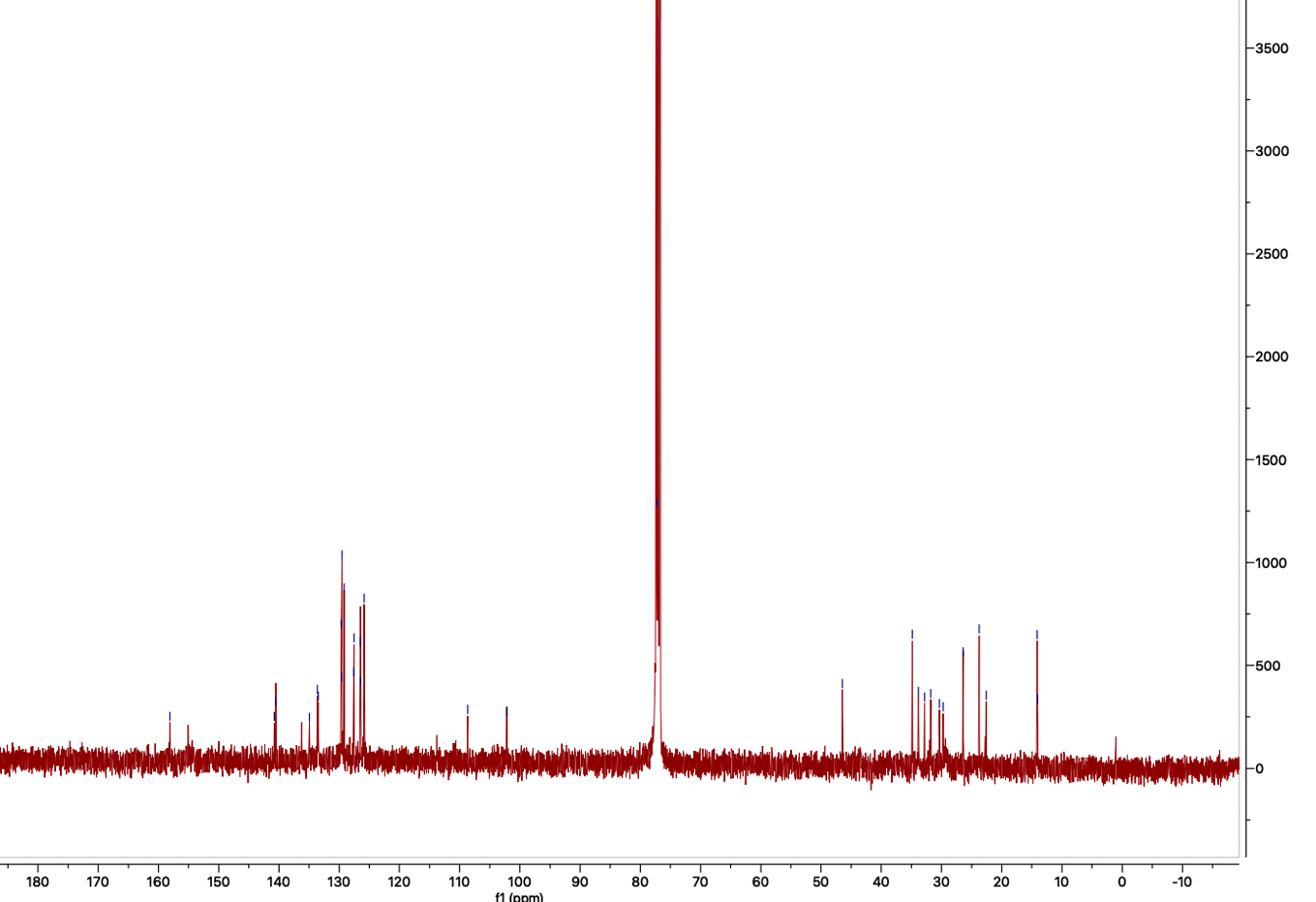
**

**methyl 3-chloro-4,6-dihydroxy-2-(2-(5-(2-(isopropylthio)benzyl)-1*H*-1,2,3-triazol-1-yl)ethyl)benzoate (45)**

**^1^H NMR**

**
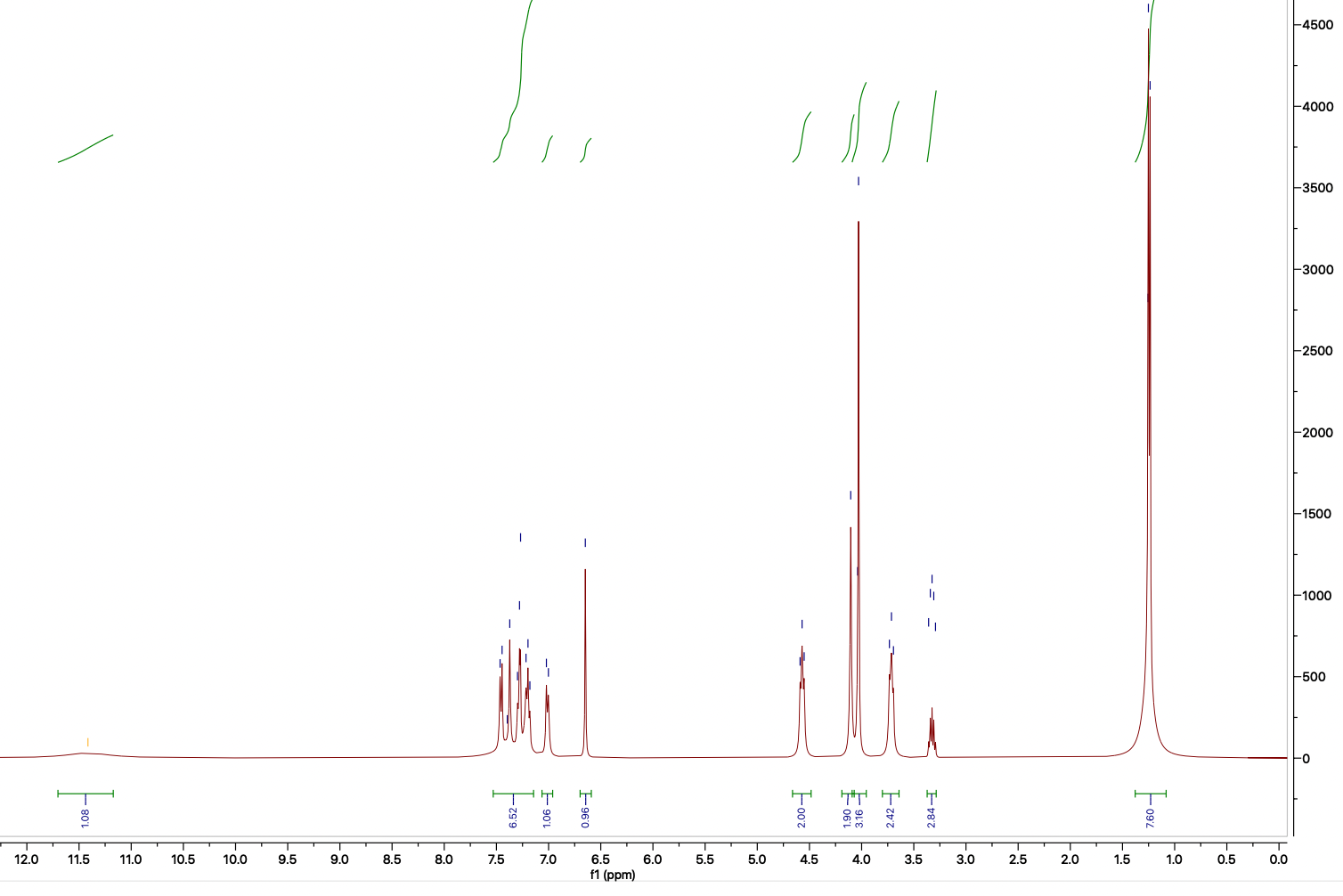
**

**methyl 3-chloro-4,6-dihydroxy-2-(2-(5-(2-(isopropylthio)benzyl)-1*H*-1,2,3-triazol-1-yl)ethyl)benzoate (45)**

**^13^C NMR**

**
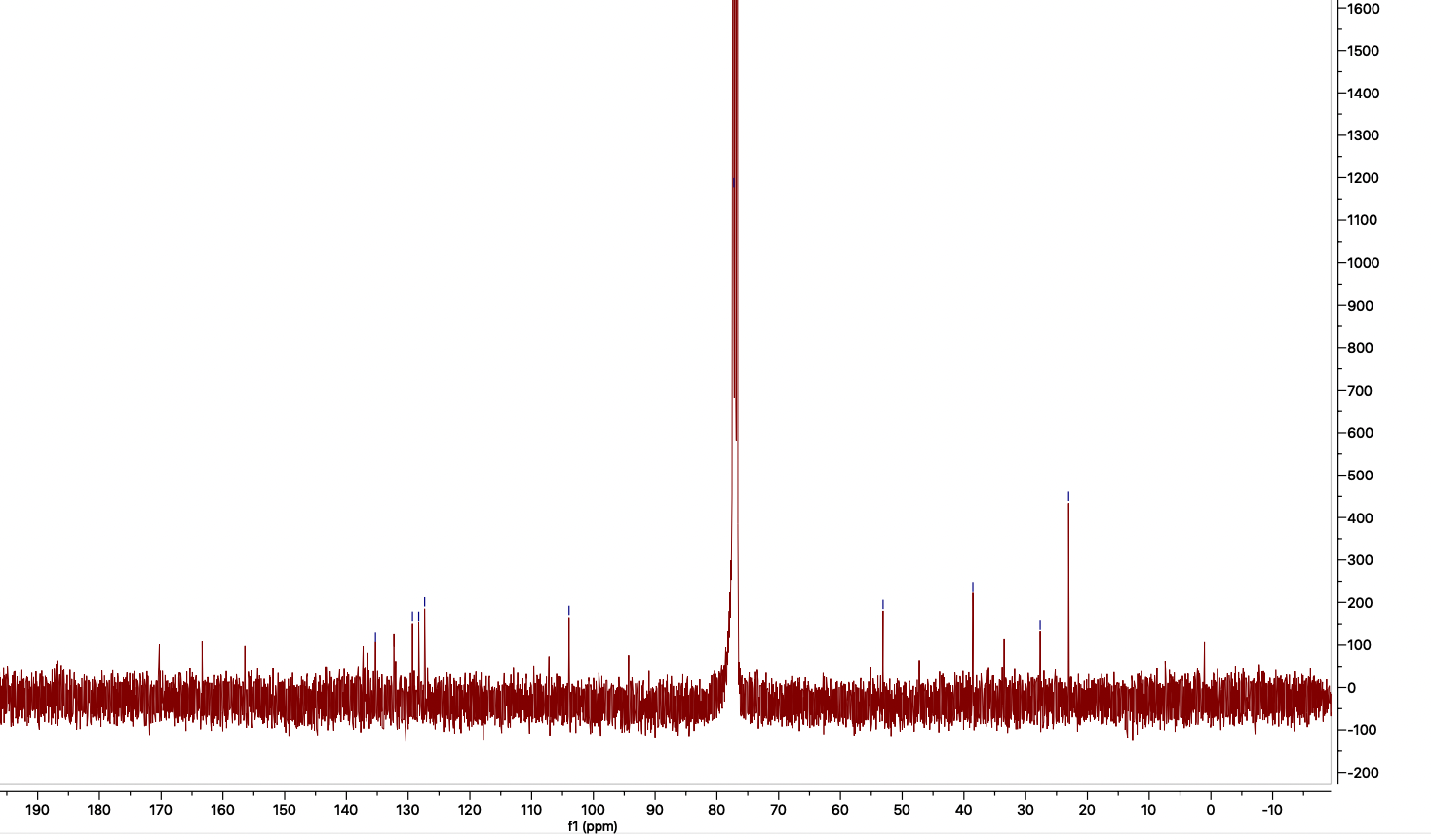
**

**methyl 3-chloro-2-(2-(5-(2-ethylbenzyl)-1H-1,2,3-triazol-1-yl)ethyl)-4,6-dihydroxybenzoate (46)**

**^1^H NMR**

**
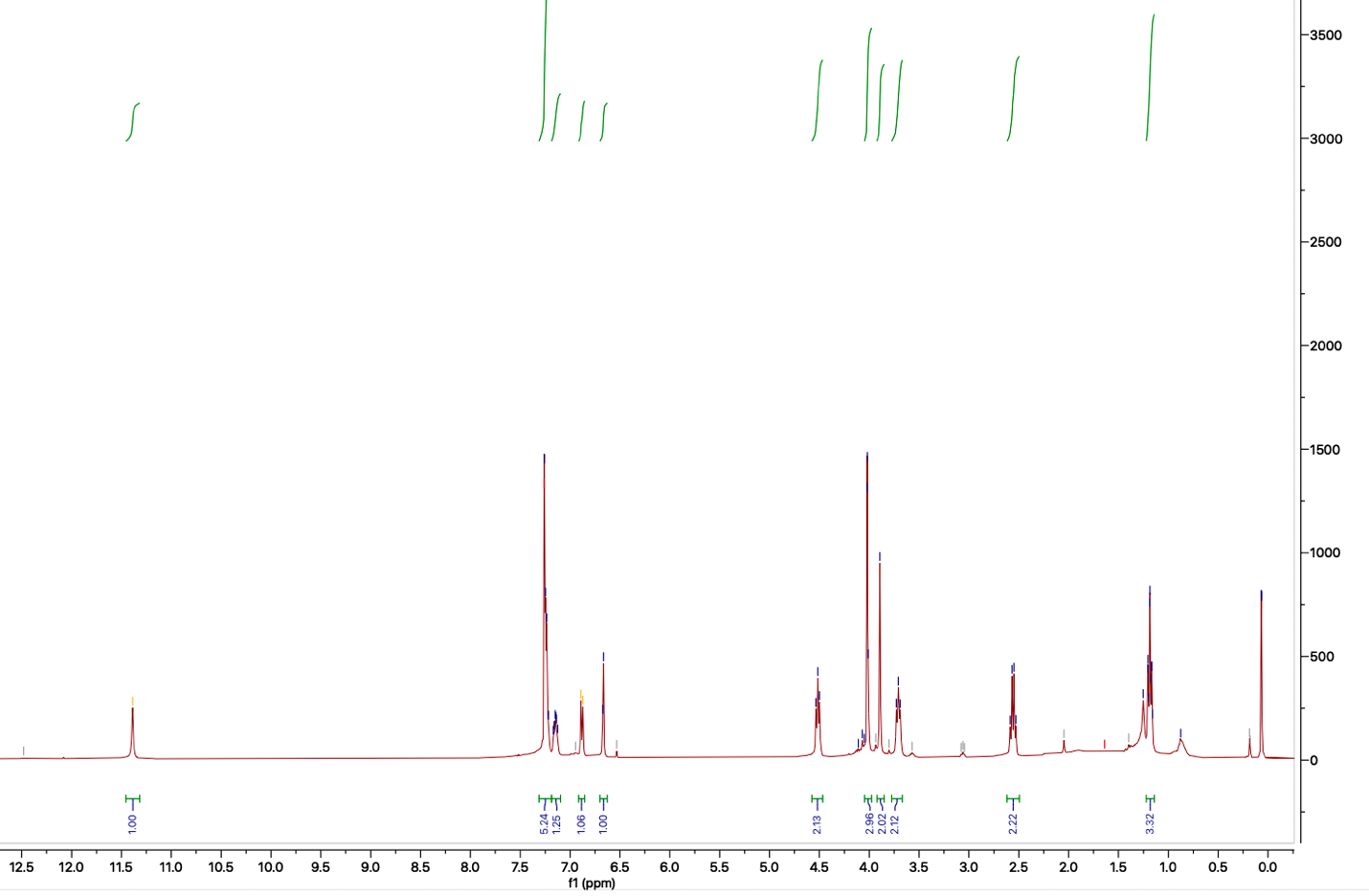
**

**methyl 3-chloro-2-(2-(5-(2-ethylbenzyl)-1H-1,2,3-triazol-1-yl)ethyl)-4,6-dihydroxybenzoate (46)**

**^13^C NMR**

**
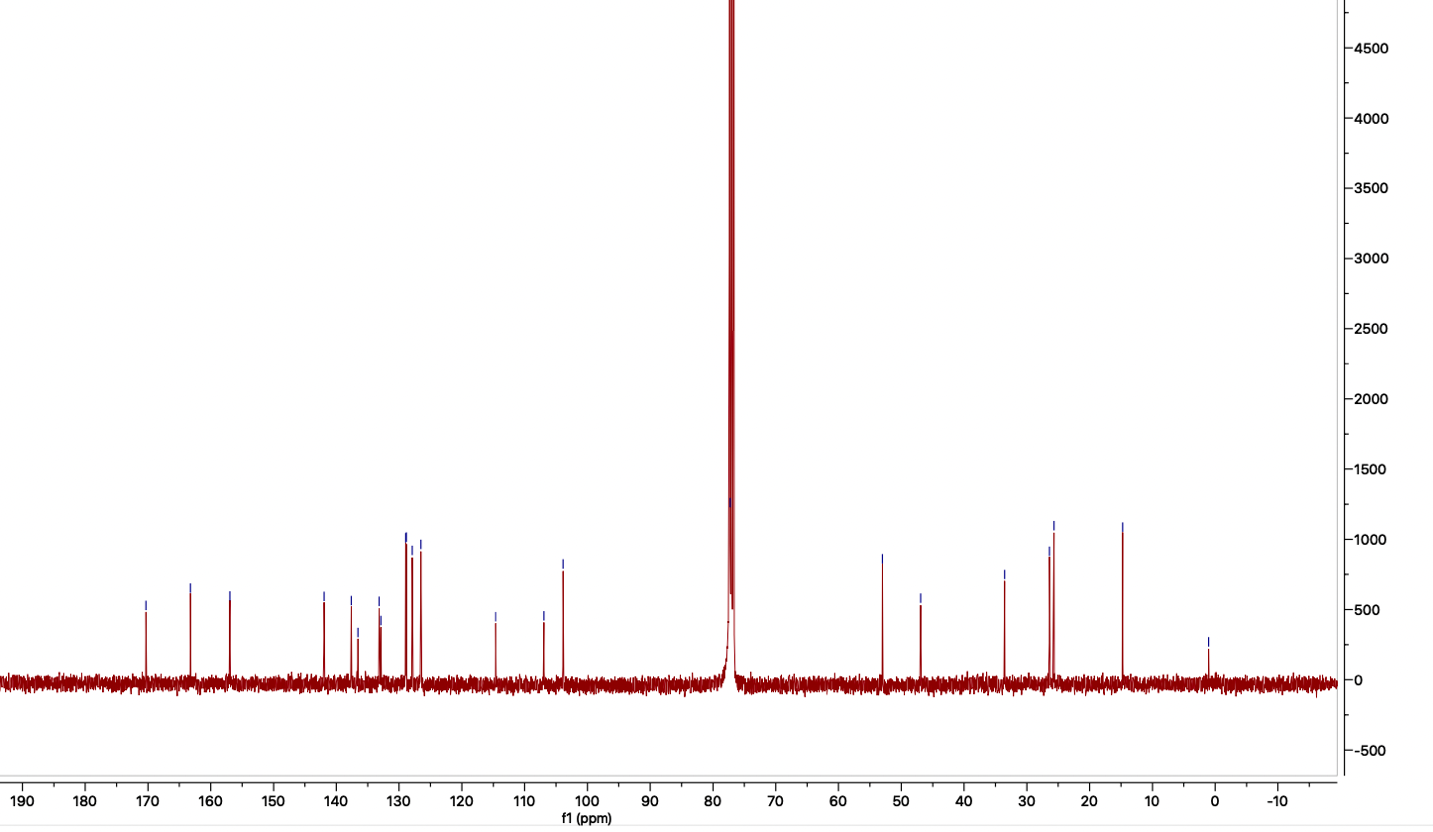
**

**methyl 3-chloro-4,6-dihydroxy-2-(2-(5-(2-propylbenzyl)-1H-1,2,3-triazol-1-yl)ethyl)benzoate (47)**

**^1^H NMR**

**^
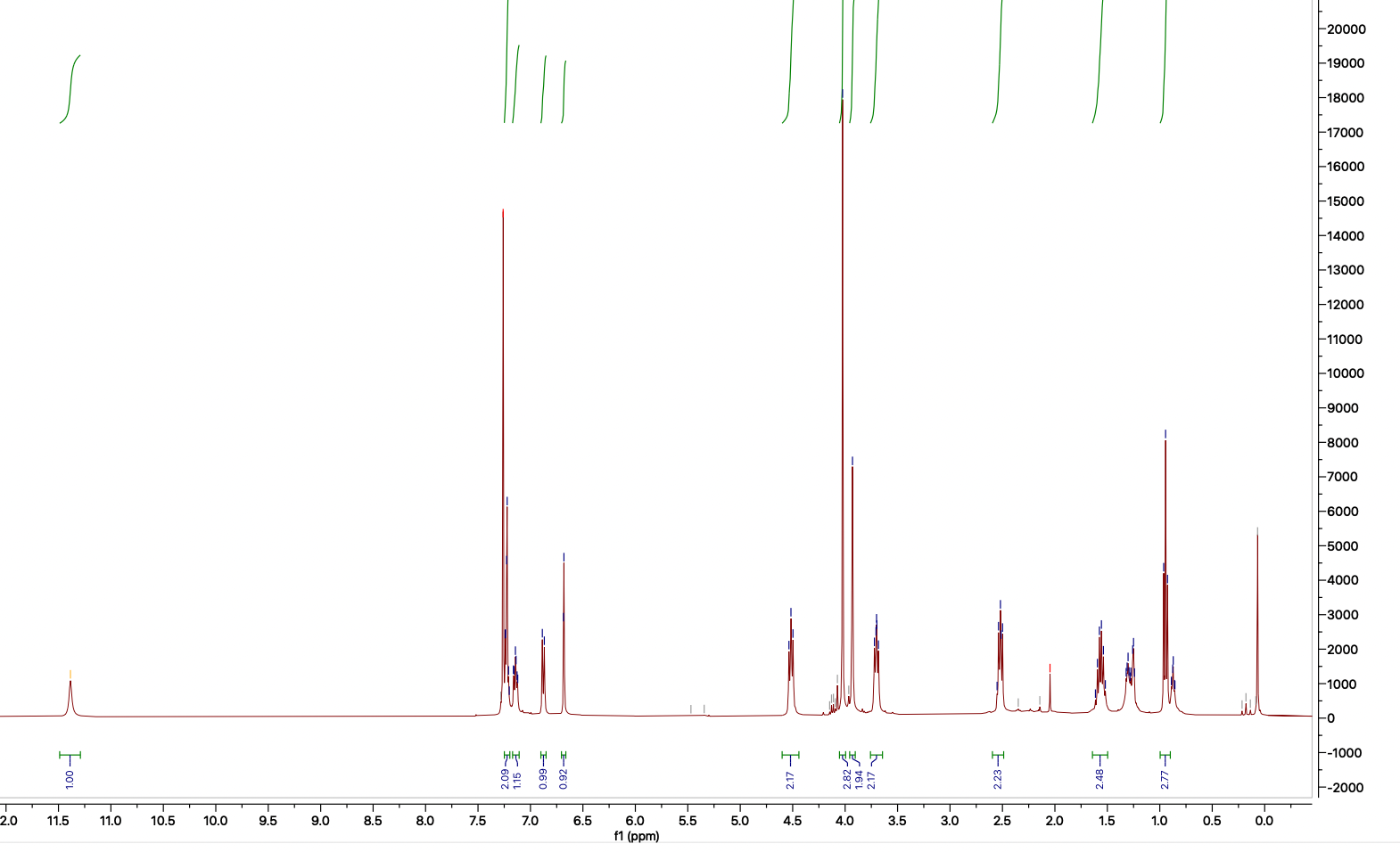
^**

**methyl 3-chloro-4,6-dihydroxy-2-(2-(5-(2-propylbenzyl)-1H-1,2,3-triazol-1-yl)ethyl)benzoate (47)**

**^13^C NMR**

**^
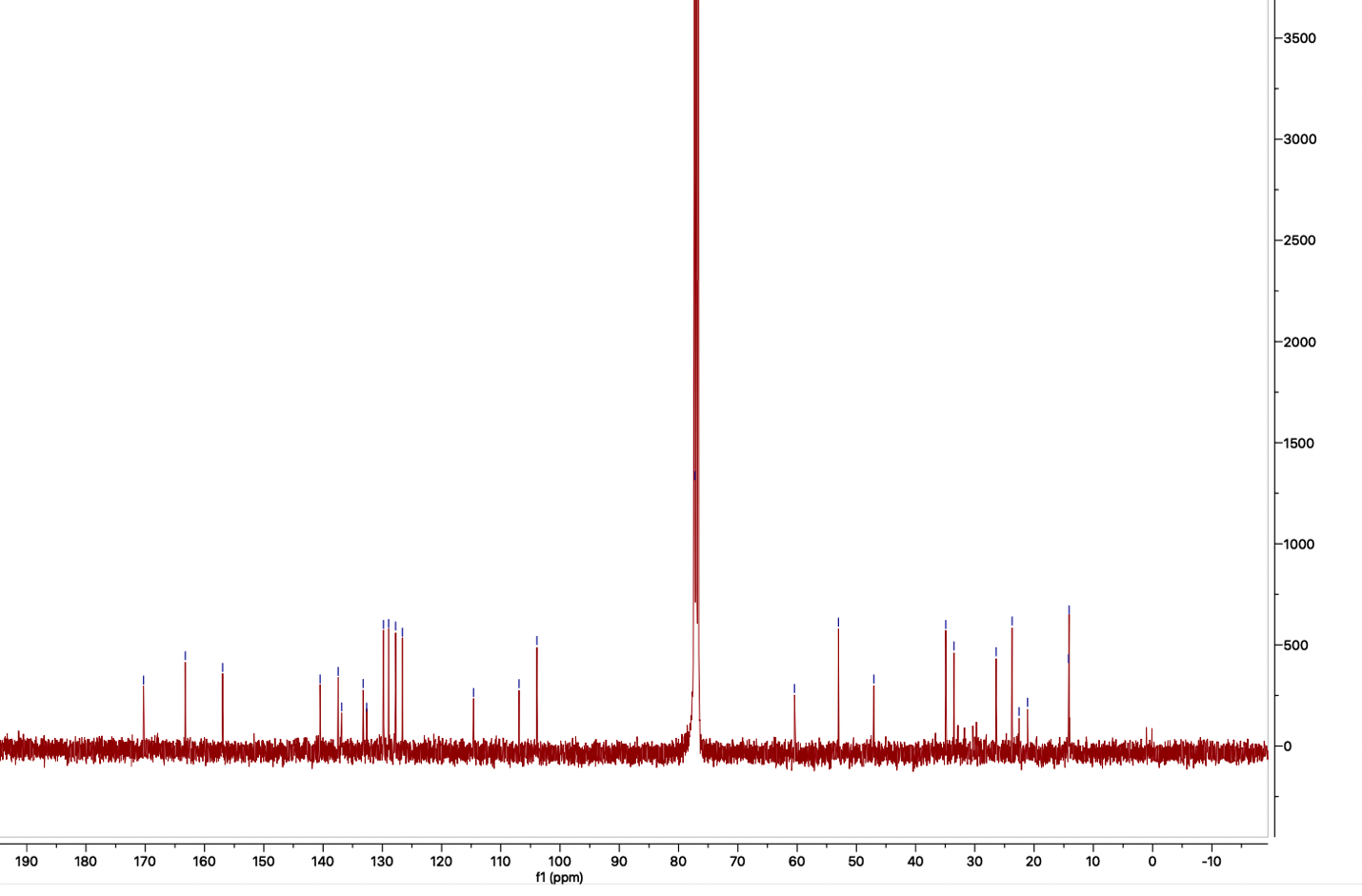
^**

**methyl 2-(2-(5-(2-butylbenzyl)-1*H*-1,2,3-triazol-1-yl)ethyl)-3-chloro-4,6-dihydroxybenzoate (48)**

**^1^H NMR**

**
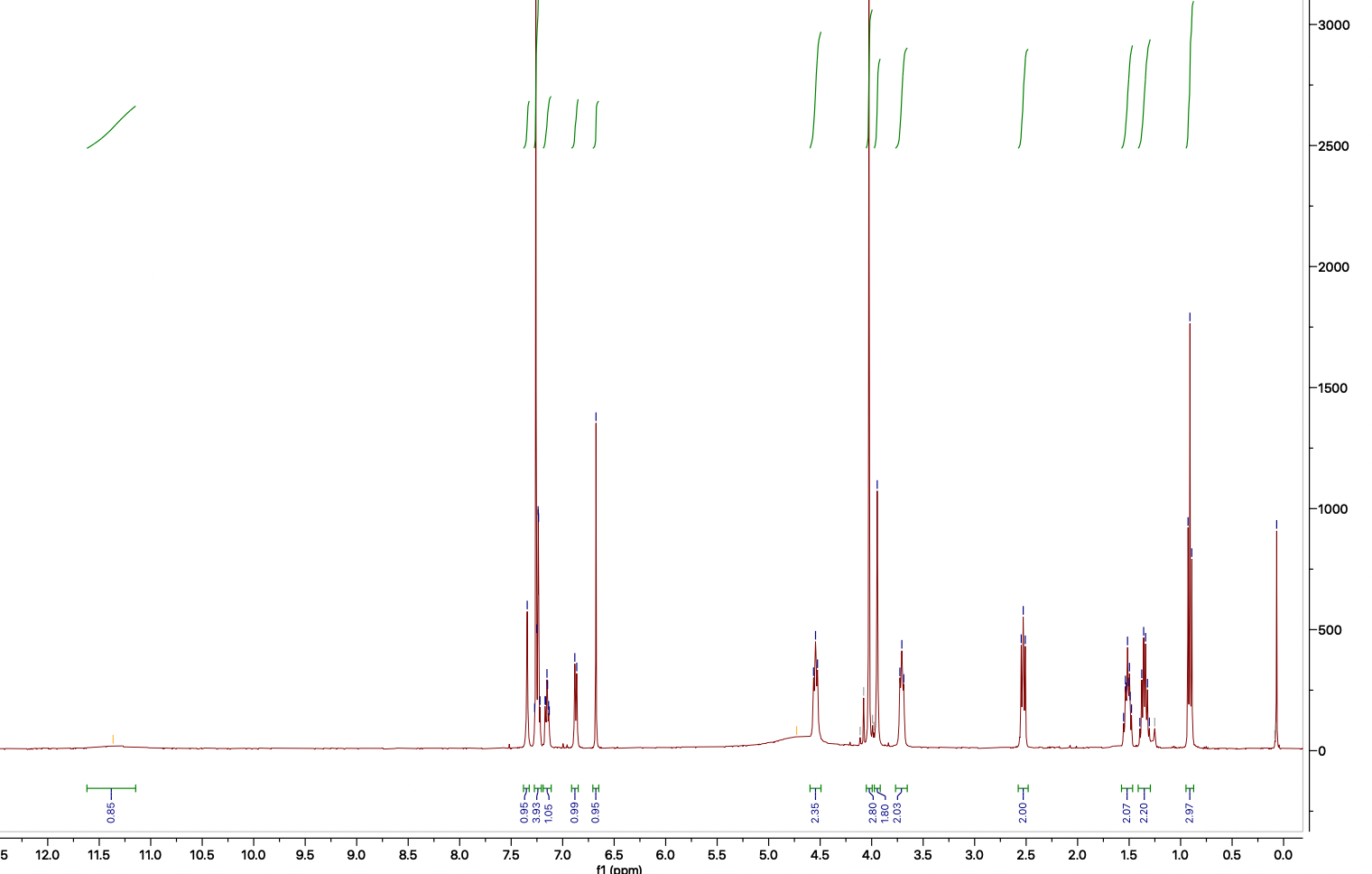
**

**methyl 2-(2-(5-(2-butylbenzyl)-1*H*-1,2,3-triazol-1-yl)ethyl)-3-chloro-4,6-dihydroxybenzoate (48)**

**^13^C NMR**

**
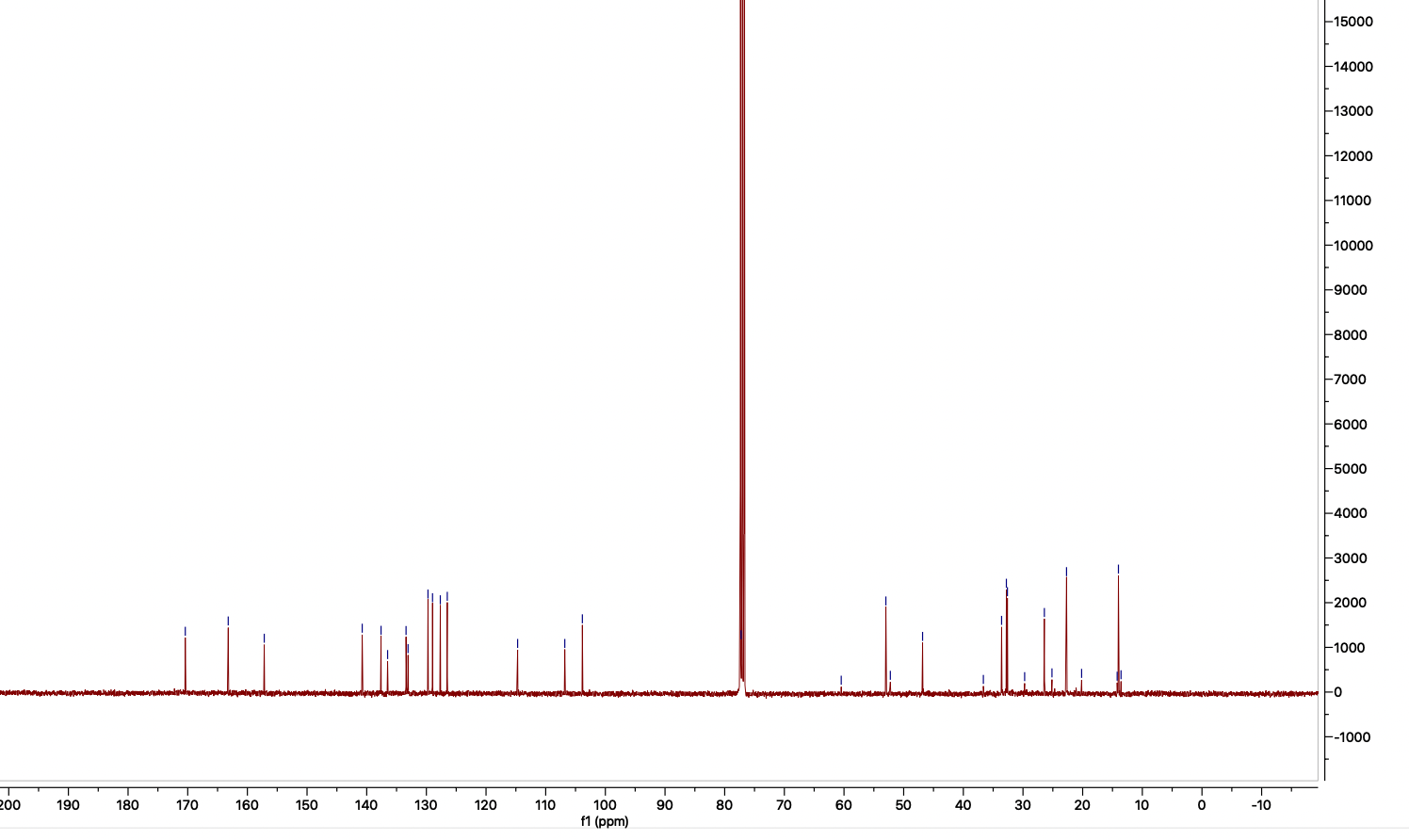
**

**methyl 3-chloro-4,6-dihydroxy-2-(2-(5-(2-isobutylbenzyl)-1H-1,2,3-triazol-1-yl)ethyl)benzoate (49)**

**^1^H NMR**

**
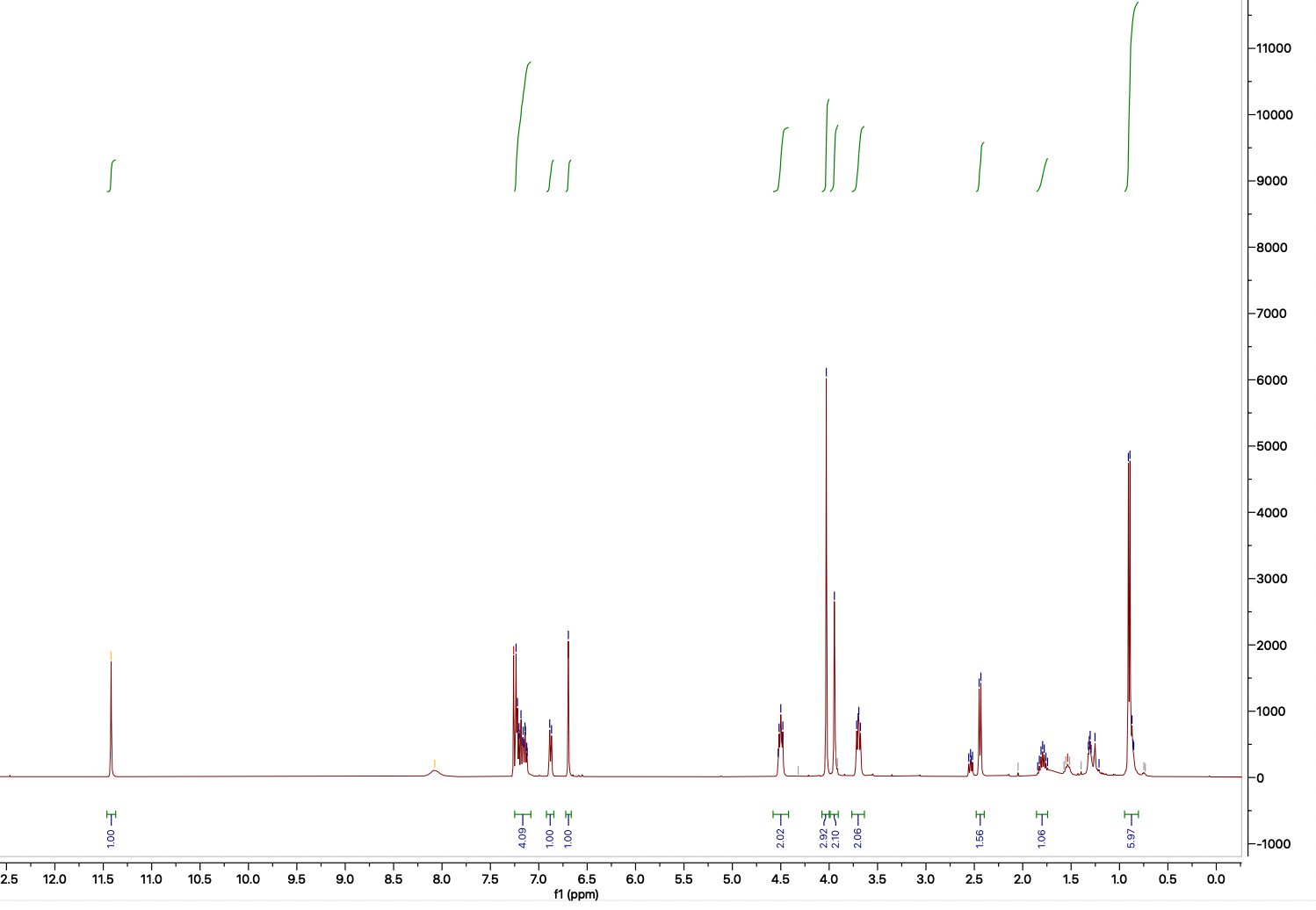
**

**methyl 3-chloro-4,6-dihydroxy-2-(2-(5-(2-isobutylbenzyl)-1H-1,2,3-triazol-1-yl)ethyl)benzoate (49)**

**^13^C NMR**

**
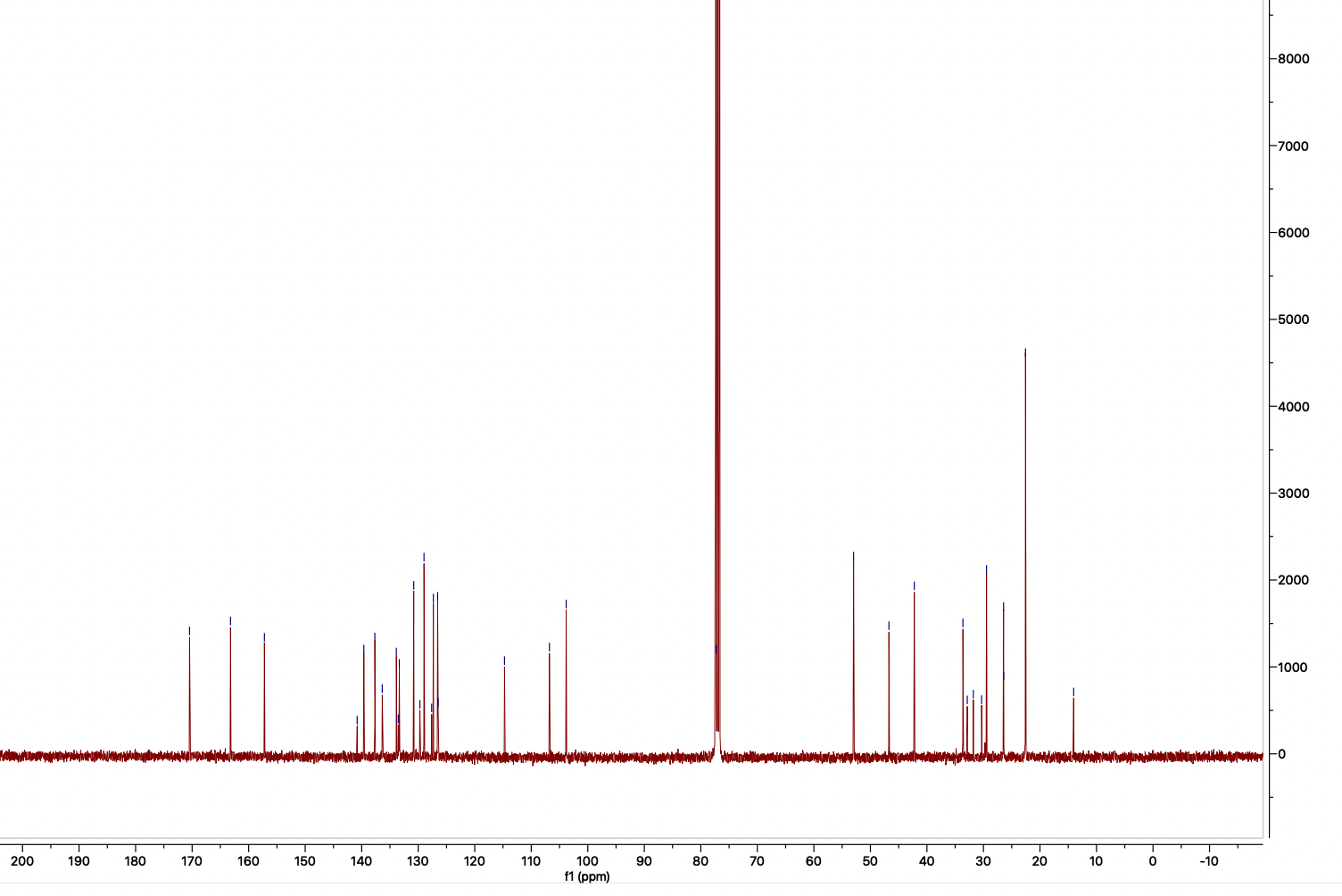
**

**methyl 3-chloro-4,6-dihydroxy-2-(2-(5-(2-(3,3,3-trifluoropropyl)benzyl)-1*H*-1,2,3-triazol-1-yl)ethyl)benzoate (50)**

**^1^H NMR**

**
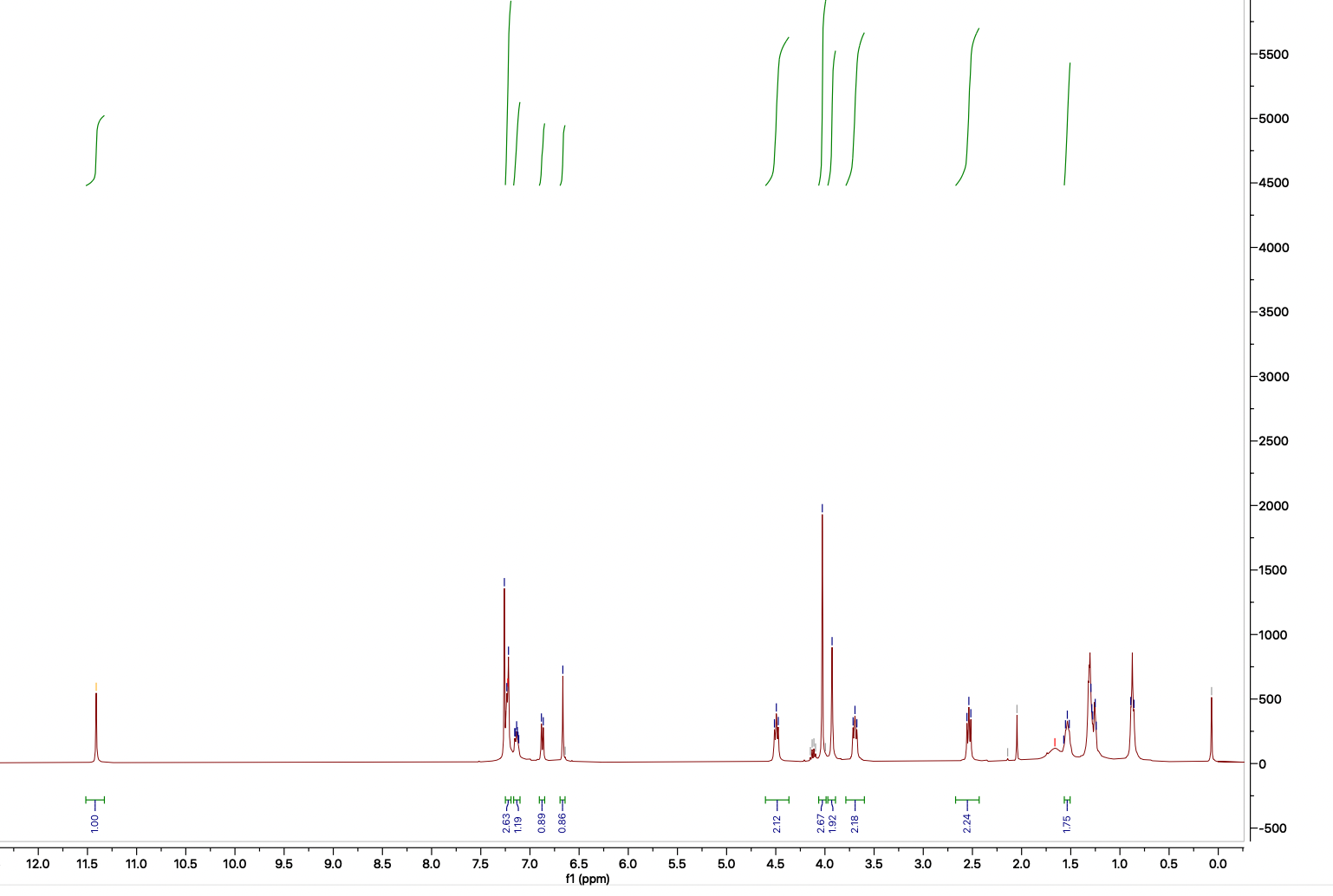
**

**methyl 3-chloro-4,6-dihydroxy-2-(2-(5-(2-(3,3,3-trifluoropropyl)benzyl)-1*H*-1,2,3-triazol-1-yl)ethyl)benzoate (50)**

**^13^C NMR**

**
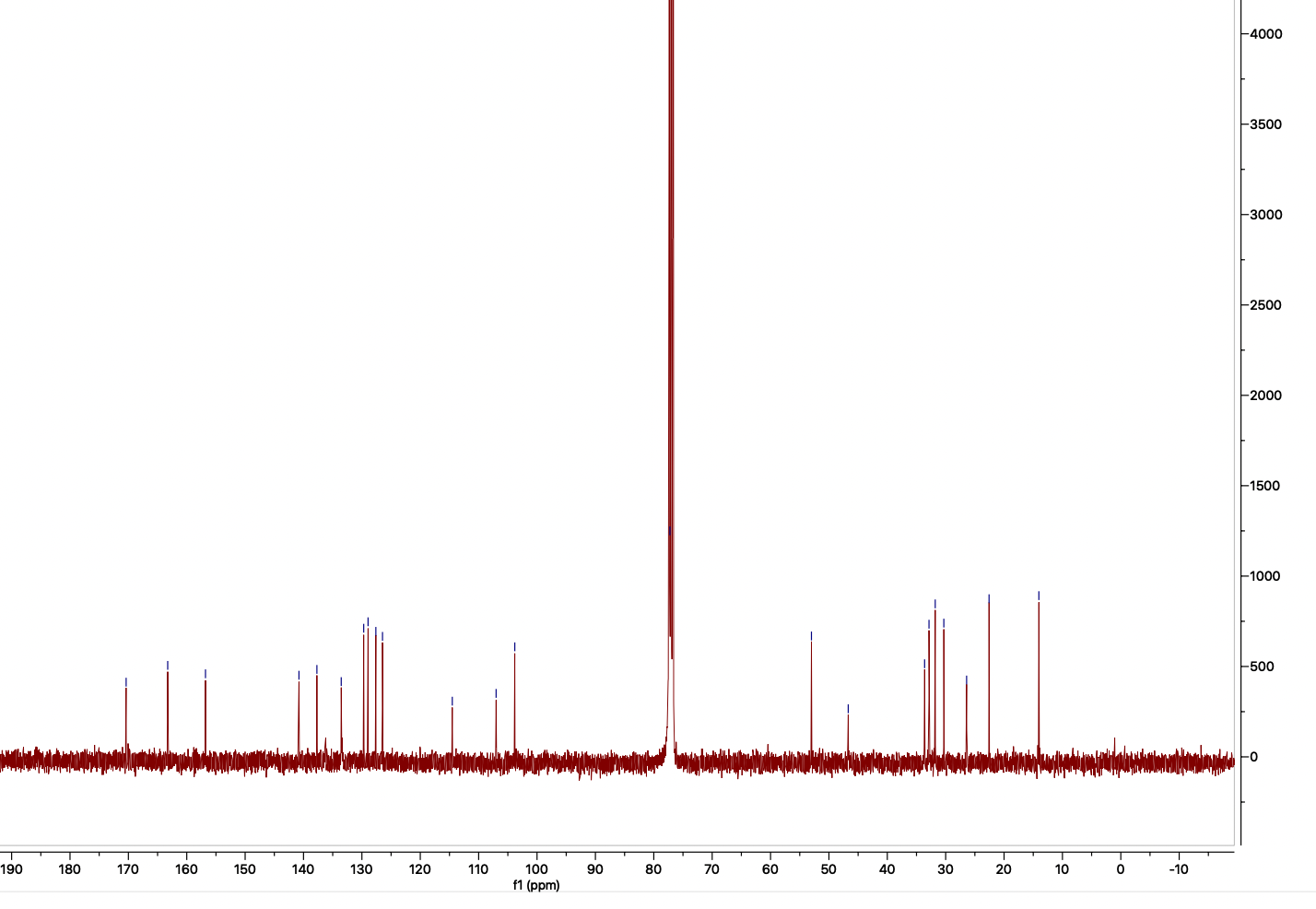
**
